# Antibacterial Activities of Predicted AMP in *Lactiplantibacillus plantarum* K9

**DOI:** 10.3390/ijms27156843

**Published:** 2026-07-30

**Authors:** Woo Young Bang, Kyu Ho Bang, A Yeong Cha, Se Hee Kim, Jin Hur, Sam Woong Kim

**Affiliations:** 1 National Institute of Biological Resources (NIBR), Environmental Research Complex, Incheon 22689, Republic of Korea; wybang@korea.kr; 2 Department of Pharmaceutical Engineering, Gyeongsang National University, Jinju 52725, Republic of Korea; khbang0095@gnu.ac.kr (K.H.B.); ayoung_912@naver.com (A.Y.C.); kshee7844@naver.com (S.H.K.); 3 Department of Veterinary Public Health, College of Veterinary Medicine, Jeonbuk National University, Iksan-Si 54596, Republic of Korea; 4 Agri-Food Bio Convergence Institute, Gyeongsang National University, Jinju 52725, Republic of Korea; 5 FARMIMs Co., Ltd., 55, Dongjin-Ro, Jinju 52725, Republic of Korea

**Keywords:** AMP, antibacteria, CAMP_R4_, genome, *L. plantarum*, transcriptome

## Abstract

This study aimed to identify novel antimicrobial peptides (AMPs) from *Lactiplantibacillus plantarum* K9, isolated from *Protaetia brevitarsis* seulensis larvae, using genome and transcriptome analyses. *L. plantarum* K9, among the isolated lactic acid bacteria from *P. brevitarsis* larvae, showed the strongest antimicrobial activity, with MIC_90_ values of 22 μL against *E. coli* and 21 μL against *S. aureus*. Approximately 54% of the antimicrobial activity was attributed to peptides or proteins. Genome analysis revealed a 3 Mb chromosome and three plasmids. Screening of hypothetical proteins using CAMP_R4_ identified 18 AMP candidates. Transcriptomic analysis showed that most AMP genes were more highly expressed in the stationary phase and were upregulated under low-pH conditions. Antimicrobial assays using synthetic peptides demonstrated that AMP4, 6, 7, 12-2, 14, and 18 exhibited strong activity against *S. aureus*, whereas AMP16 and 18 were effective against *E. coli*. After cloning each AMP and assessing its antimicrobial activity, the cloned AMPs exhibited overall greater antimicrobial activity against *S. aureus* than against *E. coli*. Among all candidates, AMP12 displayed the strongest antimicrobial activity and high specificity toward *S. aureus*. Structural analysis revealed a conserved α-helical region that is likely responsible for membrane-targeting antimicrobial activity. Collectively, these findings suggest that *L. plantarum* K9 AMP12 is a novel antimicrobial peptide that contributes to the strain’s antimicrobial properties.

## 1. Introduction

Lactic acid bacteria (LAB) are among the most widely used probiotic microorganisms. Representative LAB genera include *Aerococcus*, *Carnobacterium*, *Enterococcus*, *Lactobacillus*, *Lactococcus*, *Leuconostoc*, *Streptococcus*, *Tetragenococcus*, *Vagococcus*, and *Weissella*. In addition, numerous species belonging to the genera *Lactobacillus* and *Bifidobacterium* have been widely used as probiotics [1]. Among them, *Lactiplantibacillus plantarum* is one of the most extensively studied and commercially utilized probiotic species. As a facultatively heterofermentative bacterium, *L. plantarum* is relatively easy to culture and can adapt to a wide range of ecological niches, including fermented products, meat, plants, and the gastrointestinal tract [2]. Owing to its remarkable ecological versatility, *L. plantarum* has been reported to confer various health benefits, including cholesterol-lowering effects, improved vascular endothelial function, and reduction in postprandial glucose and HbA1c levels in individuals with prediabetes or type 2 diabetes [3,4,5].

*L. plantarum* can produce antimicrobial peptides (AMPs), including plantaricins and holins. Together with organic acids, these antimicrobial compounds are thought to play important roles in controlling food spoilage, animal diseases, and infections caused by antibiotic-resistant bacteria [6]. Recently, AMPs from diverse biological sources have attracted increasing attention as potential alternatives to conventional antibiotics [7]. Newly identified AMPs are continuously archived in specialized databases, such as the Antimicrobial Peptide Database (APD; http://aps.unmc.edu/AP) and the Database of Research on Antimicrobial Peptides (DRAMP; http://dramp.cpu-bioinfor.org/), which serve as valuable resources for the identification and development of novel therapeutic candidates [8,9]. However, naturally occurring AMPs are often generated at low levels, limiting their practical application. To overcome this limitation, various production strategies, including chemical synthesis, recombinant DNA technology, and heterologous expression systems, have been developed to improve AMP yield [10]. In addition, several approaches, such as the incorporation of noncanonical amino acids, peptide cyclization, terminal or side-chain modifications, and nanoparticle-based formulations, have been employed to enhance AMP stability and prevent rapid degradation in biological environments [10].

In this study, to isolate lactic acid bacteria with more beneficial functions for humans, *L. plantarum* K9 originated from *P. brevitarsis* seulensis was screened and isolated for its high antimicrobial activity. The isolated *L. plantarum* K9 was subjected to genome and transcriptome analyses, with the transcriptome analyzed under different pH conditions and growth phases. In addition, based on the results of genome analysis, candidate AMPs were predicted using the CAMP_R4_ program, and their antimicrobial activities were evaluated through oligopeptide synthesis and gene cloning.

## 2. Results

### 2.1. Antimicrobial Activity of L. plantarum K9 Isolated from the Gut of P. brevitarsis Larvae

**Figure 1:**
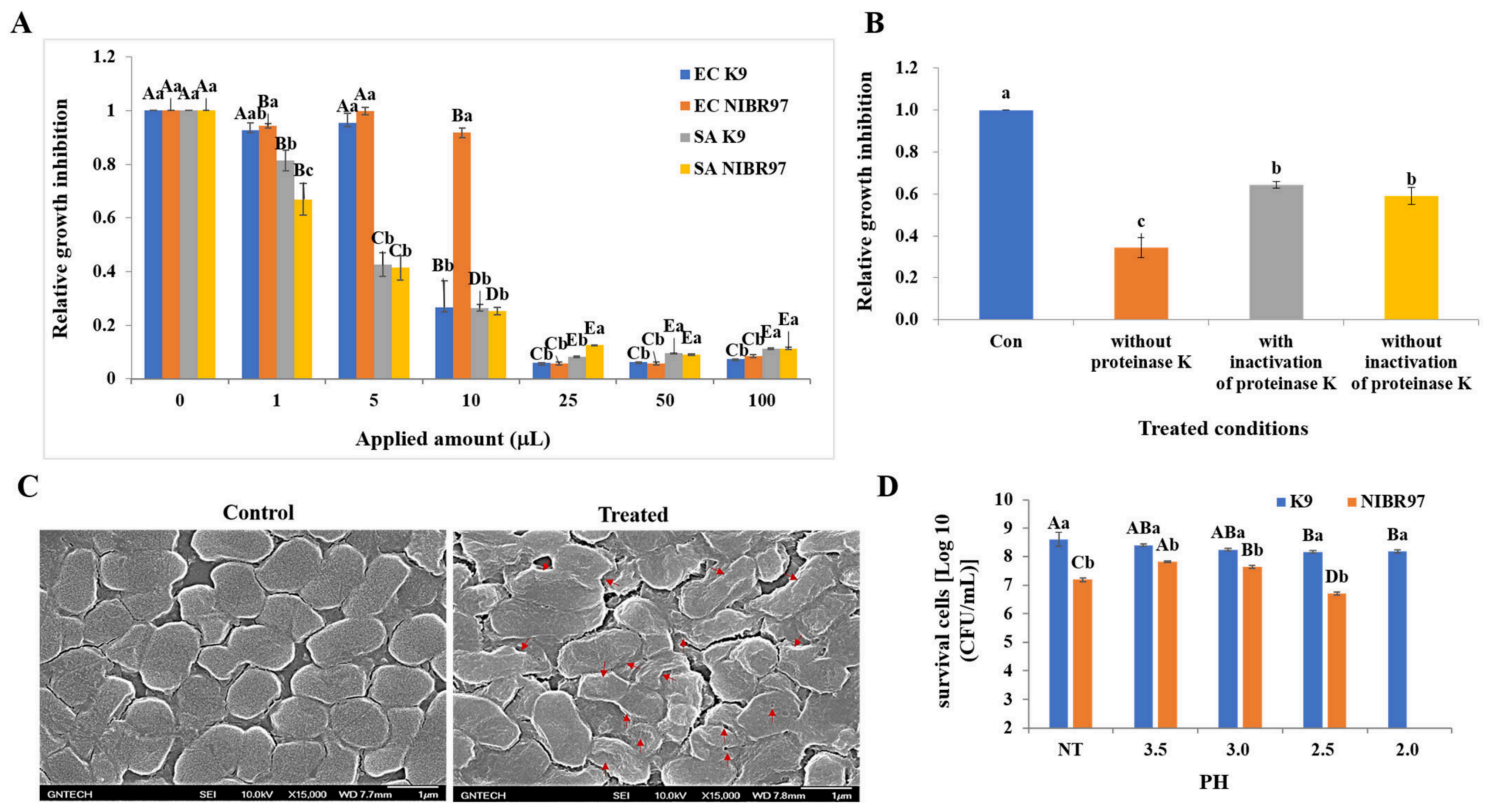
Antibacterial activities of *L. plantarum* K9 cell-free broths. (**A**) Antibacterial activities of *L. plantarum* K9 and NIBR97 cell-free culture broths at different broth concentrations; (**B**) changes in antibacterial activity following proteinase K treatment; (**C**) SEM images of *E. coli* treated with the cell-free culture broth of *L. plantarum* K9; (**D**) acid tolerances of *L. plantarum* K9 and NIBR97. The cell-free culture broths were prepared by centrifugation and filtration of *L. plantarum* K9 culture broth. Proteinase K was applied at a final concentration of 80 μg/mL and incubated for 1 h at 37 °C. Different lowercase and uppercase letters indicate significant differences among treatments (*p* < 0.05). EC and SA denote *E. coli* and *S. aureus*, respectively. Con; non-treated with proteinase K, NT; non-treated. Arrows in (**C**) indicate the locations of holes formed as results of antimicrobial activity.

The antimicrobial activity of cell-free culture broth of the *L. plantarum* K9 against *Escherichia coli* (EC) and *Staphylococcus aureus* (SA) is shown in Figure 1. The broth of *L. plantarum* K9 exhibited comparable antimicrobial activities against both EC and SA, with MIC_90_ values of 21.9 and 21.0 µL/mL, respectively (Table 1 and Supplementary Material Figures S1–S6). When compared with *L. plantarum* NIBR97 [11], *L. plantarum* K9 showed slightly stronger antimicrobial activity (Figure 1A and Table 1). *L. plantarum* is generally capable of facultative heterofermentative metabolism; however, when glucose is available as the primary carbon source, it predominantly produces lactic acid. Consequently, lactic acid is generated at high levels, resulting in strong antimicrobial activity associated with its production [12]. Therefore, to determine whether the antimicrobial activity observed in this study was associated with AMPs, the cell-free broth was treated with proteinase K. As shown in Figure 1B, proteinase K treatment reduced the antimicrobial activity by 41.5~46.3%. These results suggest that proteinase K-sensitive substances contribute to the observed antimicrobial activity. Such substances are presumed to be AMPs, proteins, or their derivatives. To further investigate the mechanism underlying the antimicrobial activity, scanning electron microscopy (SEM) was performed for the cell-free broth. As shown in Figure 1C, the treated cells with cell-free broth exhibited pore-like structures and disruptions in the cell envelope.

On the other hand, to evaluate the probiotic potential of *L. plantarum* K9 based on its acid and bile tolerance, acid and bile salt tolerance assays were performed and compared with those of *L. plantarum* NIBR97, which has previously been reported to exhibit strong antimicrobial and antiviral activities [11]. In the bile acid tolerance assay, no significant difference was observed between the two strains [Supplementary Material Figure S7]. However, with respect to acid tolerance, *L. plantarum* K9 exhibited clear tolerance at pH 2.0, whereas *L. plantarum* NIBR97 showed no detectable tolerance under the same condition (Figure 1D).

### 2.2. Genome Analysis and AMP Prediction Results

**Figure 2:**
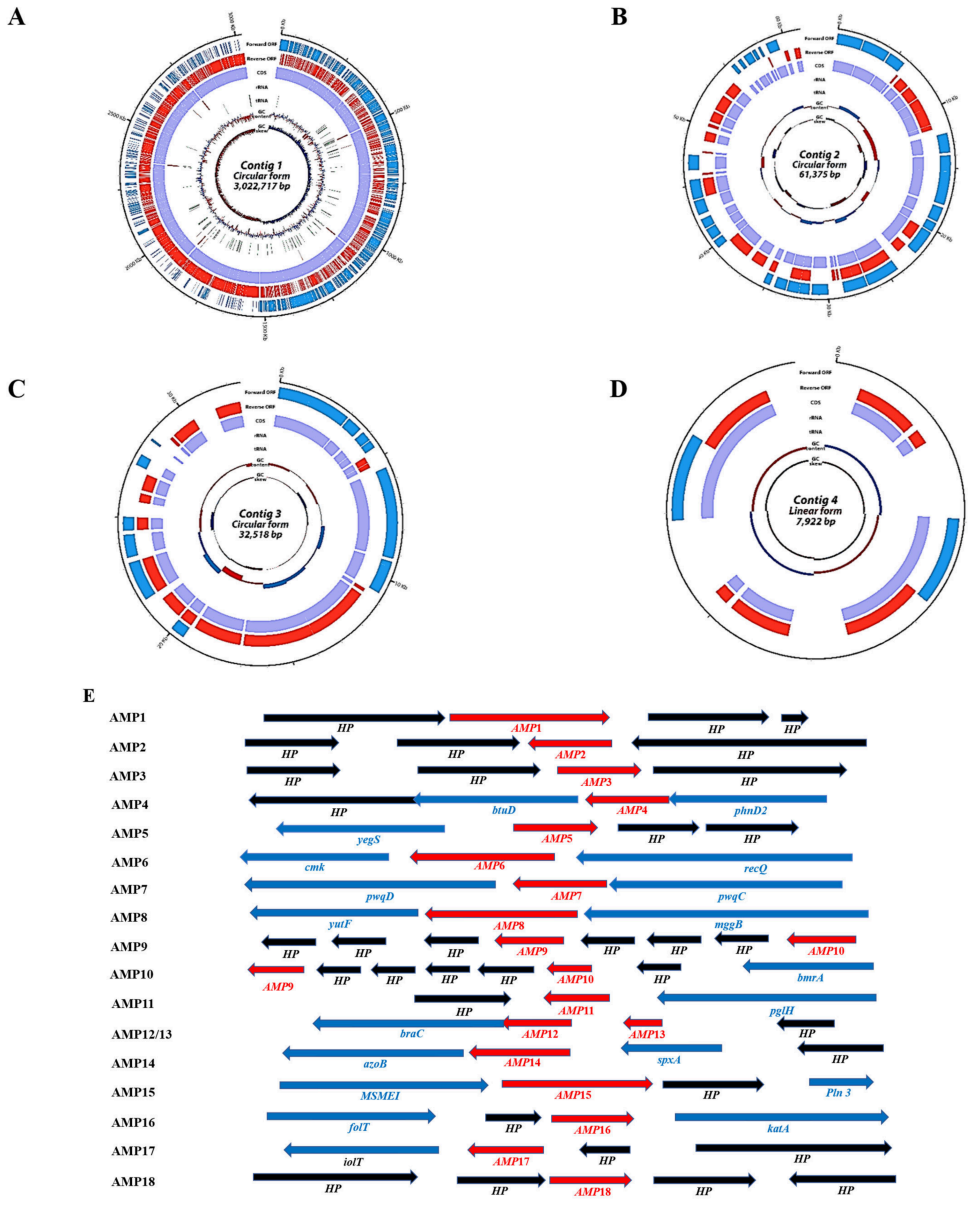
Classification according to contig of *L. plantarum* K9. (**A**) Contig 1, (**B**) contig 2, (**C**) contig 3, (**D**) contig 4, (**E**) arrangements of flanking genes for each AMPs (refer to Supplementary Material Figures S50–S53). The genome of *L. plantarum* K9 consisted of four separate contigs, including three circular replicons and one linear replicon. The contigs were 3,022,717, 61.375, 32,518, and 7922 bps in length, respectively. rRNA and tRNA genes were identified only in contig 1. *HP*: hypothetical protein; *btuD*: Vitamin B12 import ATP-binding protein BtuD; *phnD2*: putative ABC transporter phosphonate/phosphite binding protein PhnD2; yegS: lipid kinase YegS; *cmK*: cytidylate kinase; *recQ*: ATP-dependent DNA helicase RecQ; *yutF*: acid sugar phosphatase; *mggB*: mannosylglucosyl-3-phosphoglycerate phosphatase; *bmrA*: multidrug resistance ABC transporter ATP-binding/permease protein BmrA; *pglH*: GalNAc-α-(1→4)-GalNAc-α-(1→3)-diNAcBac-PP-undecaprenol α-1,4-N-acetyl-D-galactosaminyltransferase; *braC*: leucine-, isoleucine-, valine-, threonine-, and alanine-binding protein; *azoB*: NAD(P)H azoreductase; *spxA*: regulatory protein Spx; *MSMEI*: putative oxidoreductase/MSMEI_2347; *pln 3*: plantaricin 3; folT: folate transporter FolT; *katA*: vegetative catalase; *iolT*: major myo-inositol transporter IolT.

Genomic analysis was performed to investigate the presence of additional AMPs beyond well-known-antimicrobial compounds, such as plantaricin and holin, in *L. plantarum* K9, which exhibited relatively strong antimicrobial activity. The genome of *L. plantarum* K9 consisted of four contigs, with contig 1 corresponding to the chromosome and measuring 3,022,717 bp in length (Figure 2A and Figure S50). Contigs 2 and 3 were composed of 61,375 bp and 32,518 bp in length, respectively, and identified as circular plasmids (Figure 2B,C and Figures S51 and S52). Contig 4 was 7922 bp in length and was predicted to be a linear plasmid (Figure 2D and Figure S53). Because plasmids are generally circular DNA molecules, the linear structure of contig 4 may have resulted from sequence loss or incomplete assembly during genome reconstruction, and this possibility cannot be excluded.

Candidate AMPs in *L. plantarum* K9 were predicted from hypothetical proteins identified in the genome using the CAMP_R4_ program. As a result, a total of 18 candidate AMPs were identified from hypothetical protein sequences (Figure 2E and Figures S7–S49). Predicted AMPs showing more than 50% positive compared to previously reported AMPs were preferentially selected (Table 2, Table 3, Table 4 and Table 5). The flanking regions surrounding the candidate AMP genes were predominantly composed of hypothetical protein-encoding genes (Figure 2E). AMP9 and AMP10 were separated by three hypothetical protein genes, whereas AMP12 and AMP13 were located adjacent to each other. AMP9 and AMP10 shared identical amino acid sequences, and the three intervening genes also exhibited repetitive patterns with identical amino acid sequences. Among the candidate AMPs, AMP1, AMP2, AMP12, AMP16, and AMP17 were predicted to contain signal sequences. The sequence identity of the candidate AMPs to previously reported AMPs ranged from approximately 32% to 63%, whereas sequence positivity ranged from 50% to 86%. The peptide length varied from 14 to 62 amino acids, and the predicted isoelectric point (pI) ranged from 4.9 to 12.61. Notably, most candidate AMPs exhibited alkaline pI values.

### 2.3. Results of Transcriptome Analysis

The transcriptomic analysis results of the candidate AMP genes under different conditions are summarized in Table 2, Table 3 and Table 4. Across all genes, a total of 228 exhibited expression changes at least twofold in response to pH variation. Among these genes, upregulated genes were more frequently observed than downregulated genes under acidic conditions. Compared with the control condition (pH 3.5), substantial transcriptional changes were observed at pH 3.0 and 2.5, but no significant differences were detected between both pHs (Table 3). These findings suggest that numerous genes associated with acid tolerance undergo transcriptional regulation in response to decreasing pH value.

In contrast, gene expression changes associated with growth phase were much less pronounced than those induced by pH variation, with only four upregulated and seven downregulated genes identified (Table 3). Analysis of AMP gene expression according to growth phase revealed that AMP1, AMP4, AMP11, AMP12, and AMP15 were upregulated during the exponential phase, whereas the remaining AMP genes exhibited the opposite trend (Table 4). Among the candidate AMP genes, AMP18 showed the highest level of differential expression, with a 2.29-fold change, whereas AMP3 and AMP6 exhibited only minor expression changes of 1.14- and 1.10-fold, respectively. In contrast, AMP10 expression was not detected during the exponential phase, and AMP13 was not expressed under any of the conditions examined.

Changes in AMP gene expression in response to pH variation were much more pronounced than those associated with growth phase (Table 5). Expression of the AMP18 gene increased 15.51-fold at pH 2.5 compared with the control condition. At pH 3.0, AMP18 expression also increased 4.03-fold relative to the control, and a 3.85-fold difference was observed between pH 2.5 and pH 3.0. Overall, greater changes in gene expression were observed when comparing the control condition with pH 2.5 than with pH 3.0. Although relatively small differences were detected between pH 2.5 and pH 3.0, substantial expression changes were observed for AMP2, AMP12, and AMP18. Notably, expressions of AMP9 and AMP10 were not detected under the control condition, and AMP10 expression detected only at pH 3.0. Consistent with the results of the growth phase-dependent expression analysis, AMP13 was not expressed under any of the tested pH conditions, suggesting that it may not be a functional gene.

### 2.4. Evaluation of the Antimicrobial Activity of Synthesized AMPs

Peptide synthesis was performed for regions exhibiting homology to known AMPs. However, seven AMPs, AMP1~3, AMP5, AMP8, AMP10, and AMP15, could not be synthesized (Table 6). Among the synthesized peptides, AMP11-1 and AMP11-2 showed very low solubility, whereas AMP12-1, AMP16-1, and AMP16-2 exhibited slightly reduced solubility. In contrast, AMP13 precipitated rapidly after dissolution.

**Figure 3:**
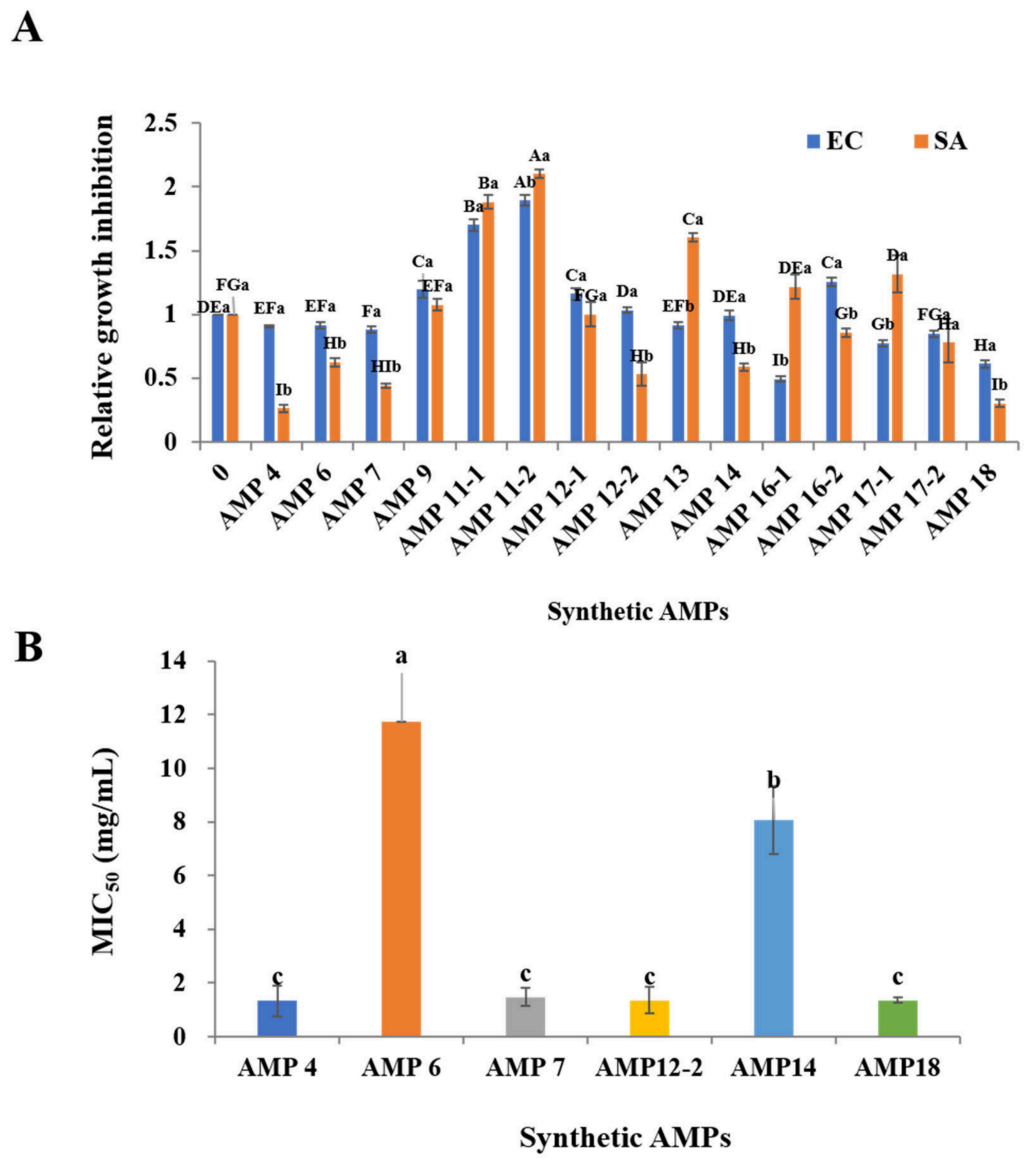
Antibacterial activity with the synthesized AMPs. (**A**) Antibacterial activities of the synthesized AMPs and (**B**) MIC_50_ of selected AMPs against *S. aureus*. Candidate AMPs were identified using CAMP_R4_ database (http://www.camp.bicnirrh.res.in/campHelp.php; accessed on 15 April 2024) and synthesized based on the predicted peptide sequences. Antibacterial activity was evaluated at a peptide concentration of 2.5 mg/mL. AMP12-2 indicates Winter flounder 1 homolog. Different lowercase and uppercase letters indicate significant differences among treatments (*p* < 0.05).

Antimicrobial activity was evaluated at a concentration of 2.5 mg/mL, the highest concentration previously used in studies on *Nibribacter* [13]. As shown in Figure 3A, the peptides exhibited very limited antimicrobial activity against *E. coli*. Among the tested peptides, only AMP16-1 and AMP18 demonstrated notable activity, with inhibition rates of 50.7% and 38.6%, respectively, relative to the control. In contrast, several peptides showed greater activities against *S. aureus*. AMP4, AMP7, and AMP18 exhibited relatively high antimicrobial activities of 73.4%, 55.7%, and 69.7%, respectively, compared with the control. Additionally, AMP6, AMP12-2, and AMP14 displayed moderate activities ranging from 37.5% to 46.8%. These findings suggest that the AMPs predicted from *L. plantarum* K9 possess greater antimicrobial activity against *S. aureus* than against *E. coli*, indicating a preference for Gram-positive bacteria.

Based on the observed antibacterial activities, the MIC_50_ values of the selected peptides against *S. aureus* were determined. AMP4 exhibited the lowest MIC_50_ value (1.53 mg/mL), indicating the strongest antibacterial activity. However, its MIC_50_ value was not significantly different from those of AMP7, AMP12-2, and AMP18. In contrast, AMP6 showed the highest MIC_50_ value among the tested peptides (11.9 mg/mL) for the MIC_50_ assay, while AMP14 exhibited an intermediate MIC_50_ value of 8.27 mg/mL.

### 2.5. Antimicrobial Activity of Cloned AMPs

**Figure 4:**
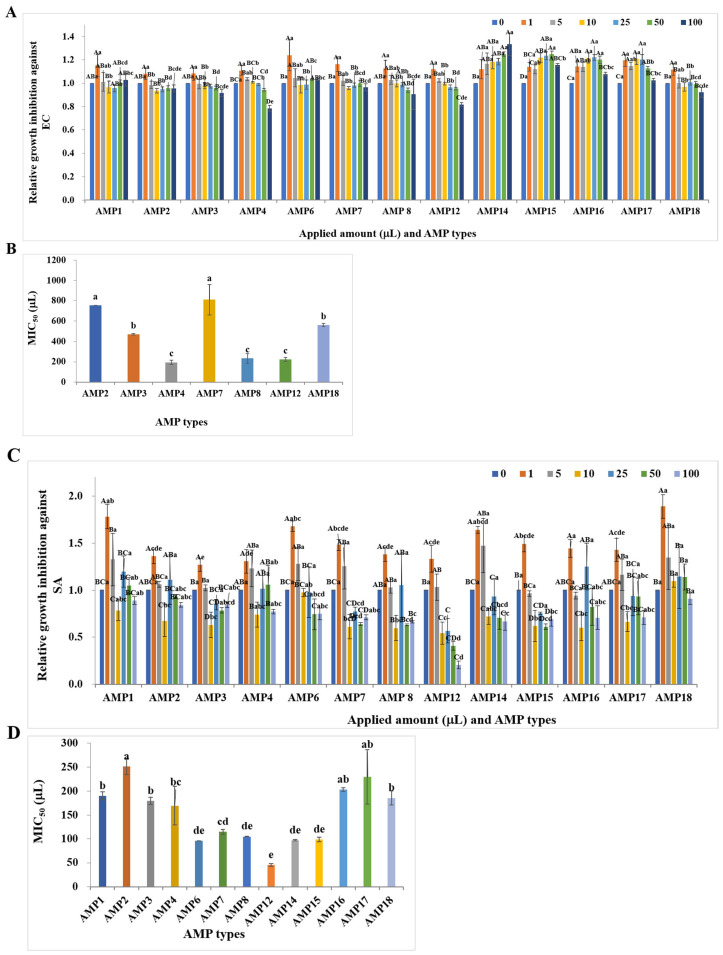
Antibacterial activity of cloned AMPs. Candidate AMP genes were expressed in *E. coli*, and cell-free culture broths were collected and used for antibacterial assays. Antibacterial activity (**A**) and MIC_50_ values (**B**) against *E. coli* (EC), as well as antibacterial activity (**C**) and MIC_50_ values (**D**) against *S. aureus* (SA), were determined. For the microtiter plate assay, *E. coli* and *S. aureus* cultures were adjusted to 10^6^ CFU/mL. Different lowercase and uppercase letters indicate statistically significant differences among treatments (*p* < 0.05).

To evaluate whether the candidate AMPs retain antimicrobial activity when expressed in *E. coli*, PCR primers were designed to amplify each AMP gene together with its upstream promoter and downstream terminator regions. The resulting PCR products were cloned into a T-vector and transformed into *E. coli*. Antimicrobial activity was subsequently evaluated using *E. coli* harboring the empty T-vector as a control. As shown in Figure 4A,B, antimicrobial activity against *E. coli* was generally low, consistent with the results obtained using the synthetic peptides. Among the tested clones, AMP4 exhibited the strongest antibacterial activity, with the lowest MIC_50_ value of 194.2 μL/mL, followed by AMP12 and AMP14 (Figure 4A,B). In contrast, AMP16, which displayed antibacterial activity in the synthetic peptide assay, showed no detectable activity when expressed in *E. coli*. AMP18 exhibited only weak activity, with an MIC_50_ value of 558.8 μL/mL. These findings suggest that the candidate AMPs exhibit limited antibacterial activity against *E. coli* when heterologously expressed in *E. coli*, indicating that either peptide expression, processing, or activity may be compromised in this host system.

Antimicrobial activity against *S. aureus* was substantially higher than that against *E. coli*, consistent with the results obtained using the synthetic peptides (Figure 4C,D). Among the tested clones, AMP12 exhibited the strongest antibacterial activity, with the lowest MIC_50_ value of 45.2 μL/mL. AMP6, AMP8, AMP14, and AMP15 also showed relatively high and comparable levels of activity. In the synthetic peptide assays, AMP4, AMP7, AMP12, and AMP18 demonstrated strong antibacterial activity against *S. aureus*. However, among the cloned AMP genes, only AMP7 and AMP12 consistently exhibited high antibacterial activity. These findings suggest that AMP7 and AMP12 are promising candidates for the development of Gram-positive bacteria-targeting antimicrobial peptides.

### 2.6. Characterization of the AMP12 Gene

**Figure 5:**
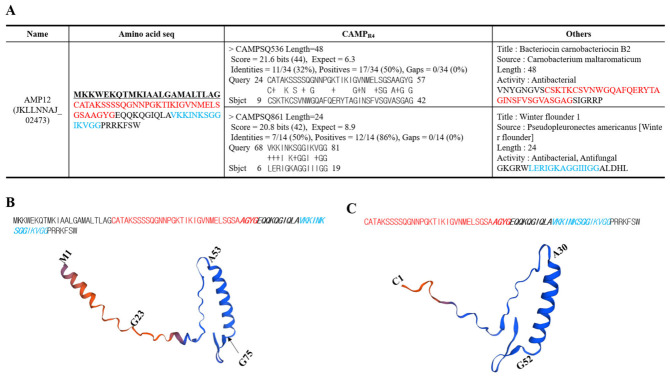
Properties of AMP12. (**A**) Prediction of AMP12 using CAMP_R4_. Red- and blue-colored letters indicate regions homologous to CAMPSQ536 (Bacteriocin carnobacteriocin B2) and CAMPSQ861 (Winter flounder 1), respectively. Signal peptide prediction was performed using SignalP 5.0 (https://services.healthtech.dtu.dk/service.php?SignalP-5.0; accessed on 15 April 2024). The signal peptide sequence, shown in bold and underlined letters, was predicted by both the Gram-positive and Gram-negative models in SignalP 5.0. (**B**) Predicted three-dimensional structure of the full-length AMP12 peptide. (**C**) Predicted three-dimensional structure of AMP12 without the signal peptide sequence. Bold and italic letters in (**B**,**C**) indicate the predicted α-helical region spanning residues A53 to G75.

The full-length peptide of AMP12 consists of 88 amino acids and contains two regions homologous to previously reported AMPs, carnobacteriocin B2 and Winter flounder 1 (Table 2 and Figure 5A). AMP12 was predicted to retain a signal sequence. The signal sequence of AMP12 predicted by SignalP 5.0 was identified as MKKWEKQTMKIAALGAMALTLAG, with a predicted cleavage site after G23, immediately preceding the region homologous to carnobacteriocin B2. The antimicrobial activities of the regions homologous to carnobacteriocin B2 and Winter flounder 1 were confirmed using the synthetic peptides AMP12-1 and AMP12-2, respectively, as well as the recombinant AMP12 protein (Figure 3 and Figure 4). However, further studies are needed to determine whether the antimicrobial activity of recombinant AMP12 is mediated by post-translational processing involving signal peptide removal and subsequent proteolytic cleavage, resulting in independently active peptide fragments, or by the intact full-length peptide acting as a single functional unit.

To predict the structural changes in AMP12 following signal sequence cleavage events, three-dimensional structural modeling was performed, and the results are presented in Figure 5B,C. The signal peptide region was predicted to form a distinct α-helix (Figure 5B). In addition, a second α-helical region spanning residues A53 to G75 was predicted, partially overlapping the sequences homologous to both carnobacteriocin B2 and winter flounder 1. Following cleavage of the signal peptide at G23, the α-helical structure corresponding to residues A53–G75 was predicted to remain intact (Figure 5C). Among AMP4, AMP7, AMP12, and AMP18, which demonstrated relatively high antimicrobial activity, the synthetic peptide of AMP12 exhibited antimicrobial activity comparable to those of the other candidates. Otherwise, the recombinant form of AMP12 showed similar antimicrobial activity against *E. coli*, while demonstrating enhanced antimicrobial activity against *S. aureus*. Specifically, when compared with those of other synthesized AMPs that displayed relatively high antimicrobial activity, the antimicrobial activity of recombinant AMP12 was greater than that of the individual synthetic peptides, AMP12-1 and AMP12-2.

## 3. Discussion

To isolate novel lactic acid bacteria, gut microbiota from *Protaetia brevitarsis* larvae were cultured on de Man, Rogosa, and Sharpe (MRS) medium, which is selective for lactic acid bacteria. Among the isolates obtained on MRS medium, ten strains exhibiting strong antimicrobial activity were selected for further analysis. Of these, strain LAB K9 displayed the most potent antimicrobial activity (Supplementary Material Figures S1 and S2). Based on 16S rDNA sequence analysis (Supplementary Material Figure S3), strain LAB K9 was identified as *Lactiplantibacillus plantarum* and was subsequently designated *Lactiplantibacillus plantarum* K9.

To evaluate the potential of this novel lactic acid bacterium, its antimicrobial activity was compared with that of *L. plantarum* NIBR97, a strain previously isolated in our laboratory [11]. *L. plantarum* K9 exhibited stronger antimicrobial activity than *L. plantarum* NIBR97 (Figure 1A and Table 1). Furthermore, unlike *L. plantarum* NIBR97, whose antimicrobial activity was unaffected by proteinase K treatment [11], the antimicrobial activity of *L. plantarum* K9 was reduced by 41.5–46.3% following proteinase K treatment (Figure 1B). The distinct responses observed between these closely related strains highlight substantial strain-specific differences in their antimicrobial compounds. Previous studies demonstrated that the antimicrobial activity of *Lactiplantibacillus taiwanensis* is also sensitive to proteinase K treatment [14], suggesting that *L. plantarum* K9 may produce proteinaceous antimicrobial substances like those produced by *L. taiwanensis*. In addition, the antimicrobial substances produced by *L. plantarum* K9 were found to exert their inhibitory effects through disruption of the target cell membrane, resulting in pore formation. This mode of action is consistent with previous observations reported for *L. plantarum* NIBR97 and *L. taiwanensis* (Figure 1C).

In contrast, *L. plantarum* K9, which was isolated from the gut of *P. brevitarsis* larvae, exhibited greater acid tolerance than *L. plantarum* NIBR97, a strain isolated from kimchi (Figure 1D). This observation may reflect the adaptive traits of lactic acid bacteria inhabiting the gastrointestinal environment. Thus, we suggest that *L. plantarum* K9 possesses physiological characteristics that enhance its survival under gut-associated conditions. Comparative genomic analysis of *L. plantarum* K9 (Figure 2A–D and Figures S50–S53) and *L. plantarum* NIBR97, whose genome was previously characterized [11], revealed a high degree of overall genomic similarity. Contigs 1–4 were highly conserved between the two strains; however, an additional contig (contig 5) was identified only in *L. plantarum* NIBR97 [11]. Although the genome sequence of *L. plantarum* K9 showed extensive homology with that of *L. plantarum* NIBR97, several observations suggest that further comparative studies are warranted. First, the two strains were isolated from markedly different ecological niches: *L. plantarum* NIBR97 was isolated from kimchi, a traditional Korean fermented food, whereas *L. plantarum* K9 was isolated from the gut of *P. brevitarsis* larvae. Second, the distinct responses of the two strains to proteinase K treatment indicate that the antimicrobial substances they produce are likely different in composition and mode of action. Finally, AMP12 was detected exclusively in *L. plantarum* K9 and was absent from *L. plantarum* NIBR97, suggesting that AMP12 may contribute to the strain-specific antimicrobial properties observed in *L. plantarum* K9.

A total of 18 putative AMPs were predicted in *L. plantarum* K9 using the CAMP_R4_ platform (Figure 2E). In a previous study, 11 AMPs were predicted in *Nibribacter radioresistens* using the same CAMP_R4_ program, and signal peptides were identified in NB AMP3, NB AMP6, and NB AMP9 [13]. The predicted NB AMPs ranged from 9 to 30 amino acids in length, with theoretical isoelectric points (pI) ranging from 3.8 to 10.85. Furthermore, the genes neighboring the NB AMP loci were predominantly annotated as hypothetical proteins, a pattern like that observed in the present study. Among the AMPs identified in *L. plantarum* K9 (designated LP AMPs), signal peptides were predicted in LP AMP1, LP AMP2, LP AMP12, LP AMP16, and LP AMP17. The LP AMPs exhibited considerable diversity in size, ranging from 14 to 62 amino acids, with predicted pI values between 4.9 and 12.61. Like the NB AMPs, the genomic regions surrounding the LP AMP loci were enriched with genes annotated as hypothetical proteins.

Based on transcriptomic analysis of *L. plantarum* K9, the number of genes exhibiting more than a two-fold change in expression increased progressively as acidity increased, compared with the untreated condition (Table 2 and Table 3). The differentially expressed genes were primarily associated with transcriptional regulation, heat shock proteins (HSPs), and transporter functions, suggesting that these genes play important roles in the acid tolerance response. In contrast, transcriptomic analysis across different growth phases revealed relatively few genes with substantial changes in expression. Unlike the response to acid stress, only a limited number of genes depending on different growth phases exhibited expression changes greater than two-fold. Specifically, four genes were significantly upregulated during the stationary phase, including three genes encoding peptidoglycan-binding domain-containing proteins and one gene encoding an extracellular membrane anchor protein. No genes exhibited a greater than two-fold increase in expression during the exponential growth phase. The increased expression of these stationary-phase-associated proteins may be related to processes required for long-term survival under nutrient-limited conditions, including antimicrobial activity, host interaction, immune recognition, biofilm formation, and cell envelope maintenance [15,16]. These findings suggest that *L. plantarum* K9 employs distinct transcriptional strategies in response to acid stress and growth phase, with acid tolerance involving broad transcriptional reprogramming, whereas adaptation to the stationary phase appears to depend on the regulation of a limited set of specialized genes.

Among the LP AMPs, AMP1, AMP4, AMP11, AMP12, and AMP15 exhibited increased expression during the exponential growth phase, whereas the remaining LP AMP genes showed the opposite expression pattern (Table 3). Similarly, transcriptomic analysis of *N. radioresistens* revealed that NB AMP genes display diverse expression patterns depending on the growth stage [13]. These observations suggest that the LP AMP genes identified in the present study may be associated with a variety of physiological functions and regulatory mechanisms. In contrast, under acidic conditions, LP AMP18 exhibited markedly increased expression relative to the untreated control, whereas no detectable expression of LP AMP13 was observed (Table 4). This finding suggests that LP AMP13 may either represent a misannotated candidate AMP or a gene that is expressed only under specific environmental conditions not examined in the present study. Previous transcriptomic studies of *L. plantarum* VAL6 demonstrated that pH changes alter the expression of genes involved in exopolysaccharide biosynthesis and a variety of other cellular processes [17,18]. Consistent with these observations, the pH conditions applied in the present study appear to have induced broad changes in gene expression, including alterations in the transcriptional profiles of LP AMP genes and other functional gene groups. These results suggest that environmental pH is an important factor influencing the regulatory networks and physiological responses of *L. plantarum* K9.

The synthetic LP AMPs identified in this study exhibited relatively low antimicrobial activity against *S. aureus*, with MIC_50_ values ranging from 1.53 to 11.94 mg/mL (Figure 3). These values were substantially higher than those reported in previous studies, in which synthetic AMPs exhibited MIC_50_ values of 2.2–128 μg/mL against *E. coli* and 32–256 μg/mL against *S. aureus* [13,19,20]. In addition, based on the activity observed at a concentration of 2.5 mg/mL, the synthetic LP AMPs were estimated to possess even lower antimicrobial activity against *E. coli*. These findings suggest that the antimicrobial activities of the predicted LP AMP candidates are considerably lower than expected from sequence-based predictions alone. Among the recombinant NB AMP clones previously identified in *N. radioresistens*, NB AMP9, NB AMP10, and NB AMP11 exhibited relatively strong antimicrobial activity against *E. coli*, with MIC_50_ values of 153.9, 131.0, and 154.0 μL/mL, respectively [13]. These activities were only slightly greater than that observed for LP AMP4 (Figure 4). Against *S. aureus*, NB AMP2, NB AMP3, NB AMP4, NB AMP5, NB AMP6, NB AMP7, NB AMP10, and NB AMP11 exhibited antimicrobial activity, with NB AMP6 showing the strongest effect and an MIC_50_ value of 123.1 μL/mL [13]. In contrast, recombinant LP AMP12 exhibited a markedly stronger antimicrobial effect against *S. aureus*, with an MIC_50_ value of 45.2 μL/mL, representing approximately a 2.7-fold improvement over the most active NB AMP clone. Notably, while high antimicrobial activity in *N. radioresistens* was observed either in synthetic peptides or in a limited number of recombinant AMP clones, LP AMP12 exhibited activity both as a synthetic peptide derivative and as a recombinant protein. This characteristic distinguishes LP AMP12 from previously reported AMP candidates. Furthermore, AMP12 is encoded by a gene that was detected exclusively in *L. plantarum* K9 and was absent from *L. plantarum* NIBR97. In addition, AMP12 contains a predicted signal peptide and exhibits particularly strong antimicrobial activity against the Gram-positive bacterium *S. aureus*. Taken together, these findings suggest that AMP12 is a unique antimicrobial peptide candidate with potential applications as a selective antimicrobial agent targeting Gram-positive pathogens.

Three-dimensional structural modeling of the synthetic NB AMPs from *N. radioresistens* demonstrated that the peptides exhibiting relatively high antimicrobial activity predominantly retained α-helical structures [13]. As reported in previous studies, it is suggested that the α-helical conformation of AMP12 plays an important role in its antimicrobial activity. In addition, EcDBS1R6 has been reported to be secreted following cleavage of its signal peptide by signal peptidase and subsequently exerts its antimicrobial activity through interactions with the bacterial membrane [21]. Because LP AMP12 also contains a predicted signal peptide, it may undergo a similar maturation process and mode of action. Furthermore, the strong antimicrobial activity of LP AMP12 against *S. aureus*, together with its relatively limited activity against *E. coli*, suggests that LP AMP12 may exhibit preferential activity against Gram-positive bacteria. However, further studies are required to clarify its secretion mechanism, processing pathway, and target specificity.

## 4. Materials and Methods

### 4.1. Evaluation of the Antimicrobial Efficacy of Cell-Free Extracts

Antimicrobial activity was evaluated using the Gram-negative bacterium *Escherichia coli* (EC, ATCC 25922) and the Gram-positive bacterium *Staphylococcus aureus* (SA, ATCC 6538) by either the disc diffusion method or a microtiter plate assay. For antimicrobial analysis, strains pre-cultured by overnight (O/N) were adjusted to a concentration of 10^6^ CFU/mL and used in the experiments. After adding the reaction mixtures to microtiter plates or spotting them onto discs, the plates were incubated at 37 °C overnight while observing the reaction outcomes. *Lactiplantibacillus plantarum* K9 was cultured in MRS medium at 37 °C for 24 h, followed by centrifugation at 12,000× *g* for 10 min. The supernatant was collected and filtered through a 0.2 μm filter to obtain a cell-free broth. For the antimicrobial assays, the cell-free broth was used at different concentrations, and the pre-cultured target strains were added after being adjusted to 10^6^ CFU/mL. The final reaction volume was adjusted to 200 μL with LB broth.

### 4.2. Acid Tolerance

*L. plantarum* K9 and NIBR97 were cultured to the exponential growth phase and used for the experiments. Each strain was centrifuged at 3500× *g* for 20 min, after which the supernatant was removed. The bacterial cells were washed twice with PBS buffer (pH 7.2) and then used for subsequent experiments. Fresh MRS broth was adjusted to pH 2.0, 2.5, 3.0, and 3.5 using 1 M HCl and used for the experiments. The cell pellets of each strain were resuspended in 10 mL of pH-adjusted MRS broth and incubated at 37 °C for 3 h. After incubation, the resulting suspensions were appropriately diluted with sterile distilled water, and viable cell counts were determined.

### 4.3. Scanning Electron Microscope (SEM)

*E. coli* was incubated with the cell-free broth from *L. plantarum* K9 culture for 24 h. The treated *E. coli* was incubated with the cell-free broth from *L. plantarum* K9 culture. The treated *E. coli* cells were fixed with one volume of 2.5% glutaraldehyde (Sigma-Aldrich, St. Louis, MO, USA) for 24 h at 4 °C. Then, the samples were rinsed with sterile PBS buffer thrice and sequentially dehydrated with graded ethanol (30%, 50%, 70%, 80%, 90%, and 100% (*v*/*v*); 15 min incubation for each concentration). Finally, the samples were dried at room temperature and sputter-coated with gold for SEM. Cells were fixed with one volume of 2.5% glutaraldehyde (Sigma-Aldrich, St. Louis, MO, USA) for 24 h at 4 °C. Then, the samples were rinsed with sterile PBS buffer thrice and sequentially dehydrated with graded ethanol (30%, 50%, 70%, 80%, 90%, and 100% (*v*/*v*); 15 min incubation for each concentration). Finally, the samples were dried at room temperature and sputter-coated with gold for SEM.

### 4.4. Genome Analysis of L. plantarum K9

Genome analysis of *L. plantarum* K9 was performed using a genome preparation kit (Wizard Genomic DNA Purification Kit, Promega Corporation, Madison, WI, USA). A total of 5 μg for each sample was used as input into library preparation. The SMRTbell library was constructed with SMRTbell™ Template Prep Kit 1.0 (PN 100-259-100) following manufacture’s instructions (Pacific Biosciences of California, Inc., Menlo Park, CA, USA). The small fragments lower than 20 kb of SMRTbell template were removed using Blue Pippin Size selection system for large-insert library. The constructed library was validated by Agilent 2100 Bioanalyzer (Agilent Technologies, Santa Clara, CA, USA). After a sequencing primer is annealed to the SMRTbell template, DNA polymerase is bound to the complex using DNA/Polymerase Binding kit P6 (Pacific Biosciences, Menlo Park, CA, USA). This polymerase-SMRTbell-adaptor complex is then loaded into SMRT cells. The SMRTbell library was sequenced using 1 SMRT cells (Pacific Biosciences) using C4 chemistry (DNA sequencing Reagent 4.0) and 240 min movies were captured for each SMRT cell using the PacBio RS II (Pacific Biosciences) sequencing platform [22].

*L. plantarum* K9 was prepared by P6-C4 chemistry and sequenced using 1 SMRT cell with MagBead OneCellPerWell v1 Protocol (Insert Sizes 20 kb, movie time 1 × 240 min). We produced 117,132 long reads and 913,773,737 base pairs after subreads filtering. *De novo* assembly was conducted using the hierarchical genome assembly process (HGAP, Version 2.3) workflow, including consensus polishing with Quiver [23]. As the estimated genome size was 3,244,413 bp and average coverage was 103X, we performed error correction based on the longest about 30X (487,589,278 bp) seed bases with rest shorter reads and then assembled with error corrected reads. As a result of HGAP process, we got the results 3,037,359 bp N50 contig and 4,382,491 bp total contig lengths by polish process. Finally, since bacterial genomes and plasmids are typically circular, we checked the forms for each of contigs using MUMmer 3.5 and trimmed one of the self-similar ends for manual genome closure [24].

Putative gene coding sequences (CDSs) from the assembled contigs were identified using Glimmer v3.02 [25] and open reading frames (ORFs) were obtained. These ORFs were searched using Blastall alignment (http://www.ncbi.nlm.nih.gov/books/NBK1762/; accessed on 12 May 2020) against the NCBI Non-redundant protein database (nr) for all species. GO annotation was assigned to each of ORFs by Blast2GO software analyzing the best hits of the BLAST results [https://www.blast2go.com/; accessed on 12 May 2020]. Additionally, ribosomal RNAs and transfer RNAs were predicted using RNAmmer 1.2 and tRNAscan-SE 1.4 [26,27].

### 4.5. Bioinformatic Analysis for Identification of AMPs from the L. plantarum K9 Genome

Peptides with a similarity (identities/positives) of more than 50% to existing AMPs and possessing a signal sequence cleaved by an endopeptidase were selected as AMP candidates using CAMP_R4_ (http://www.camp.bicnirrh.res.in/campHelp.php) (accessed on 15 April 2024) and SignalP 5.0 (https://services.healthtech.dtu.dk/services/SignalP-5.0/; accessed on 15 April 2024), respectively. Their amino acid and nucleotide sequences were subsequently used for peptide synthesis and gene cloning, respectively.

### 4.6. Transcriptomic Analysis of L. plantarum K9

For transcriptomic analysis, *L. plantarum* K9 was cultured to the exponential growth phase (A_600_ = 0.5) and the stationary phase (A_600_ ≥ 1.5), after which RNA was isolated from each condition. On the other hand, to observe changes in mRNA expressions in response to pH variation, cultured *L. plantarum* K9 cells were exposed to pH-adjusted conditions of 2.5 and 3.0 and incubated at 37 °C for 3 h, followed by RNA isolation from each condition. Total RNA extraction was performed using the AccuPrep^®^ Bacterial RNA Extraction Kit (Bioneer, Daejeon, Republic of Korea). RNA purity was measured using 1 μL of total RNA extract with a NanoDrop 1000 spectrophotometer (Thermo Fisher Scientific, Waltham, MA, USA). The quality of total RNA was verified by confirming the RNA Integrity Number (RIN) values measured using an Agilent Technologies 2100 Bioanalyzer (Agilent Technologies, Santa Clara, CA, USA). For total RNA sequencing library preparation, the Nugen Universal Prokaryotic RNA-seq Kit (Part Number 0363-32) was used, and all procedures were performed according to the manufacturer’s instructions. A total of 300 ng of total RNA was used to synthesize first- and second-strand cDNA using selective primers. After applying parameters specific to the Ovation protocol, the cDNA was fragmented to an average size of 200 bp by sonication using a Covaris S220 system in microtubes. All subsequent steps, including cDNA purification, end repair, adaptor ligation, first-strand selection, and first-strand purification, were performed according to the manufacturer’s official protocol. During Strand Selection II, rRNA was removed in a bacterial species–specific manner using the AnyDeplete technique with AnyDeplete probes (Tecan Asia Pte Ltd., Singapore). The libraries were amplified by PCR, and the quantity and quality of the amplified products were assessed by capillary electrophoresis (Agilent Technologies, Santa Clara, CA, USA). Following qPCR performed with SYBR Green PCR Master Mix (Applied Biosystems), equimolar amounts of index-tagged libraries were combined into a single pool. RNA sequencing was carried out using the Illumina NovaSeq 6000 system (Illumina, San Diego, CA, USA).

### 4.7. Expression Analysis Between Samples and Identification of DEGs

At first, readings for each sample were mapped to the reference genome by Tophat (v2.0.13). The aligned results were added to Cuffdiff (v2.2.0) to report differentially expressed genes. For library normalization and dispersion estimation, geometric and pooled (“blind” when each condition has single replicates, or “pooled” when multiple replicates are available) methods were applied.

Cuffdiff provides various output files, and using one of its outputs, “gene_exp.diff”, DEGs (Differentially Expressed Genes) were identified. To detect DEGs between sample1 as control and sample2 as case, two filtering processes were applied. First, using Cuffdiff status code, genes that only have “OK” status were extracted. Status code indicates whether each condition contains enough reads in a locus for a reliable calculation of expression level, and “OK” status means the test is successful to calculate gene expression level. For the second filtering, 2-fold change was calculated and genes belonging to the following range were selected.

Up-regulated:

*log*2[*case*] − *log*2[*control*] > = *log*2(2) = 1 log2[case] − log2[control] > = log2(2) = 1

Down-regulated:

*log*2[*case*] − *log*2[*control*] <= *log*2(1/2) = −1 log2[case] − log2[control] < = log2(1/2) = −1

For ontology analysis, genes after 2-fold change were picked (for mouse samples, genes with 2fold&pvalue < 0.05&FDR < 0.1 were selected for ontology analysis) and applied to DAVID as an input to get a comprehensive set of functional annotation. Disease, Gene ontology, pathway categories were selected, and Ease score was changed from 0.1 to 1 to include more output. Ease score is a conservative adjustment to the Fisher exact probability. It weights significance in favor of the association supported by more genes. DAVID then generated functional annotation chart which lists annotation terms and their associated genes under study.

### 4.8. L. plantarum K9 Amps Gene Cloning and Peptide Synthesis

General gene cloning was performed using the method of Sambrook et al. (2001) [28]. Candidate AMPs obtained through the analysis of the genome and transcriptome were designed and synthesized with primers for PCR amplification (Table 1; Genotech, Daejon, Republic of Korea). After amplifying each AMP gene using this primer set and PCR PreMix (AcuPower PCRMix, Bioneer, Daejeon, Republic of Korea), gel extraction was performed. Eluted DNA fragments were ligated to the T-easy vector (Promega, Madison, WI, USA) and transformed into *E. coli* DH5α (Invitrogen, Carlsbad, CA, USA) using the heat-shock method using calcium chloride. Cloned AMPs were identified using restriction enzyme cleavage and nucleotide sequencing (Applied Biosystems, Foster City, CA, USA).

Amino acid sequences in regions with homology to predicted AMPs were obtained, and these sequences were synthesized as artificial peptides (Table 2; Cosmogenetech, Seoul, Republic of Korea).

### 4.9. Evaluation of Antibacterial Activity of Cell-Free Supernatants and Synthetic Peptides

The cell-free supernatant of all *E. coli* DH5α strains containing cloned AMPs was collected after 24 h of culture and filtered through a 0.2 μm syringe (mixed cellulose esters (MCE), Merck, Darmstadt, Germany), and finally the cell-free supernatant was used for the evaluation of antibacterial activity. The cell-free supernatant from the *E. coli* DH5α, harboring only the plasmid vector without the LP-AMP genes, was confirmed as a negative control to have very little effect on antibacterial activities. The synthesized AMPs were suspended at a concentration of 10 mg/mL in distilled water and then used for antibacterial activity. The antibacterial activity against the Gram-negative bacterium, *E. coli* (EC; ATCC 10536), and the Gram-positive bacterium, *Staphylococcus aureus* (SA, ATCC 6538), was analyzed using the microtiter plate method and expressed as minimal inhibition concentration (MIC); the bacteria pre-cultured overnight (O/N) were cultured at 10^6^ CFU/mL with the cell-free supernatant or the synthetic peptides, the reaction mixtures were added to the microtiter plate, and finally the reactivity was observed by O/N culture at 37 °C.

### 4.10. Statistical Analysis

The collected data were analyzed using the PROC ANOVA procedure of the SAS program (ver. 9.2; SAS Institute Inc, Cary, NC, USA). Mean values that differed at the level of 5% significance were verified using Duncan’s multiple range test (DMRT).

## Figures and Tables

**Table 1:** MIC_90_ of the antibacterial activities of *L. plantarum* K9 and NIBR97 cell-free broths against *E. coli* and *S. aureus.*

K9 (μL Cell Free Broth/mL)		NIBR97 (μL Cell Free Broth/mL)	
*E. coli*	*S. aureus*	*E. coli*	*S. aureus*
21.80 ± 0.70	20.98 ± 0.02	26.38 ± 0.27	22.01 ± 0.19

**Table 2:** Results of the Transcriptomic Analysis of *L. plantarum* K9.

Experimental Conditions	Comparison	Up-Regulated Genes	Down-Regulated Genes	Total DEGs
pH dependence	pH 2.5/NT *	78	43	121
	pH 2.5/pH 3.0	0	0	0
	pH 3.0/NT	71	36	107
Growth dependence	stationary/exponential	4	7	11

* NT; nontreated condition (pH 3.5); Up- and downregulated values; number of genes.

**Table 3:** Growth Phase-Dependent Expression of Predicted AMP Genes.

AMP Name	Test ID	Exponential Phase	Stationary Phase	Fold Change (S/E ^1^)
PLK9_AMP1	JKLLNNAJ_00318	85.66	54.73	−1.57
PLK9_AMP2	JKLLNNAJ_00345	171.39	280.54	1.64
PLK9_AMP3	JKLLNNAJ_00536	141.24	160.78	1.14
PLK9_AMP4	JKLLNNAJ_00572	88.73	62.71	−1.41
PLK9_AMP5	JKLLNNAJ_01374	359.41	617.26	1.72
PLK9_AMP6	JKLLNNAJ_01523	283.48	311.55	1.10
PLK9_AMP7	JKLLNNAJ_01715	102.78	174.42	1.70
PLK9_AMP8	JKLLNNAJ_01850	489.89	585.47	1.20
PLK9_AMP9	JKLLNNAJ_02064	42.00	119.79	2.85
PLK9_AMP10	JKLLNNAJ_02068	-	59.68	inf
PLK9_AMP11	JKLLNNAJ_02183	534.58	645.19	−1.21
PLK9_AMP12	JKLLNNAJ_02473	122.46	76.28	−1.61
PLK9_AMP13	JKLLNNAJ_02474	-	-	-
PLK9_AMP14	JKLLNNAJ_02712	74.91	91.74	1.22
PLK9_AMP15	JKLLNNAJ_02746	164.80	127.07	−1.30
PLK9_AMP16	JKLLNNAJ_02882	7733.32	9948.07	1.29
PLK9_AMP17	INGLFCCM_00010	2594.51	3583.99	1.38
PLK9_AMP18	MLOLCKOO_00006	360.34	825.04	2.29

^1^ S/E indicates the stationary-phase/exponential-phase ratio. -: not detected expression; inf: infinite fold change.

**Table 4:** pH-Dependent Expression of Predicted AMP Genes.

AMP Name	Test_id	*L. plantarum* K9			Absolute Fold Change		
		NT (pH 3.5)	pH 2.5	pH 3.0	pH 2.5/NT	pH 2.5/3.0	pH 3.0/NT
PLK9_AMP1	JKLLNNAJ_00318	3.28	6.78	4.86	2.07	1.39	1.48
PLK9_AMP2	JKLLNNAJ_00345	50.78	138.55	62.53	2.73	2.22	1.23
PLK9_AMP3	JKLLNNAJ_00536	326.33	111.00	104.74	−2.94	−1.06	−3.12
PLK9_AMP4	JKLLNNAJ_00572	6.76	39.72	33.44	5.87	1.19	4.94
PLK9_AMP5	JKLLNNAJ_01374	2061.07	1021.32	831.42	−2.02	−1.23	−2.48
PLK9_AMP6	JKLLNNAJ_01523	312.00	377.70	328.68	1.21	1.15	1.05
PLK9_AMP7	JKLLNNAJ_01715	35.84	29.55	44.42	1.21	1.50	1.24
PLK9_AMP8	JKLLNNAJ_01850	154.75	105.92	101.16	−1.46	−1.05	−1.53
PLK9_AMP9	JKLLNNAJ_02064	0.00	155.67	163.76	-	1.05	-
PLK9_AMP10	JKLLNNAJ_02068	0.00	0.00	131.01	-	-	-
PLK9_AMP11	JKLLNNAJ_02183	211.48	274.38	288.72	1.30	1.05	1.37
PLK9_AMP12	JKLLNNAJ_02473	2.93	16.87	2.45	5.76	6.87	1.19
PLK9_AMP13	JKLLNNAJ_02474	0.00	0.00	0.00	-	-	-
PLK9_AMP14	JKLLNNAJ_02712	13.96	47.22	35.87	3.38	1.32	2.57
PLK9_AMP15	JKLLNNAJ_02746	49.36	20.85	28.04	−2.37	−1.35	−1.76
PLK9_AMP16	JKLLNNAJ_02882	2844.14	3543.81	3260.80	1.25	1.09	1.15
PLK9_AMP17	INGLFCCM_00010	29,710.00	25,855.90	21,787.60	−1.15	−1.19	−1.36
PLK9_AMP18	MLOLCKOO_00006	316.25	20.39	78.45	−15.51	−3.85	−4.03

NT: non-treated; -: expression changes were not characterized.

**Table 5:** Synthetic oligopeptides used for this study.

AMPs Name	ORF Sequences	Homologous AMPs		Synthesized AMPs			
		Homolog Name	Identities/ Positives (%)	Amino Acid Seq	Amino Acid No	pI	MW
AMP1	***MIKLRQVLKKILIGLMVFVLVFTAFSSSVDTVSA***HRRGVTHTRKHKVRHSASGHAKKAHRAHRKAKRRVVHRKAPHRRKVAVRHK**KKTPKRHKKAHKKKAAKKHKKAHKKKAAKKHKKSHKKKAAKKHKKSHKKKAAKKHGKSHKKK**SSKKHGKSNKKKSSKKHGKSNKKKSSKKHGKSHKKKSSKKHGKSHKKKSSKKHGKSSKKKSSKKHGKSNKKKSSKKHGKSSKKKSSKKHGKSNKKKSSKKHTSKGSAVIATLSRKTGISKGIISLLTDLVGYNDIYTMITGKDAATGKKRSRLVGAAWTALNFVPVSKVAKLAKAAKVL**ATAKKAEKVAKNGGRIKRAARATKLAMKRAAEKLAKRKPAKKAAKKAAEKARKTKK**VKHAKNVRATGQAKHEATHRAGTQLSKAKAKLEREKAAKHAKNVRATGQAKHEATHRAGT	Oncorhyncin II	50/56	KKTPKRHKKAHKKKAAKKHKKAHKKKAAKKHKKSHKKKAAKKHKKSHKKKAAKKHGKSHKKK	62	11.9	7279.00
		cgMolluscidin	41/57	ATAKKAEKVAKNGGRIKRAARATKLAMKRAAEKLAKRKPAKKAAKKAAEKARKTKK	56	11.79	6056.40
AMP2	*MLNKTINIIKKYPVRSLLVVLIVVFAIYVISDPSII**SS*** **FNQGLSDGTAGR**	Plantaricin JK	57/64	SSFNQGLSDGTAGR	14	5.55	1396.44
AMP3	MAE**VILVIIGALVLGILGR**ITFKFWQYGRPQHLRQSVSANSADDSAVTLFIPGYAGNRFSFGGMLQRFTAGASPISH	Preprotemporin-1SKa	63/81	VILVIIGALVLGILGR	16	9.72	1619.11
AMP4	MP**YLNVTKGSWDK**VGSTFTVKDNAEAPFTSLAGKSVVNIAMMPVQQGPWVYNTKSLSKDDQKKIATEFTSKSFAQNKKIFSEPNAKTPMMFPKKSEKSKLVNVTDKWYAPTHKLVGY	CNAP2	64/82	YLNVTKGSWDK	11	8.5	1310.47
AMP5	**MGWIGVAILGLTIGIVANAVGAHGRRQTTINLIAGLFGALGG**QLSFGWFGPTVAGMTLLPVVSGAMILIVIGTVAAQALQPQ	Bombin H7	57/86	ILGLTIGIVANAVG	14	5.52	1310.60
		Garvicin ML	36/53	GLTIGIVANAVGAHGRRQTTINLIAGLFGALGG	33	12	3189.71
AMP6	**MSQKNDNDKSQADKPWDKTFEDDRDDSGNLSRTQKRKQDSSNSTLTTVLVVLILLLALAPIGY**FLVKKNSLNNPQQTEQVASSSSKKSS**ASVAASKSSTAASKKKAAASSKKA**AAKSSSLALASSKAASSKAASSTAASSEESASTDDSSASSSSSSSESSSGTKYVTVEAGQGVYRVATNAGISVDKLLELNGLSSDATISAGQRLRVR	CAMPSQ1089	50/58	ASVAASKSSTAASKKKAAASSKKA	24	10.7	2236.55
AMP7	MGLVYASVRILSNRRVQTLEYLADELMLTSLGIINHQQPVTATHDQPA**IKPQPVTSVVKAAVKRV**	Buf III analog	47/65	IKPQPVTSVVKAAVKRV	17	11.26	1820.25
AMP8	MNR**EHIDELAEQQAERRRNLYHGLRTDKTHFEVSKH**PFEIVYDYRQGFDLDKFVERYSSILNKYDYIVGDWGFEQLRLKGFFRDDMKDVQRSQTIGAVQDYLYEYCNFGCAYFIIKNERVIKPKRTSRSRKDDRRGKKNTNSRQSR**ARSNRNSSRSKQRRKSHSFTTKQK**AAPFTEKRRQPAKVTAGNGKQAQTTKQSSGKRHFTIRQK	Hemoglobin subunit alpha	33/52	EHIDELAEQQAERRRNLYHGLRTDKTHFEVSKH	33	6.46	4044.42
		Protamine y1	42/67	ARSNRNSSRSKQRRKSHSFTTKQK	24	12.61	2876.19
AMP9	MSFFNDFHVLPGTHTSSYTSSVSNDI**FGGFHSFILNLIPAIKSLFSK**	Ponericin-W-like 321	38/57	FGGFHSFILNLIPAIKSLFSK	21	10	2336.81
AMP10	MSFFNDFHVLPGTHTSSYTSSVSNDI**FGGFHSFILNLIPAIKSLFSK**	Ponericin-W-like 321	38/57	FGGFHSFILNLIPAIKSLFSK	21	10	2336.81
AMP11	MKTTWLASLLVTIFW**GAVLGLVVTYLGGAM**VEALTAT**PIVREPFKAMAVGIILAVMSGLLVT**THH	Hymenochirin-5B (AMP11-1)	40/68	PIVREPFKAMAVGIILAVMSGLLVT	25	9.18	2626.30
		Bombinin-H4 (AMP11-2)	60/73	GAVLGLVVTYLGGAM	15	5.52	1420.73
AMP12	***MKKWEKQTMKIAALGAMALTLAGCATAKSSSS*QGNNPGKTIKIGVNMELSGSAAGYG**EQQKQGIQLA**VKKINKSGGIKVGG**PRRKFSW	carnobacteriocin B2 (AMP12-1)	32/50	CATAKSSSSQGNNPGKTIKIGVNMELSGSAAGYG	34	9.11	3286.64
		Winter flounder 1 (AMP12-2)	50/86	VKKINKSGGIKVGG	14	10.48	1384.69
AMP13	MKIQ**SNLTNLLTTLAVQNLALLLMKK**DPVT	D-LAK140	45/64	SNLTNLLTTLAVQNLALLLMKK	22	10	2412.96
AMP14	MDNIGYLLMQFSKQLRYQLNQRLIANGLTIQQWAVMQQISLWVERTAQQPTANQLCHVLDMDRPTMSGILRRLAAKRLVEQEVNPADQRAKLLRLTQVGIQEL**QAGQRISDQVVATGLQKLTAAEKQSL**RQLLKKLGSN	Cecropin-D	35/62	QAGQRISDQVVATGLQKLTAAEKQSL	26	8.59	2741.10
AMP15	MSDTAVMLKTTLLAGAILLENGAEIKRV**EDTMQRIVTNAGYPDAQVFVLLTGITVSLPENATSEVRAIHNRGM**DLEKVDQVNSLSRQFANHDIDLTEFAAALERVNQAVPTFPFSWLCLAAVVVSVPLMVAFTGRTTPADLITCGLAGLAGFAAFYWINRLGSIRFLSEFMGAFIIGLITLLGFYLTNGHYHPDAIIIGAVMPLVPGVAIT**NAVRDTMTGNLLSGPARAVEAVLSACAIGVGI**ALTSFFY	CAMPSQ1461	31/63	NAVRDTMTGNLLSGPARAVEAVLSACAIGVG	31	6.06	3014.47
		Carnocyclin A	30/53	EDTMQRIVTNAGYPDAQVFVLLTGITVSLPENATSEVRAIHNRGM	45	4.9	4916.56
AMP16	***MHWLWVLIIGAIIGAIAG*AITNKGKSMGWISNIIAGLVGSAI**GEALLGSWGPQLAGMAIVPSIIGGVIVVAITSFVLTRMD	GrammistinGsC (AMP16-1)	43/67	NKGKSMGWISNIIAGLVGSAI	21	10	2116.51
		Alyteserin-2b (AMP16-2)	44/81	IIGAIIGAIAGAITNK	16	8.75	1495.83
AMP17	***MHWLWVLIIGAIIGAIAG*AITSKGKSMGWIANIVAGLVGSSIGEAILGSWGPQLAGMA**IIPSIIGAVIVVAVVSFFLSRSTR	GrammistinGsC (AMP17-1)	50/72	KSMGWIANIVAGLVGSSI	18	8.75	1803.15
		Caerulein precursor-related fragment Ea	35/62	IGAIIGAIAGAITSKGKSMGWIANIV	26	10	2513.04
		Bacteriocin As-48	33/63	IIGAIAGAITSKGKSMGWIANIVA	24	10	2342.83
		Sln2-3	31/51	GKSMGWIANIVAGLVGSSIGEAILGSWGPQLAGMA	35	6	3399.97
		Grammistin Pp (AMP17-2)	56/81	KSMGWIANIVAGLVGS	16	8.75	1602.91
LP_AMP18	MSELKRYLIVAGINGAGKSTLYRARPELFTHSKRLNADEILQKMGGDWRKD**RDNFRAMREEIKQF**QRL	Latarcin 4a	43/86	RDNFRAMREEIKQF	14	8.74	1840.09

Bold letters indicate the predicted AMP sequences, whereas italicized and underlined sequences in AMP1, AMP2, AMP12, AMP16, and AMP17 represent the predicted signal sequences. The putative AMP and signal peptide sequences were predicted using CAMP_R4_ (http://www.camp.bicnirrh.res.in/campHelp.php; accessed on 15 April 2024) and SignalP 5.0 (https://services.healthtech.dtu.dk/services/SignalP-5.0/; accessed on 15 April 2024), respectively.

**Table 6:** Oligonucleotides used for this study.

Forward		Reverse	
Primer Name	Nucleotide Seq (5′→3′)	Primer Name	Nucleotide Seq (5′→3′)
AMP1-F-NdeI	CATATGCAGTTCCAAGTGTAACCTC	AMP1-R-XbaI	TCTAGATTTTCCATCGCGAACTCTAAC
AMP2-F-NdeI	CATATGATAATTGCACTGAGAACGCTA	AMP2-R-XbaI	TCTAGACAATATATTGGGATGCATCG
AMP3-F-NdeI	CATATGTGGACGTTGAAGTTGGCGA	AMP3-R-XbaI	TCTAGAGTCTGAACGTGCTCATGCGC
AMP4-F-BspHI	TCATGACGCTTGACGAATGTCAGTG	AMP4-R-XbaI	TCTAGAGCACACCACAAGAGCAAAGA
AMP5-F-NdeI	CATATGAAATGCCGTAAGTTGTTGCC	AMP5-R-XbaI	TCTAGAATGATAGCACCTGCAAAAGC
AMP6-F-NdeI	CATATGTGCCCAGTACGTGACTGCC	AMP6-R-XbaI	TCTAGAACGAGAATTGCGGGCGCTCA
AMP7-F-NdeI	CATATGCCGCAAATCAGCATCGACA	AMP7-R-XbaI	TCTAGAGCACGGCAGACTTTAATGGC
AMP8-F-NdeI	CATATGACCATCACCTGCAAGGTCA	AMP8-R-XbaI	TCTAGACCTCACGTGGAAAACGGGAC
AMP9-F-NdeI	CATATGAAACTCATAATGTATATCCTC	AMP9-R-XbaI	TCTAGATTACCAGGTACACATACGTC
AMP10-F-NdeI	CATATGGACGTATGTGTACCTGGTA	AMP10-R-XbaI	TCTAGAAGCAACCAACCACTTGGTAC
AMP11-F-NdeI	CATATGATCACCTAATGTTGTTGCATTA	AMP11-R-XbaI	TCTAGAGGCAGTGGCTGGCACTATTG
AMP12-F-NdeI	CATATGAAGTTCTTAGTCACAAAGGTTG	AMP12-R-XbaI	TCTAGAGTTGACAACCCTTGCTGTTC
AMP13-F-NdeI	CATATGTGCTACGTTGTTGACCCCG	AMP13-R-XbaI	TCTAGAAACCGAAGCTAATCAAGACC
AMP14-F-NdeI	CATATGGATTCCCCGTAACGCCGCA	AMP14-R-XhoI	CTCGAGGAGTAAGACGACACCGATTG
AMP15-F-NdeI	CATATGGCGGCTAATACGGAATCGTA	AMP15-R-XbaI	TCTAGATAGCGACGCCAATCACGATA
AMP16-F-NdeI	CATATGACTCATACTAAAAGCCACTAAC	AMP16-R-XbaI	TCTAGAGCCCTTTTCACGTAAATTAC
AMP17-F-BspHI	TCATGAAATCCTCCCAAAGATATGAC	AMP17-R-XbaI	TCTAGATTCTTTAACGCCTGTAATTG
AMP18-F-NcoI	CCATGGATGGCTGAACTAAGCGAATG	AMP18-R-XbaI	TCTAGATTTAACAACTTCATATGGTACC

## Data Availability

The original contributions presented in this study are included in the article. Further inquiries can be directed to the corresponding authors.

## Supplementary Materials

The following supporting information can be downloaded at: https://www.mdpi.com/article/10.3390/ijms27156843/s1.
