# Supplementary material for: Antibacterial Activities of Predicted AMP in Lactiplantibacillus plantarum K9
Source: Int J Mol Sci. 2026 Jul 30;27(15):6843. doi: 10.3390/ijms27156843 (PMC13467433; doi:10.3390/ijms27156843)

Suppl. Fig. S1. Antimicrobial activity of gut lactic acid bacteria strains from *Protaetia brevitarsis* larvae

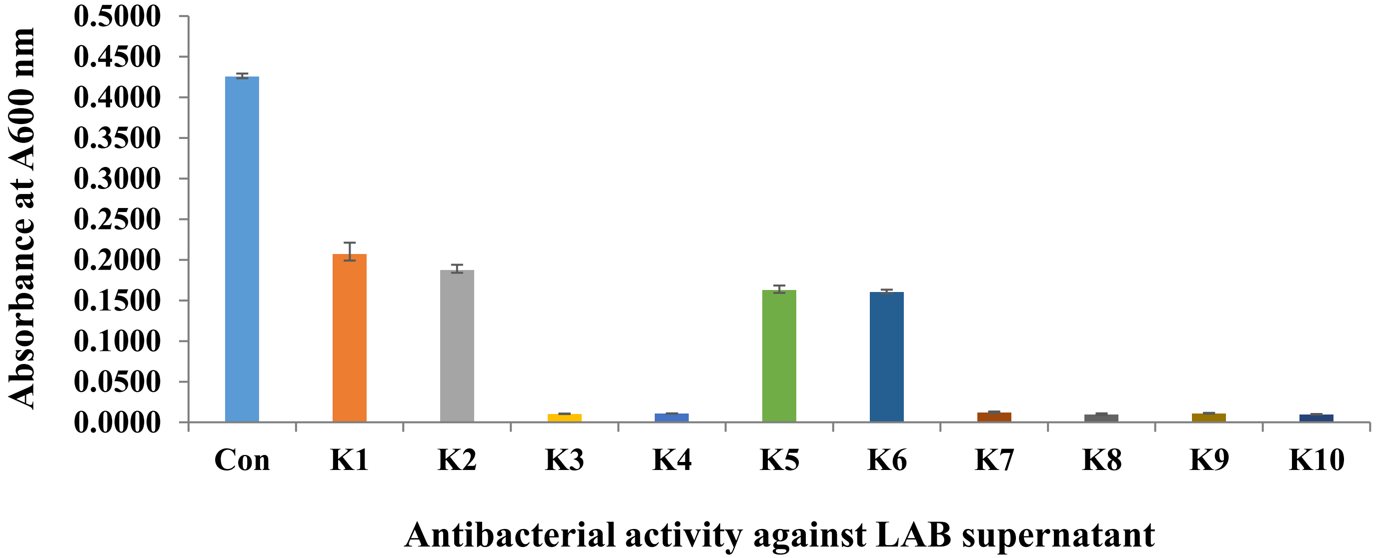

Sensitive bacteria: *E. coli* 10^6 CFU/mL
LABs supernatant vol: 50 ul/200 ul final volume

Suppl. Fig. S2. Antimicrobial activity of 1st selected gut lactic acid bacteria strains from *Protaetia brevitarsis* larvae

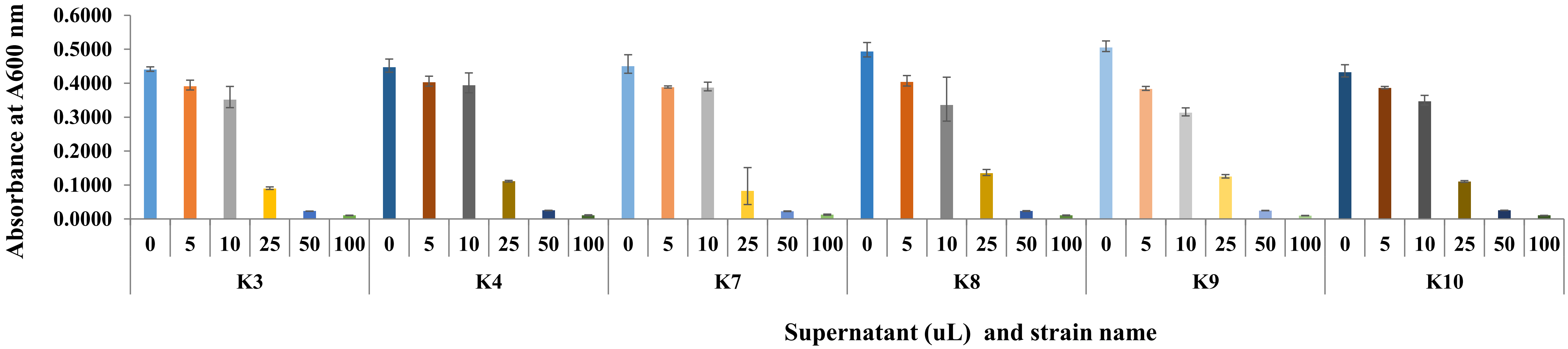

Sensitive bacteria: *E. coli* 10^6 CFU/mL
LABs supernatant vol: indicated amount

Suppl. Fig. S3. Comparison results of phylogenetic tree with L. plantarum K9 16S rDNA sequence

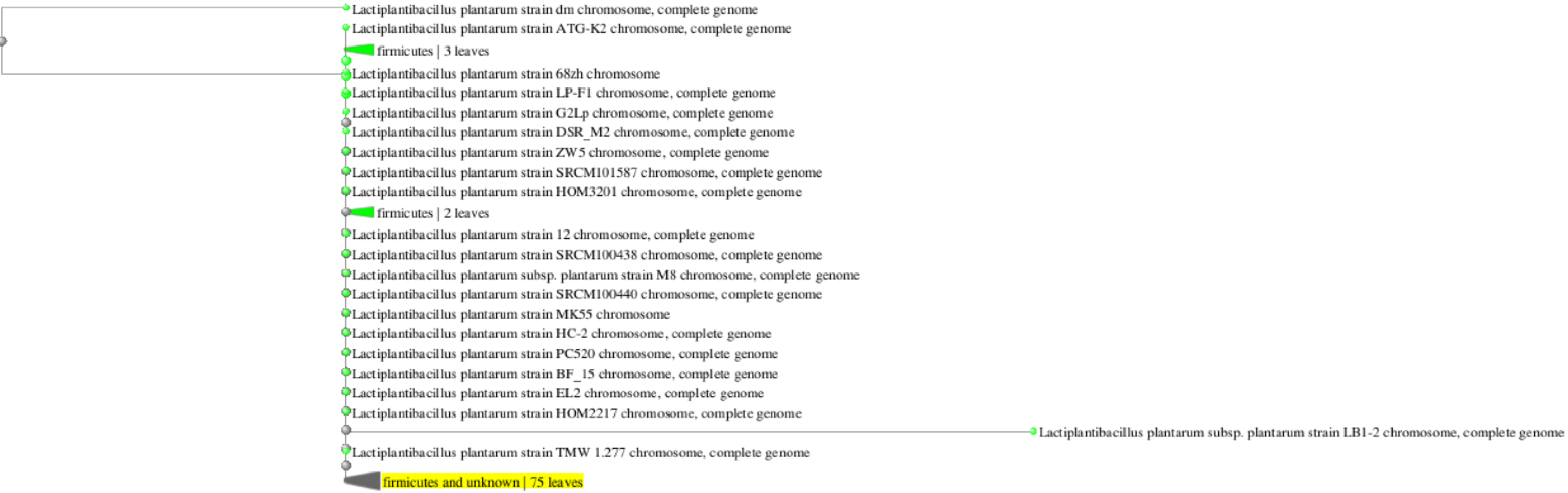

Suppl. Fig. S4. Antimicrobial activity depending on sensitive bacterial amount against *L. plantarum* K9 cell-free supernatant

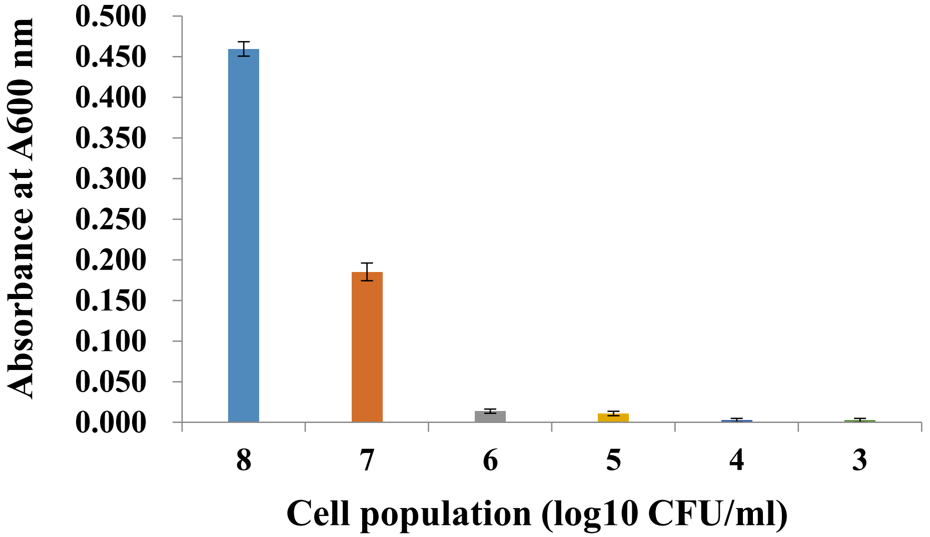

Sensitive bacteria: *E. coli* 10^6 CFU/mL
LABs supernatant vol: 50 ul/200 ul final volume

Suppl. Fig. S5. Antimicrobial activity depending on reaction temperature against *L. plantarum* K9 cell-free supernatant

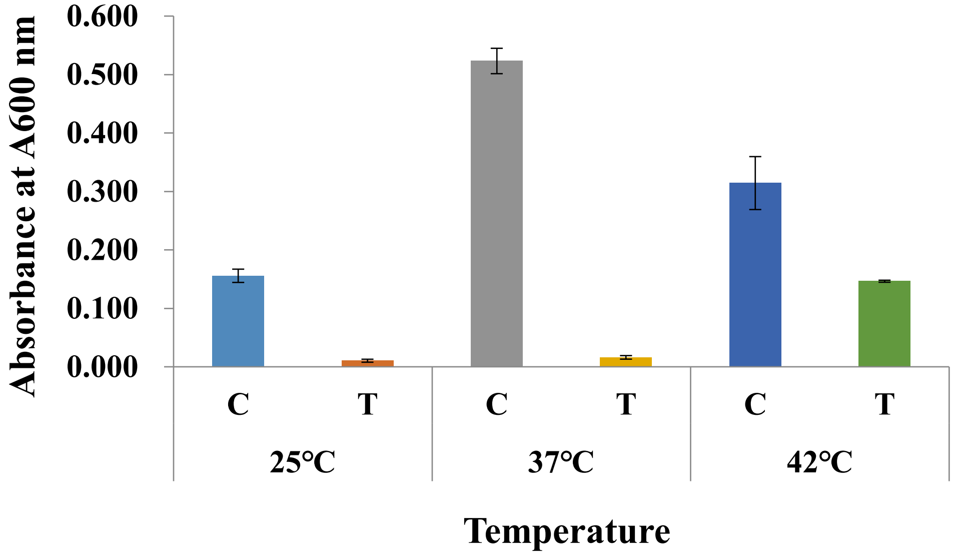

Sensitive bacteria: *E. coli* 10^6 CFU/mL
LABs supernatant vol: 50 ul/200 ul final volume

Suppl. Fig. S6. Evaluation for thermostability of *L. plantarum* K9 cell-free supernatant

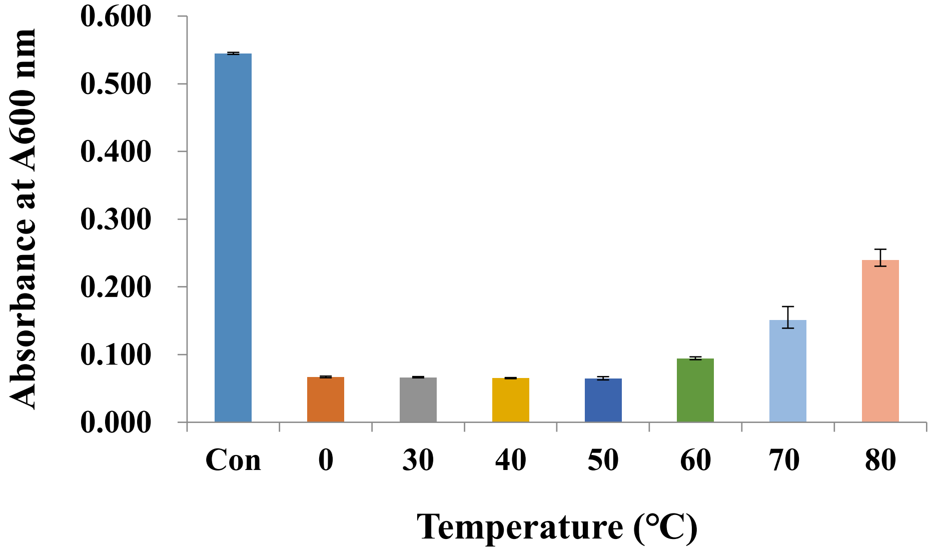

Sensitive bacteria: *E. coli* 10^6 CFU/mL
LABs supernatant vol: 50 ul/200 ul final volume

Suppl. Fig. S7. Evaluation for thermostability of *L. plantarum* K9 cell-free supernatant

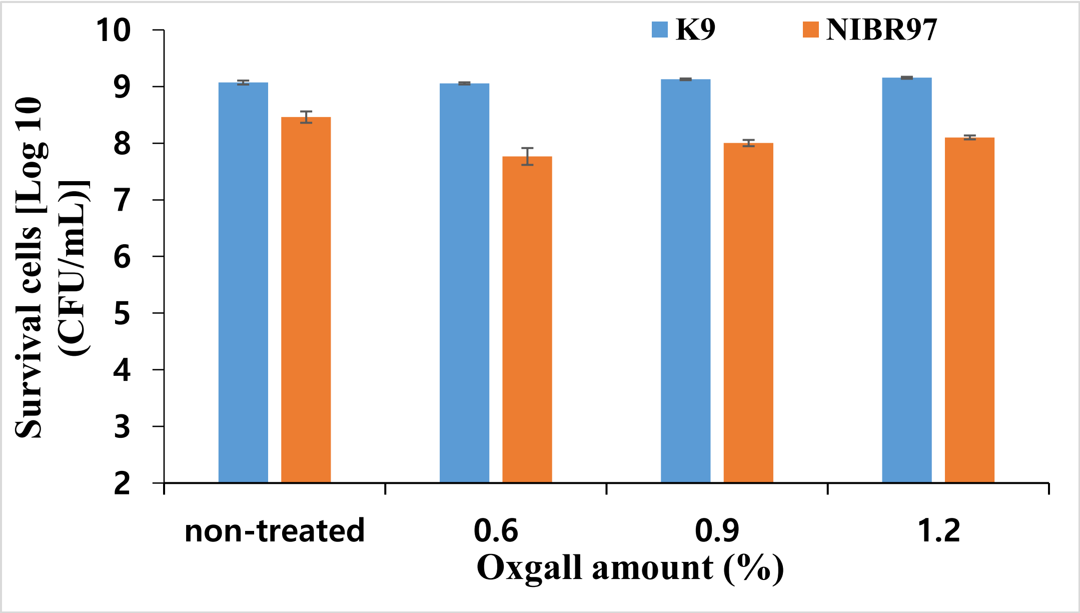

Sensitive bacteria: *E. coli* 10^6 CFU/mL
LABs supernatant vol: 50 ul/200 ul final volume

Suppl. Fig. S8. Putative AMPs

<table>
<tr><th>No</th><th colspan="2">Name</th><th>amino acid seq</th><th>amino acid No</th><th>campsign</th></tr>
<tr><td rowspan="2">1</td><td rowspan="2">>JKLLNNAJ_00318 hypothetical protein</td><td rowspan="2">AMP1</td><td rowspan="2">MIKLRQVLKKILIGLMVFVLVFTAFSSSVDTVSAHRRGVTHTRKHKV
RHSASGHAKKAHRAHRKAKRRVVHRKAPHRRKVAVRHKKKTPKRHKK
AHKKKAAKKHKKAHKKKAAKKHKKSHKKKAAKKHKKSHKKKAAKKHG
KSHKKKSSKKHGKSNKKKSSKKHGKSNKKKSSKKHGKSHKKKSSKKH
GKSHKKKSSKKHGKSSKKKSSKKHGKSNKKKSSKKHGKSSKKKSSKK
HGKSNKKKSSKKHTSKGSAVIATLSRKTGISKGIISLLTDLVGYNDI
YTMITGKDAATGKKRSRLVGAAWTALNFVPVSKVAKLAKAAKVLATA
KKAEKVAKNGGRIKRAARATKLAMKRAAEKLAKRKPAKKAAKKAAEK
ARKTKKVKHAKNVRATGQAKHEATHRAGTQLSKAKAKLEREKAAKHA
KNVRATGQAKHEATHRAGT</td><td rowspan="2">442</td><td>> CAMPSQ85 Length=69 Score = 45.4 bits (106), Expect = 5e-007
Identities = 32/64 (50%), Positives = 36/64 (56%), Gaps = 2/64 (3%)
Query 86 KKTPKRHKK-AHKKKAAKKHKKAHKKKAAKKHKKSHKKKAAKKHK-KSHKKKAAKKHGKS 143
KK+PK+ KK A KKAAK KK K AA K KKA K K K+ K KAAK +
Sbjct 6 KKSPKKAKKPATPKKAAKSPKKVKKPAAAAKKAAKSPKKATKAAKPKAAKPKAAKAKKAA 65

Query 144 HKKK 147
KKK
Sbjct 66 PKKK 69</td></tr>
<tr><td>> APD_1372|AP02232|cgMolluscidin Length=55 Score = 38.9 bits (89), Expect = 8e-0
05
Identities = 23/56 (41%), Positives = 32/56 (57%), Gaps = 2/56 (4%)
Query 327 ATAKKAEKVAKNGGRIKRAARATKLAMKRAAEKLAKRKPAKKAAKKAAEKARKTKK 382
ATAKK K A + K+A + + K+AA+K +K K+A KK A+K K KK
Sbjct 2 ATAKKGAKKADAPAKPKKATKPK--SPKKAAKKAGAKKGVKRAGKKGAKKTTKAKK 55</td></tr>
<tr><td>2</td><td>>JKLLNNAJ_00345 hypothetical protein</td><td>AMP2</td><td>MLNKTINIIKKYPVRSLLVVLIVVFAIYVISDPSIISSFNQGLSDGT
AGR</td><td>50</td><td>> CAMPSQ3099 Length=25 Score = 19.6 bits (39), Expect = 8.4
Identities = 8/14 (57%), Positives = 9/14 (64%), Gaps = 0/14 (0%)
Query 37 SSFNQGLSDGTAGR 50
SS +G DG AGR
Sbjct 8 SSLRKGFYDGEAGR 21</td></tr>
<tr><td>3</td><td>>JKLLNNAJ_00536 hypothetical protein</td><td>AMP3</td><td>MAEVILVIIGALVLGILGRITFKFWQYGRPQHLRQSVSANSADDSAV
TLFIPGYAGNRFSFGGMLQRFTAGASPISH</td><td>77</td><td>> CAMPSQ83 Length=19 Score = 22.3 bits (46), Expect = 1.9
Identities = 10/16 (63%), Positives = 13/16 (81%), Gaps = 0/16 (0%)
Query 4 VILVIIGALVLGILGR 19
VIL +IG L+ GILG+
Sbjct 4 VILPVIGKLLNGILGK 19</td></tr>
<tr><td>4</td><td>>JKLLNNAJ_00572 hypothetical protein</td><td>AMP4</td><td>MPYLNVTKGSWDKVGSTFTVKDNAEAPFTSLAGKSVVNIAMMPVQQG
PWVYNTKSLSKDDQKKIATEFTSKSFAQNKKIFSEPNAKTPMMFPKK
SEKSKLVNVTDKWYAPTHKLVGY</td><td>117</td><td>> DBAASP_1189|1314|CNAP2 Length=15 Score = 22.3 bits (46), Expect = 4.6
Identities = 7/11 (64%), Positives = 9/11 (82%), Gaps = 0/11 (0%)
Query 3 YLNVTKGSWDK 13
++NV KG WDK
Sbjct 2 HMNVQKGKWDK 12</td></tr>
<tr><td rowspan="2">5</td><td rowspan="2">>JKLLNNAJ_01374 hypothetical protein</td><td rowspan="2">AMP5</td><td rowspan="2">MGWIGVAILGLTIGIVANAVGAHGRRQTTINLIAGLFGALGGQLSFG
WFGPTVAGMTLLPVVSGAMILIVIGTVAAQALQPQ</td><td rowspan="2">82</td><td>> CAMPSQ659 Length=17 Score = 20.8 bits (42), Expect = 8.4
Identities = 8/14 (57%), Positives = 12/14 (86%), Gaps = 0/14 (0%)
Query 8 ILGLTIGIVANAVG 21
ILG +G+V+NA+G
Sbjct 1 ILGPILGLVSNALG 14</td></tr>
<tr><td>> CAMPSQ3403 Length=60 Score = 23.5 bits (49), Expect = 1.3
Identities = 13/36 (36%), Positives = 19/36 (53%), Gaps = 3/36 (8%)
Query 10 GLTIGI---VANAVGAHGRRQTTINLIAGLFGALGG 42
G+ G+ + NAV A T ++L +G F A GG
Sbjct 5 GMAAGVAKTIVNAVSAGMDIATALSLFSGAFTAAGG 40</td></tr>
<tr><td rowspan="2">6</td><td rowspan="2">>JKLLNNAJ_01523 hypothetical protein</td><td rowspan="2">AMP6</td><td rowspan="2">MSQKNDNDKSQADKPWDKTFEDDRDDSGNLSRTQKRKQDSSNSTLTT
VLVVLILLLALAPIGYFLVKKNSLNNPQQTEQVASSSSKKSSASVAA
SKSSTAASKKKAAASSKKAAAKSSSLALASSKAASSKAASSTAASSE
ESASTDDSSASSSSSSSESSSGTKYVTVEAGQGVYRVATNAGISVDK
LLELNGLSSDATISAGQRLRVR</td><td rowspan="2">210</td><td>> CAMPSQ85 Length=69 Score = 35.4 bits (80), Expect = 4e-004
Identities = 23/64 (36%), Positives = 34/64 (53%), Gaps = 3/64 (5%)
Query 81 ASSSSKKSSASVAASKSSTAASKKKAAASSKKAAAKSSSLALASSKAASSKAASSTAASS 140
A S KK+ K++ + K K A++ K AAKS A +KAA KAA AA +
Sbjct 5 AKKSPKKAKKPATPKKAAKSPKKVKKPAAAAKKAAKSPKKA---TKAAKPKAAKPKAAKA 61

Query 141 EESA 144
+++A
Sbjct 62 KKAA 65</td></tr>
<tr><td>> CAMPSQ1089 Length=30 Score = 23.1 bits (48), Expect = 4.8
Identities = 12/24 (50%), Positives = 14/24 (58%), Gaps = 0/24 (0%)
Query 90 ASVAASKSSTAASKKKAAASSKKA 113
A A+ + A KKKAAA KKA
Sbjct 4 APAPAAAAPAKAPKKKAAAKPKKA 27</td></tr>
</table>

No	Name		amino acid seq	amino acid No	campsign
7	>JKLLNNAJ_01715 hypothetical protein	AMP7	MGLVYASVRILSNRRVQTLEYLADELMLTSLGIINHQQPVTATHDQPAIKPQPVTSVVKAAVKRV	65	> DBAASP_4301|4651|Buf III analog Length=21 Score = 20.0 bits (40), Expect = 10.0<br>Identities = 8/17 (47%), Positives = 11/17 (65%), Gaps = 0/17 (0%)<br>Query 49 IKPQPVTSVVKAAVKRV 65<br>+K P+ VVK VK+V<br>Sbjct 3 VKQWPIGKVVKKVVKKV 19
8	>JKLLNNAJ_01850 hypothetical protein	AMP8	MNREHIDELAEQQAERRRNLYHGLRTDKTHFEVSKHPFEIVYDYRQGFDLDKFVERYSSILNKYDYIVGDWGFEQLRLKGFFRDDMKDVQRSQTIGAVQDYLYEYCNFGCAYFIIKNERVIKPKRTSRSRKDDRRGKKNTNSRQSRARSNRNSSRSKQRRKSHSFTTKQKAAPFTEKRRQPAKVTAGNGKQAQTTKQSSGKRHFTIRQK	209	> DBAASP_6067|6422|Hemoglobin subunit alpha Length=40 Score = 23.5 bits (49), Expect = 3.5<br>Identities = 11/33 (33%), Positives = 17/33 (52%), Gaps = 0/33 (0%)<br>Query 4 EHIDELAEQQAERRRNLYHGLRTDKTHFEVSKH 36<br>EH+D+L +E H LR D +F++ H<br>Sbjct 5 EHLDDLPGALSELSDLHAHKLRVDPVNFKLLSH 37<br><br>> Yadamp_1670|2500|Protamine y1 Length=31 Score = 22.3 bits (46), Expect = 8.5<br>Identities = 10/24 (42%), Positives = 16/24 (67%), Gaps = 0/24 (0%)<br>Query 147 ARSNRNSSRSKQRRKSHSFTTKQK 170<br>AR R+SSR +RR+ TT+++<br>Sbjct 1 ARRRRSSSRPIRRRRPRRRTTRRR 24
9	>JKLLNNAJ_02064 hypothetical protein	AMP9	MSFFNDFHVLPGTHTSSYTSSVSNDIFGGFHSFILNLIPAIKSLFSK	47	> CAMPSQ2603 Length=24 Score = 19.6 bits (39), Expect = 7.3<br>Identities = 8/21 (38%), Positives = 12/21 (57%), Gaps = 0/21 (0%)<br>Query 27 FGGFHSFILNLIPAIKSLFSK 47<br>FG S L+P++ LFS+<br>Sbjct 1 FGSLFSLGSKLLPSVFKLFSR 21
10	>JKLLNNAJ_02068 hypothetical protein	AMPs10	MSFFTDLHFLPGTHTSSYTSSVSNDIFGGFHSFILNLIPAIKSLFSK	47	> CAMPSQ2603 Length=24 Score = 19.6 bits (39), Expect = 7.3<br>Identities = 8/21 (38%), Positives = 12/21 (57%), Gaps = 0/21 (0%)<br>Query 27 FGGFHSFILNLIPAIKSLFSK 47<br>FG S L+P++ LFS+<br>Sbjct 1 FGSLFSLGSKLLPSVFKLFSR 21
11	>JKLLNNAJ_02183 hypothetical protein	AMPs11	MKTTWLASLLVTIFWGAVLGLVVTYLGGAMVEALTATPIVREPFKAMAVGIILAVMSGLLVTTHH	65	> CAMPSQ3668 Length=28 Score = 20.4 bits (41), Expect = 7.4<br>Identities = 10/25 (40%), Positives = 17/25 (68%), Gaps = 1/25 (4%)<br>Query 38 PIVREPFKAMAVGIILAVMSGLLVT 62<br>PIV++ K +A G +L+ ++G L T<br>Sbjct 5 PIVKDTLKKVAKG-VLSTIAGALST 28<br><br>> CAMPSQ649 Length=21 Score = 20.0 bits (40), Expect = 10.0<br>Identities = 9/15 (60%), Positives = 11/15 (73%), Gaps = 0/15 (0%)<br>Query 16 GAVLGLVVTYLGGAM 30<br>G VLGLV + LGG +<br>Sbjct 3 GPVLGLVGSALGGLL 17
12	>JKLLNNAJ_02473 hypothetical protein	AMP12	MKKWEKQTMKIAALGAMALTLAGCATAKSSSSQGNNPGKTIKIGVNMELSGSAAGYGEQQKQGIQLAVKKINKSGGIKVGGPRRKFSW	88	> CAMPSQ536 Length=48 Score = 21.6 bits (44), Expect = 6.3<br>Identities = 11/34 (32%), Positives = 17/34 (50%), Gaps = 0/34 (0%)<br>Query 24 CATAKSSSSQGNNPGKTIKIGVNMELSGSAAGYG 57<br>C+ K S + G + G+N +SG A+G G<br>Sbjct 9 CSKTKCSVNWGQAFQERYTAGINSFVSGVASGAG 42<br><br>> CAMPSQ861 Length=24 Score = 20.8 bits (42), Expect = 8.9<br>Identities = 7/14 (50%), Positives = 12/14 (86%), Gaps = 0/14 (0%)<br>Query 68 VKKINKSGGIKVGG 81<br>+++I K+GGI +GG<br>Sbjct 6 LERIGKAGGIIIGG 19

No	Name		amino acid seq	amino acid No	campsign
13	>JKLLNNAJ_02474 hypothetical protein	AMP13	MKIQSNLTNLLTTLAVQNLALLLMKKDPVT	30	> DBAASP_4173|4522|D-LAK140 Length=25 Score = 19.2 bits (38), Expect = 4.8<br>Identities = 10/22 (45%), Positives = 14/22 (64%), Gaps = 0/22 (0%)<br>Query 5 SNLTNLLTTLAVQNLALLLMKK 26<br>+ L L LA++ LALL +KK<br>Sbjct 4 AKLALLKWLLALKKLALLALKK 25
14	>JKLLNNAJ_02712 hypothetical protein	AMP14	MDNIGYLLMQFSKQLRYQLNQRLIANGLTIQQWAVMQQISLWVERTAQQPTANQLCHVLDMDRPTMSGILRRLAAKRLVEQEVNPADQRAKLLRLTQVGIQELQAGQRISDQVVATGLQKLTAAEKQSLRQLLKKLGSN	139	> CAMPSQ59 Length=36 Score = 23.1 bits (48), Expect = 2.7<br>Identities = 9/26 (35%), Positives = 16/26 (62%), Gaps = 0/26 (0%)<br>Query 104 QAGQRISDQVVATGLQKLTAAEKQSL 129<br>+AGQR+ D +++ G T A+ +L<br>Sbjct 9 RAGQRVRDAIISAGPAVATVAQATAL 34<br>> DBAASP_6046|6401| Length=48 Score = 22.7 bits (47), Expect = 4.1<br>Identities = 12/32 (38%), Positives = 19/32 (59%), Gaps = 0/32 (0%)<br>Query 74 AAKRLVEQEVNPADQRAKLLRLTQVGIQELQA 105<br>A K+ +E + + LL+LT GI++LQA<br>Sbjct 14 AIKKKIEAIEKEIEAQQHLLQLTVWGIKQLQA 45
15	>JKLLNNAJ_02746 hypothetical protein	AMP15	MSDTAVMLKTTLLAGAILLENGAEIKRVEDTMQRIVTNAGYPDAQVFVLLTGITVSLPENATSEVRAIHNRGMDLEKVDQVNSLSRQFANHDIDLTEFAAALERVNQAVPTFPFSWLCLAAVVVSVPLMVAFTGRTTPADLITCGLAGLAGFAAFYWINRLGSIRFLSEFMGAFIIGLITLLGFYLTNGHYHPDAIIIGAVMPLVPGVAITNAVRDTMTGNLLSGPARAVEAVLSACAIGVGIALTSFFY	250	> CAMPSQ1461 Length=44 Score = 23.5 bits (49), Expect = 4.3<br>Identities = 10/32 (31%), Positives = 20/32 (63%), Gaps = 0/32 (0%)<br>Query 212 NAVRDTMTGNLLSGPARAVEAVLSACAIGVGI 243<br>NA++DT+T + + A+ +++S GVG+<br>Sbjct 6 NAIKDTVTAAINNDGAKLGTSIVSIVENGVGL 37<br>> CAMPSQ813 Length=60 Score = 23.5 bits (49), Expect = 5.2<br>Identities = 14/47 (30%), Positives = 25/47 (53%), Gaps = 2/47 (4%)<br>Query 29 EDTMQRIVT--NAGYPDAQVFVLLTGITVSLPENATSEVRAIHNRGM 73<br>+ T +++V+ NAG + +L G+TV L T+ AI +G+<br>Sbjct 8 QGTAEKVVSLINAGLTVGSIISILGGVTVGLSGVFTAVKAAIAKQGI 54
16	>JKLLNNAJ_02882 hypothetical protein	AMP16	MHWLWVLIIGAIIGAIAGAITNKGKSMGWISNIIAGLVGSAIGEALLGSWGPQLAGMAIVPSIIGGVIVAITSFVLTRMD	81	> CAMPSQ1102 Length=26 Score = 20.8 bits (42), Expect = 7.8<br>Identities = 9/21 (43%), Positives = 14/21 (67%), Gaps = 0/21 (0%)<br>Query 22 NKGKSMGWISNIIAGLVGSAI 42<br>N K +G I+ + AGL+GS +<br>Sbjct 1 NWRKILGKIAKVAAGLLGSML 21<br>> CAMPSQ3254 Length=17 Score = 20.8 bits (42), Expect = 8.2<br>Identities = 7/16 (44%), Positives = 13/16 (81%), Gaps = 0/16 (0%)<br>Query 8 IIGAIIGAIAGAITNK 23<br>I+GAI+ ++G ++NK<br>Sbjct 1 ILGAILPLVSGLLSNK 16

No	Name		amino acid seq	amino acid No	campsign
17	>INGLFCCM_00010 hy<br>pothetical protein	AMP17	MHWLWVLIIGAIIGAIAGAITSKGKSMGWIANIVAGLV<br>GSSIGEAILGSWGPQLAGMAIIPSIIGAVIVVAVVSFF<br>LSRSTR	82	> CAMPSQ1102 Length=26 Score = 21.9 bits (45), Expect = 3.0<br>Identities = 9/18 (50%), Positives = 13/18 (72%), Gaps = 0/18 (0%)<br>Query 25 KSMGWIANIVAGLVGSSI 42<br>K +G IA + AGL+GS +<br>Sbjct 4 KILGKIAKVAAGLLGSML 21<br><br>> CAMPSQ2899 Length=31 Score = 21.9 bits (45), Expect = 3.1<br>Identities = 9/26 (35%), Positives = 16/26 (62%), Gaps = 0/26 (0%)<br>Query 9 IGAIIGAIAGAITSKGKSMGWIANIV 34<br>+G+I+G I GK++G +A+ V<br>Sbjct 2 LGSILGKILNVAGKVGKTIGKVADAV 27<br><br>> CAMPSQ1026 Length=30 Score = 21.6 bits (44), Expect = 4.2<br>Identities = 8/24 (33%), Positives = 15/24 (63%), Gaps = 0/24 (0%)<br>Query 12 IIGAIAGAITSKGKSMGWIANIVA 35<br>I A+AG + + ++ GW+ IV+<br>Sbjct 6 IPAAVAGTVLNVVEAGGWVTTIVS 29<br><br>> DBAASP_1407|1537|Sln2-3 Length=46 Score = 21.6 bits (44), Expect = 5.4<br>Identities = 11/35 (31%), Positives = 18/35 (51%), Gaps = 1/35 (3%)<br>Query 24 GKSMGWIANIVAGLVGSSIGEAILGSWGPQLAGMA 58<br>G W+ + AG+VG ++ AI G W G++<br>Sbjct 6 GSGNHWV-HCGAGIVGGALIGAIGGPWSAVAGGIS 39<br><br>> CAMPSQ1094 Length=25 Score = 21.2 bits (43), Expect = 5.8<br>Identities = 9/16 (56%), Positives = 13/16 (81%), Gaps = 0/16 (0%)<br>Query 25 KSMGWIANIVAGLVGS 40<br>K +G IA++ AGL+GS<br>Sbjct 4 KILGQIASVGAGLLGS 19
18	>MLOLCKOO_00006 hy<br>pothetical protein	AMP18	MSELKRYLIVAGINGAGKSTLYRARPELFTHSKRLNAD<br>EILQKMGGDWRKDRDNFRAMREEIKQFQRL	68	> CAMPSQ4328 Length=24 Score = 20.4 bits (41), Expect = 7.8<br>Identities = 6/14 (43%), Positives = 12/14 (86%), Gaps = 0/14 (0%)<br>Query 52 RDNFRAMREEIKQF 65<br>+D F++M E++KQ+<br>Sbjct 3 KDKFKSMGEKLKQY 16

Suppl. Fig. S9. Prediction of signal sequence for LPK9 AMP1

<table>
<tr><th>Name</th><th>amino acid seq</th><th>amino acid No</th><th>campsign</th><th>Others</th></tr>
<tr><td rowspan="2">>JKLLNNAJ_00318 hypothetical protein</td><td rowspan="2">MIKLRQVLKKILIGLMVFVLVFTAFSSSVDTVSAHRRGVT
HTRKHKVRHSASGHAKKAHRAHRKAKRRVVHRKAPHRRKV
AVRHKKKTPKRHKKAHKKKAAKKHKKAHKKKAAKKHKKSH
KKKAAKKHKKSHKKKAAKKHGKSHKKKSSKKHGKSNKKKS
SKKHGKSNKKKSSKKHGKSHKKKSSKKHGKSHKKKSSKKH
GKSSKKKSSKKHGKSNKKKSSKKHGKSSKKKSSKKHGKSN
KKKSSKKHTSKGSAVIATLSRKTGISKGIISLLTDLVGYN
DIYTMITGKDAATGKKRSRLVGAAWTALNFVPVSKVAKLA
KAAKVLATAKKAEKVAKNGGRIKRAARATKLAMKRAAEKL
AKRKPAKKAAKKAAEKARKTKKVKHAKNVRATGQAKHEAT
HRAGTQLSKAKAKLEREKAAKHAKNVRATGQAKHEATHRA
GT</td><td rowspan="2">442</td><td>> CAMPSQ85 Length=69
Score = 45.4 bits (106), Expect = 5e-007
Identities = 32/64 (50%), Positives = 36/64 (56%), Gaps = 2/64 (3%)
Query 86 KKTPKRHKK-AHKKKAAKKHKKAHKKKAAKKHKKSHKKKAAKKHK-KSHKKKAAKKHGKS 143
KK+PK+ KK A KKAAK KK K AA K KKA K K K+ K KAAK +
Sbjct 6 KKSPKKAKKPATPKKAAKSPKKVKKPAAAAKKAAKSPKKATKAAKPKAAKPKAAKAKKAA 65

Query 144 HKKK 147
KKK
Sbjct 66 PKKK 69</td><td>Oncorhyncin II, Source : Oncorhynchus mykiss [Rainbow trout, Antibacterial
KAVAAKKSPKKAKKPATPKKAAKSPKKVKKPAAAAKKAAKSPKKATKAAKPKAAKPKAAKAKKAAPKKK</td></tr>
<tr><td>> APD_1372|AP02232|cgMolluscidin Length=55
Score = 38.9 bits (89), Expect = 8e-005
Identities = 23/56 (41%), Positives = 32/56 (57%), Gaps = 2/56 (4%)
Query 327 ATAKKAEKVAKNGGRIKRAARATKLAMKRAAEKLAKRKPAKKAAKKAAEKARKTKK 382
ATAKK K A + K+A + + K+AA+K +K K+A KK A+K K KK
Sbjct 2 ATAKKGAKKADAPAKPKKATKPK--SPKKAAKKAGAKKGVKRAGKKGAKKTTKAKK 55</td><td>Anti-Gram+ & Gram -
AATAKKGAKKADAPAKPKKATKPKSPKKAAKKAGAKKGVKRAGKKGAKKTTKAKK</td></tr>
</table>

	Oncorhyncin II homolog	cgMolluscidin homolog
total hydrophobic ratio	16 %	39%
total net charge	+ 37.5	+21
Secondary structure	helix	helix

protein type	Signal peptide (Sec/SPI)	TAT signal peptide (Tat/SPI)	Lipoprotein signal peptide (Sec/SPII)	Other
**Likelihood**	0.6394	0.0274	0.2112	0.122

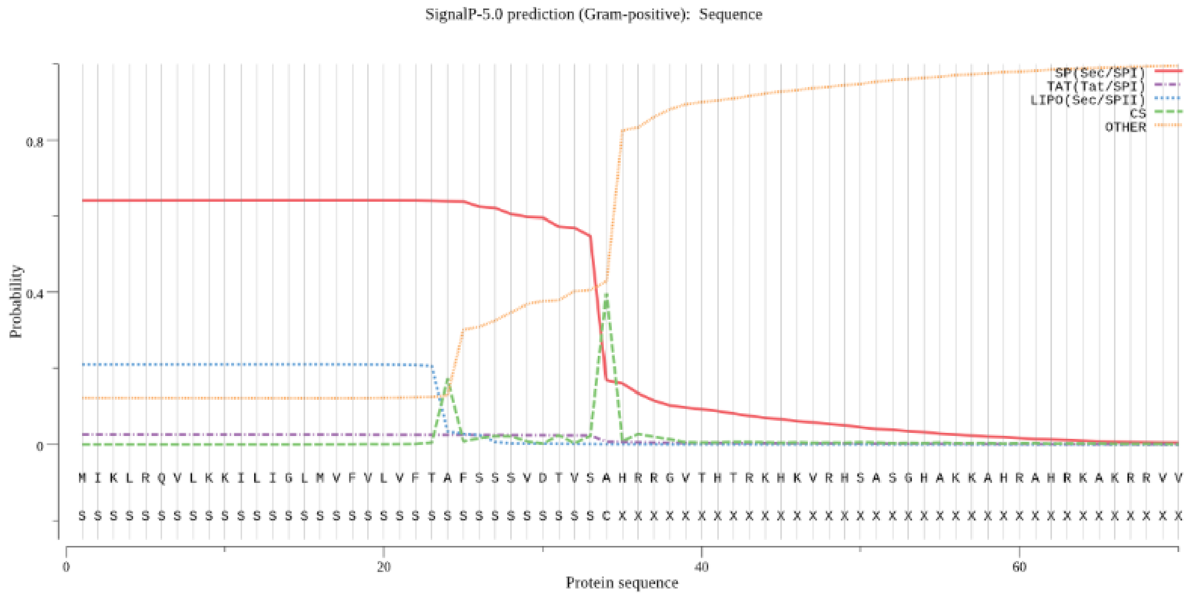

Suppl. Fig. S10. Arrangement of franking genes of LPK9 AMP1

contig 1

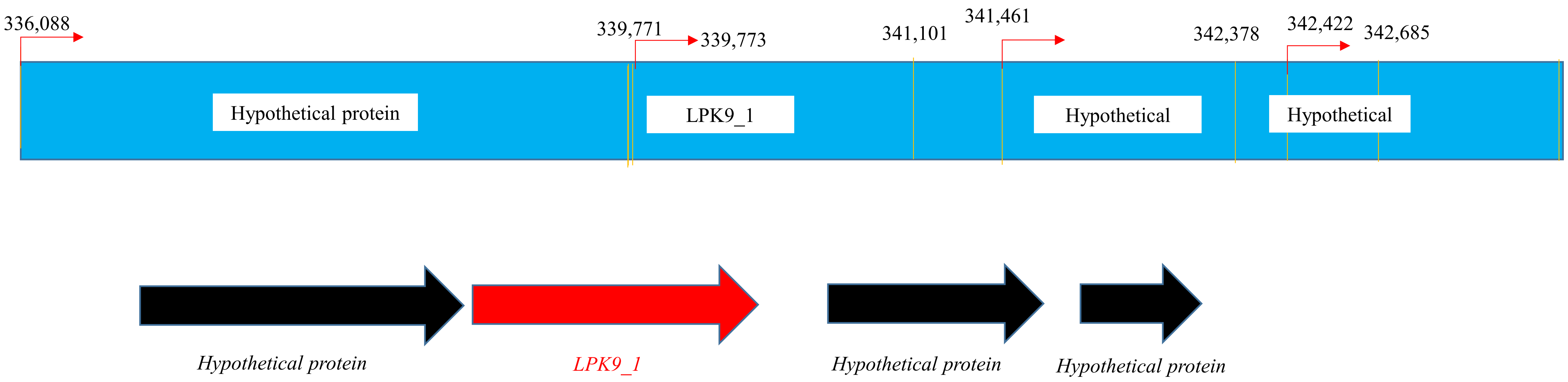

Suppl. Fig. S11. Prediction of signal sequence for LPK9 AMP2

Name	amino acid seq	amino acid No	campsign	Others
>JKLLNNAJ_00345 hypothetical protein	MLNKTINIIKKYPVRSLLVVLIVVFAIYVISDPSIISSFN QGLSDGTAGR	50	> CAMPSQ3099<br>Length=25<br>Score = 19.6 bits (39), Expect = 8.4<br>Identities = 8/14 (57%), Positives = 9/14 (64%), Gaps = 0/14 (0%)<br>Query 37 SSFNQGLSDGTAGR 50<br>SS +G DG AGR<br>Sbjct 8 SSLRKGFYDGEAGR 21	Title : Plantaricin JK<br>Source : Lactobacillus plantarum C1<br>Length : 25<br>Activity : Antimicrobial<br>Validated : Experimentally Validated<br>Sequence: GAWKNFWSSLRKGFYDGEAGRAIRR

total hydrophobic ratio (%)	21
total net charge	0
MW (Da)	1396.438
Secondary structure	helix

Protein type	Signal peptide (Sec/SPI)	TAT signal peptide (Tat/SPI)	Lipoprotein signal peptide (Sec/SPII)	Other
**Likelihood**	0.5216	0.0012	0.0129	0.4643

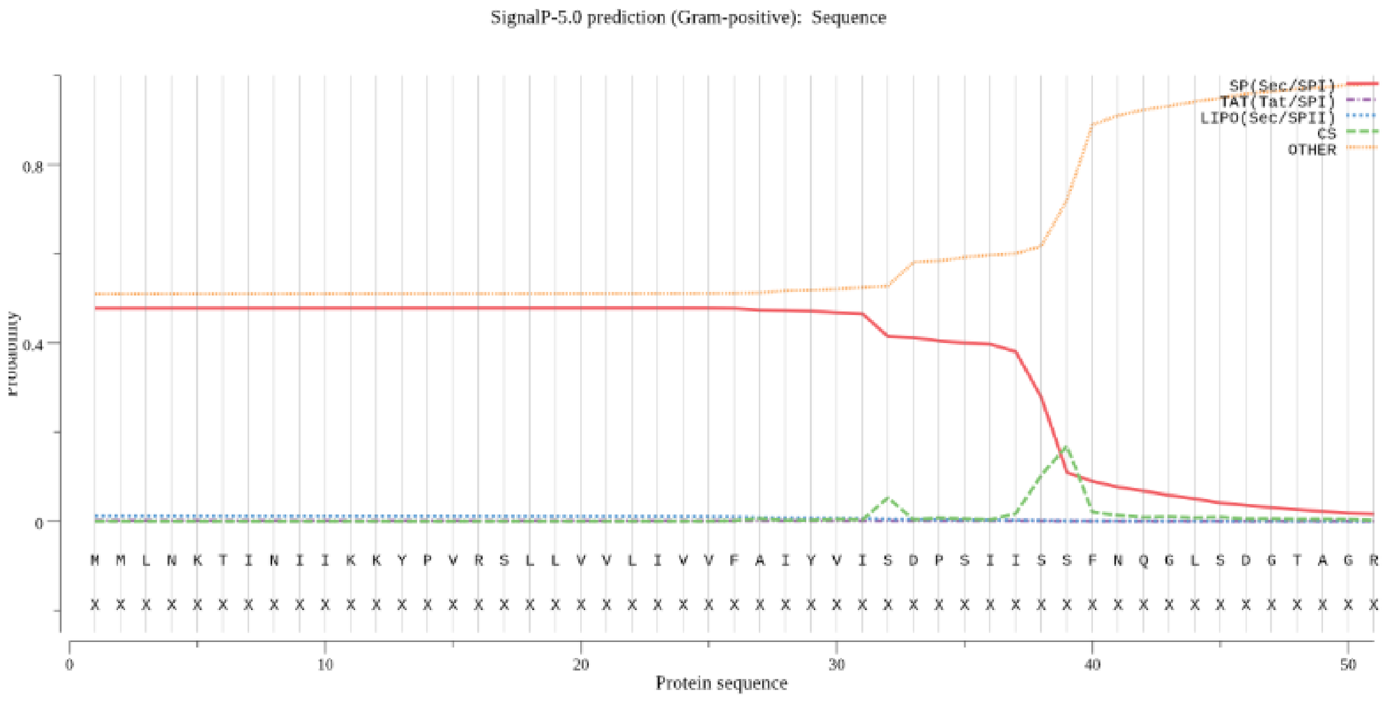

Supl. Fig. S12. Arrangement of franking genes of LPK9 AMP2

contig 1

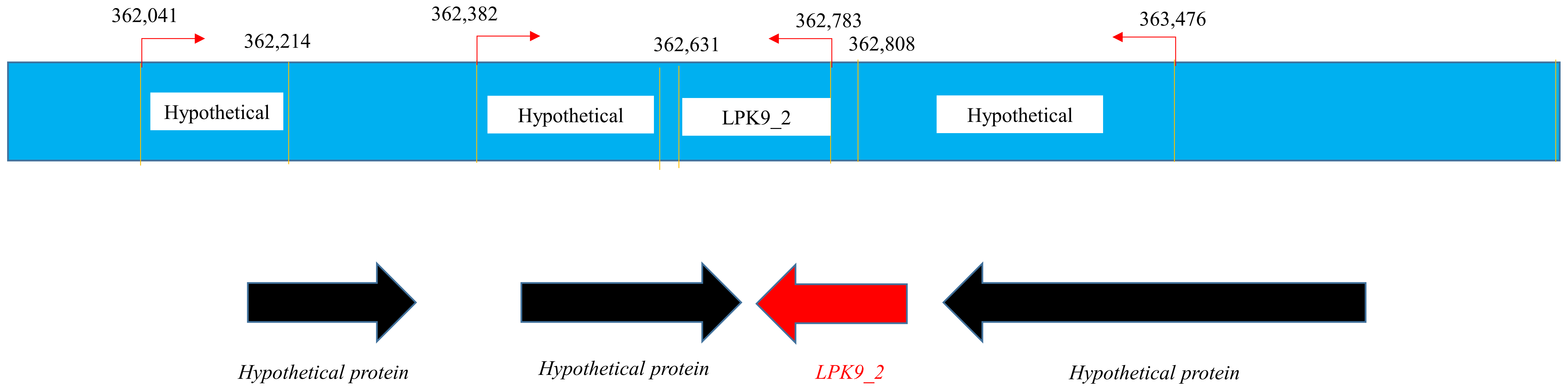

Suppl. Fig. S13. Prediction of signal sequence for LPK9 AMP3

Name	amino acid seq	amino acid No	campsign	Others
>JKLLNNAJ_00536 hypothetical protein	MAEVILVIIGALVLGILGRITFKFWQYGRPQHLRQSVSANSADDSAVTLFIPGYAGNRFSFGGMLQRFTAGASPISH	77	> CAMPSQ83<br>Length=19<br>Score = 22.3 bits (46), Expect = 1.9<br>Identities = 10/16 (63%), Positives = 13/16 (81%), Gaps = 0/16 (0%)<br>Query 4 VILVIIGALVLGILGR 19<br>VIL +IG L+ GILG+<br>Sbjct 4 VILPVIGKLLNGILGK 19	Preprotemporin-1SKa Source : Rana sakuraii [Japanese brown frog] Antibacterial, Antifungal<br>FLPVILPVIGKLLNGILGK<br>* NOT OVERLAPPING WITH NIBR97

total hydrophobic ratio (%)	75
total net charge	+1
MW (Da)	1619.117
Secondary structure	helix

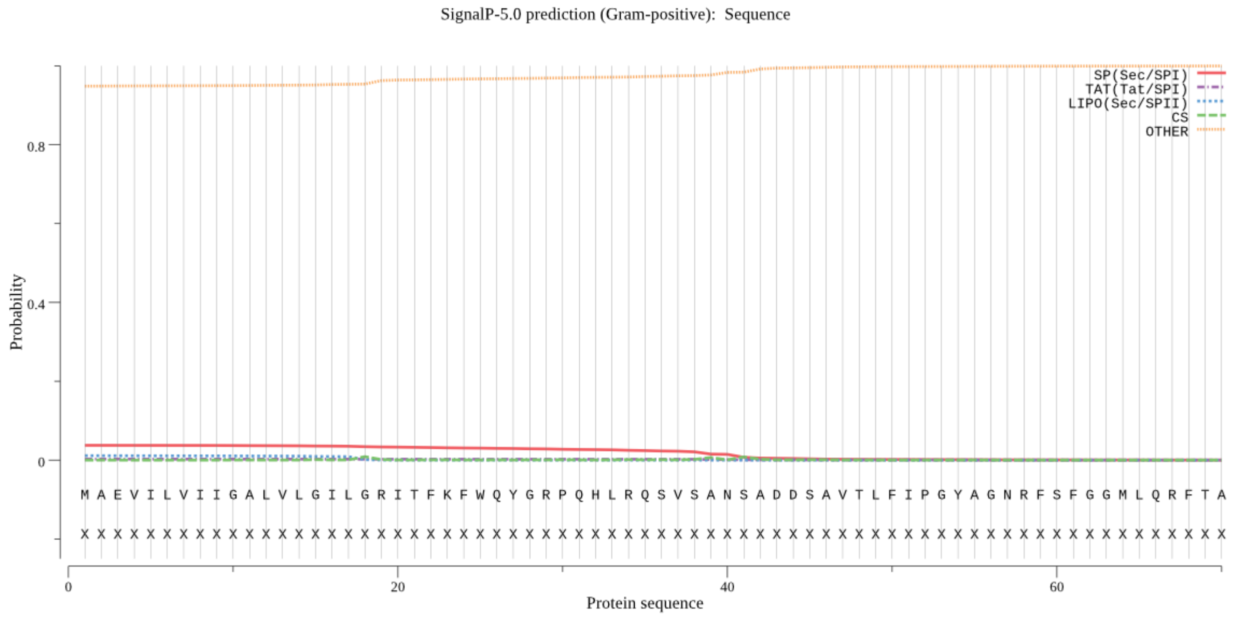

protein type	Signal peptide (Sec/SPI)	TAT signal peptide (Tat/SPI)	Lipoprotein signal peptide (Sec/SPII)	Other
**Likelihood**	0.0375	0.0029	0.0115	0.9481

Suppl. Fig. S14. Arrangement of franking genes of LPK9 AMP3

contig 1

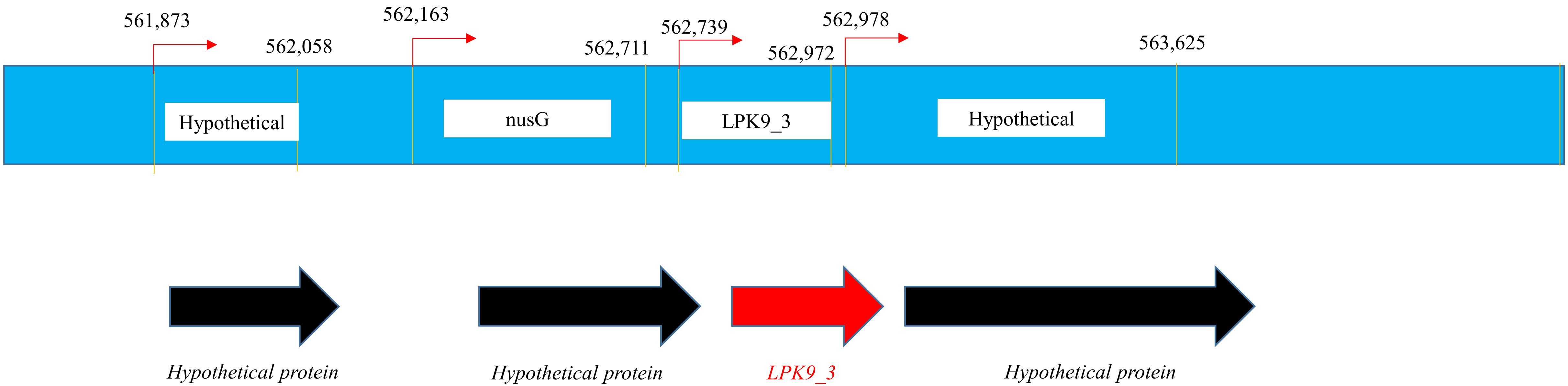

nusG; Transcription termination/antitermination protein NusG

Suppl. Fig. S15. Prediction of signal sequence for LPK9 AMP4

Name	amino acid seq	amino acid No	campsign	Others
>JKLLNNAJ_00572 hypothetical protein	MPYLNVTKGSWDKVGSTFTVKDNAEAPFTSLAGKSVVNIAMMPVQQGPWVYNTKSLSKDDQKKIATEFTSKSFAQNKKIFSEPNAKTPMMFPKKSEKSKLVNVTDKWYAPTHKLVGY	117	> DBAASP_1189|1314|CNAP2<br>Length=15<br>Score = 22.3 bits (46), Expect = 4.6<br>Identities = 7/11 (64%), Positives = 9/11 (82%), Gaps = 0/11 (0%)<br>Query 3 YLNVTKGSWDK 13<br>++NV KG WDK<br>Sbjct 2 HMNVQKGKWDK 12	Name : CNAP2 (Control peptide)<br>Synthesis Type : Synthetic<br>Target Group : Gram+ Gram-<br>Target Object : Lipid Bilayer<br>MHMNVQKGKWDKDPK<br>* NOT OVERLAPPING WITH NIBR97

	LPK9_AMPs4
total hydrophobic ratio (%)	27
total net charge	+1
MW (Da)	1310.465
Secondary structure	helix

protein type	Signal peptide (Sec/SPI)	TAT signal peptide (Tat/SPI)	Lipoprotein signal peptide (Sec/SPII)	Other
**Likelihood**	0.0137	0.0008	0.0026	0.9829

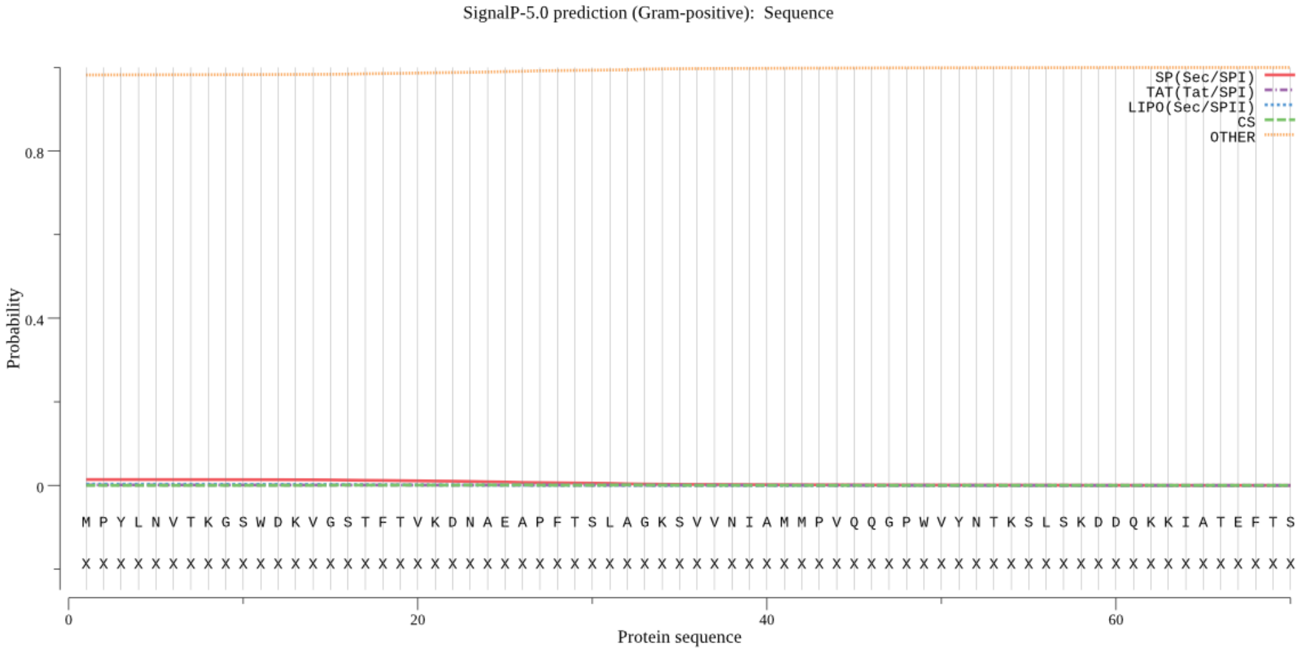

Suppl. Fig. S16. Arrangement of franking genes of LPK9 AMP4

contig 1

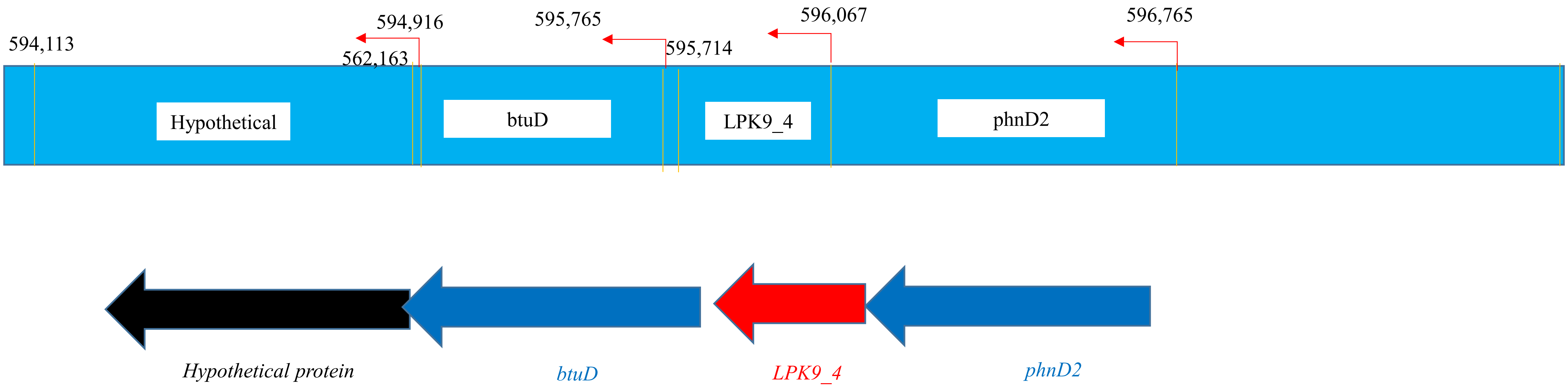

btuD; Vitamin B12 import ATP-binding protein BtuD, phnD2; putative ABC transporter phosphonate/phosphite binding protein PhnD2

Suppl. Fig. S17. Prediction of signal sequence for LPK9 AMP5

Name	amino acid seq	amino acid No	campsign	Others
>JKLLNNAJ_01374 hypothetical protein	MGWIGVAILGLTIGIVANAVGAHGRRQTTINLIAGLFGALGGQLSFGWFGPTVAGMTLLPVVSGAMILIVIGTVAAQALQPQ	82	> CAMPSQ659<br>Length=17<br>Score = 20.8 bits (42), Expect = 8.4<br>Identities = 8/14 (57%), Positives = 12/14 (86%), Gaps = 0/14 (0%)<br>Query 8 ILGLTIGIVANAVG 21<br>ILG +G+V+NA+G<br>Sbjct 1 ILGPILGLVSNALG 14	Title : Bombin H7<br>Source : Bombina orientalis [Oriental fire-bellied toad]<br>Taxonomy : Animalia, Amphibians Antibacterial<br>ILGPILGLVSNALGGLL
			> CAMPSQ3403<br>Length=60<br>Score = 23.5 bits (49), Expect = 1.3<br>Identities = 13/36 (36%), Positives = 19/36 (53%), Gaps = 3/36 (8%)<br>Query 10 GLTIGI---VANAVGAHGRRQTTINLIAGLFGALGG 42<br>G+ G+ + NAV A T ++L +G F A GG<br>Sbjct 5 GMAAGVAKTIVNAVSAGMDIATALSLFSGAFTAAGG 40	Title : Garvicin ML<br>GenInfo Identifier : 280983598<br>Source : Lactococcus garvieae DCC43 Antimicrobial<br>LVATGMAAGVAKTIVNAVSAGMDIATALSLFSGAFTAAGGIMALIKKYAQKKLWKQLIAA

	Bombin H7	Garvicin ML
total hydrophobic ratio (%)	66	48
total net charge	0	+2.25
MW (Da)	1651.028	3189.72
Secondary structure	helix	helix

protein type	Signal peptide (Sec/SPI)	TAT signal peptide (Tat/SPI)	Lipoprotein signal peptide (Sec/SPII)	Other
**Likelihood**	0.071	0.0039	0.1254	0.7996

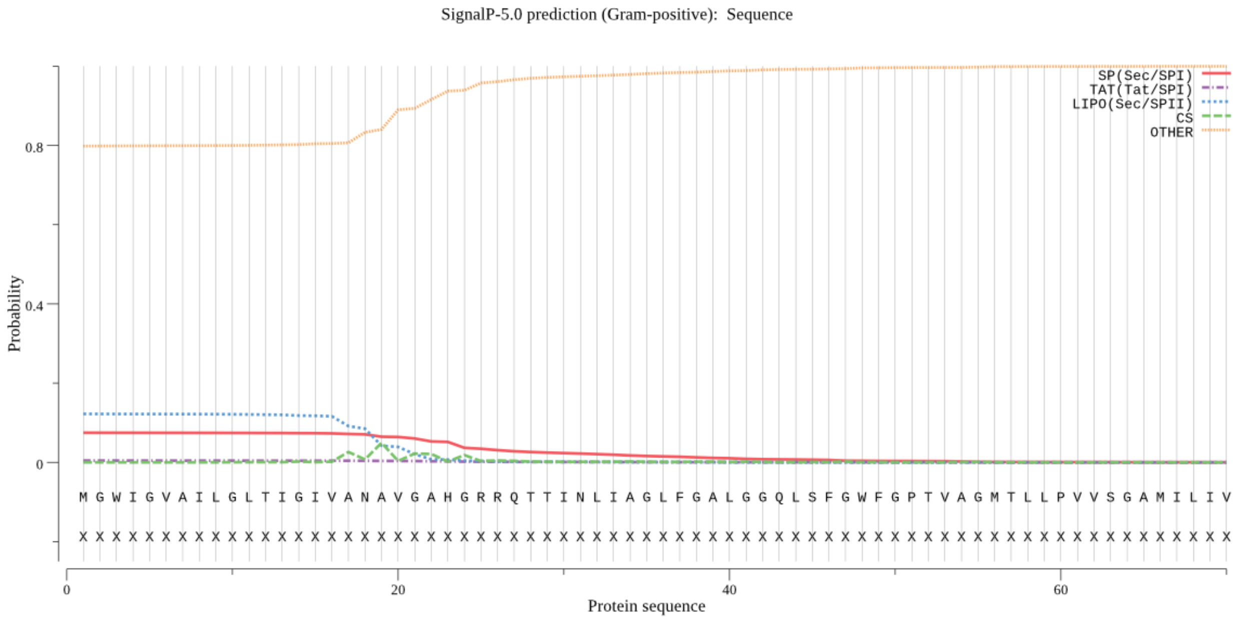

Suppl. Fig. S18. Prediction of signal sequence for LPK9 AMP5

Name	amino acid seq	amino acid No	campsign	Others
>JKLLNNAJ_01374 hypothetical protein	MGWIGVAILGLTIGIVANAVGAHGRRQTTINLIAGLFGALGGQLSFGWFGPTVAGMTLLPVVSGAMILIVIGTVAAQALQPQ	82	> CAMPSQ659<br>Length=17<br>Score = 20.8 bits (42), Expect = 8.4<br>Identities = 8/14 (57%), Positives = 12/14 (86%), Gaps = 0/14 (0%)<br>Query 8 ILGLTIGIVANAVG 21<br>ILG +G+V+NA+G<br>Sbjct 1 ILGPILGLVSNALG 14	Title : Bombin H7<br>Source : Bombina orientalis [Oriental fire-bellied toad]<br>Taxonomy : Animalia, Amphibians Antibacterial<br>ILGPILGLVSNALGGLL
			> CAMPSQ3403<br>Length=60<br>Score = 23.5 bits (49), Expect = 1.3<br>Identities = 13/36 (36%), Positives = 19/36 (53%), Gaps = 3/36 (8%)<br>Query 10 GLTIGI---VANAVGAHGRRQTTINLIAGLFGALGG 42<br>G+ G+ + NAV A T ++L +G F A GG<br>Sbjct 5 GMAAGVAKTIVNAVSAGMDIATALSLFSGAFTAAGG 40	Title : Garvicin ML<br>GenInfo Identifier : 280983598<br>Source : Lactococcus garvieae DCC43 Antimicrobial<br>LVATGMAAGVAKTIVNAVSAGMDIATALSLFSGAFTAAGGIMALIKKYAQKKLWKQLIAA

	AMP prediction		
MGWIGVAILGLTIGIVANAVGAHG	PeptideRanker		0.97791
	CAMR3	SVM	AMP(0.567)
		RFC	AMP(0.6565)
		ANN	AMP
		DAC	AMP(0.844)

Suppl. Fig. S19. Arrangement of franking genes of LPK9 AMP5

contig 1

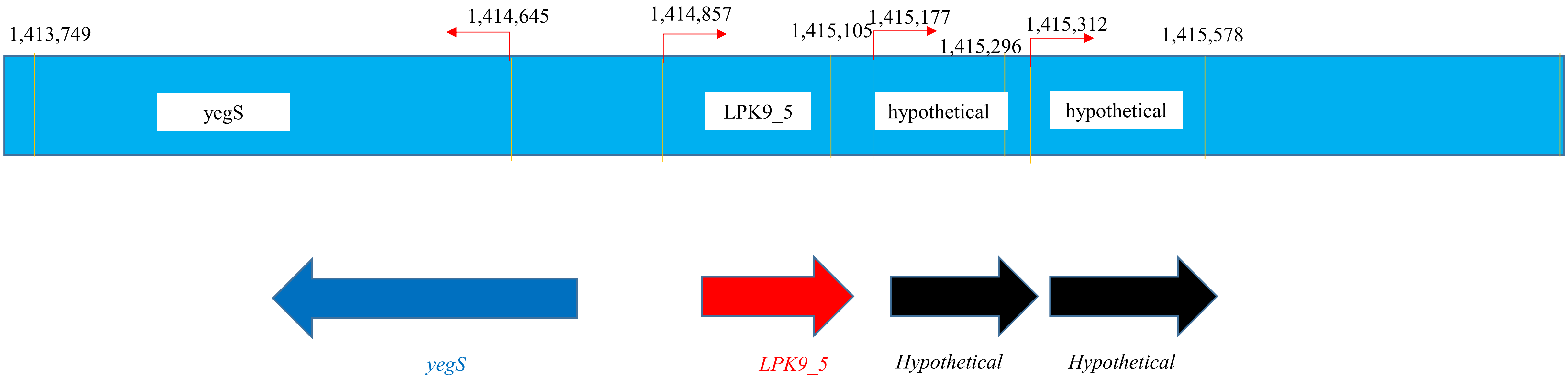

yegS; lipid kinase YegS

Suppl. Fig. S20. Prediction of signal sequence for LPK9 AMP6

<table>
<tr><th>Name</th><th>amino acid seq</th><th>amino acid No</th><th>campsign</th><th>Others</th></tr>
<tr><td rowspan="2">>JKLLNNAJ_01523 hypothetical protein</td><td rowspan="2">MSQKNDNDKSQADKPWDKTFEDDRDDSGNLSRTQKRKQDSSNSTLTTVLVVLILLLALAPIGYFLVKKNSLNNPQQTEQVASSSSKKSSASVAASKSSTAASKKKAAASSKKAAAKSSSLALASSKAASSKAASSTAASSEESASTDDSSASSSSSSSESSSGTKYVTVEAGQGVYRVATNAGISVDKLLELNGLSSDATISAGQRLRVR</td><td rowspan="2">210</td><td>> CAMPSQ85
Length=69
Score = 35.4 bits (80), Expect = 4e-004
Identities = 23/64 (36%), Positives = 34/64 (53%), Gaps = 3/64 (5%)
Query 81 ASSSSKKSSASVAASKSSTAASKKKAAASSKKAAAKSSSLALASSKAASSKAASSTAASS 140
A S KK+ K++ + K K A++ K AAKS A +KAA KAA AA +
Sbjct 5 AKKSPKKAKKPATPKKAAKSPKKVKKPAAAAKKAAKSPKKA---TKAAKPKAAKPKAAKA 61

Query 141 EESA 144
+++A
Sbjct 62 KKAA 65</td><td>Title : Oncorhyncin II
GenInfo Identifier : 121951
Source : Oncorhynchus mykiss [Rainbow trout] Antibacterial
KAVAAKKSPKKAKKPATPKKAAKSPKKVKKPAAAAKKAAKSPKKATKAAKPKAAKPKAAKAKKAAPKKK</td></tr>
<tr><td>> CAMPSQ1089
Length=30
Score = 23.1 bits (48), Expect = 4.8
Identities = 12/24 (50%), Positives = 14/24 (58%), Gaps = 0/24 (0%)
Query 90 ASVAASKSSTAASKKKAAASSKKA 113
A A+ + A KKKAAA KKA
Sbjct 4 APAPAAAAPAKAPKKKAAAKPKKA 27</td><td>Title : SAMPH1
GenInfo Identifier : 92090603
Source : Salmo salar [Atlantic salmon] Antibacterial
AEVAPAPAAAAPAKAPKKKAAAKPKKAGPS</td></tr>
</table>

	Oncorhyncin II	SAMPH1
total hydrophobic ratio (%)	37	41
total net charge	+9	+6
MW (Da)	5883.389	2236.53
Secondary structure	helix	helix

protein type	Signal peptide (Sec/SPI)	TAT signal peptide (Tat/SPI)	Lipoprotein signal peptide (Sec/SPII)	Other
**Likelihood**	0058	0.0057	0.0074	0.9812

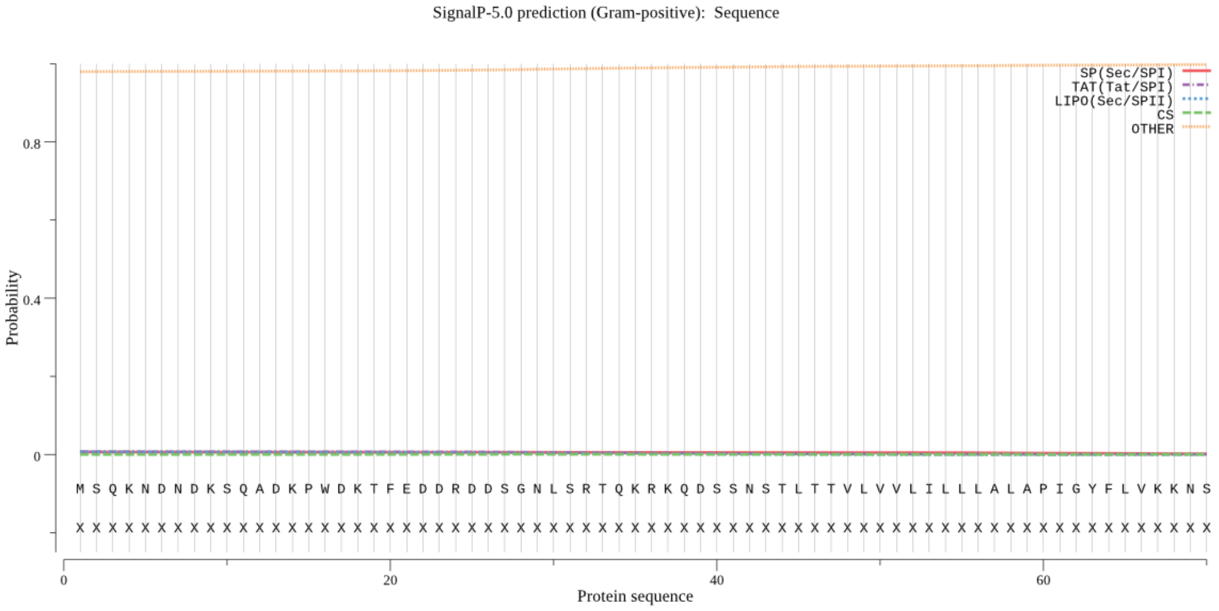

Suppl. Fig. S21. Arrangement of franking genes of LPK9 AMP6

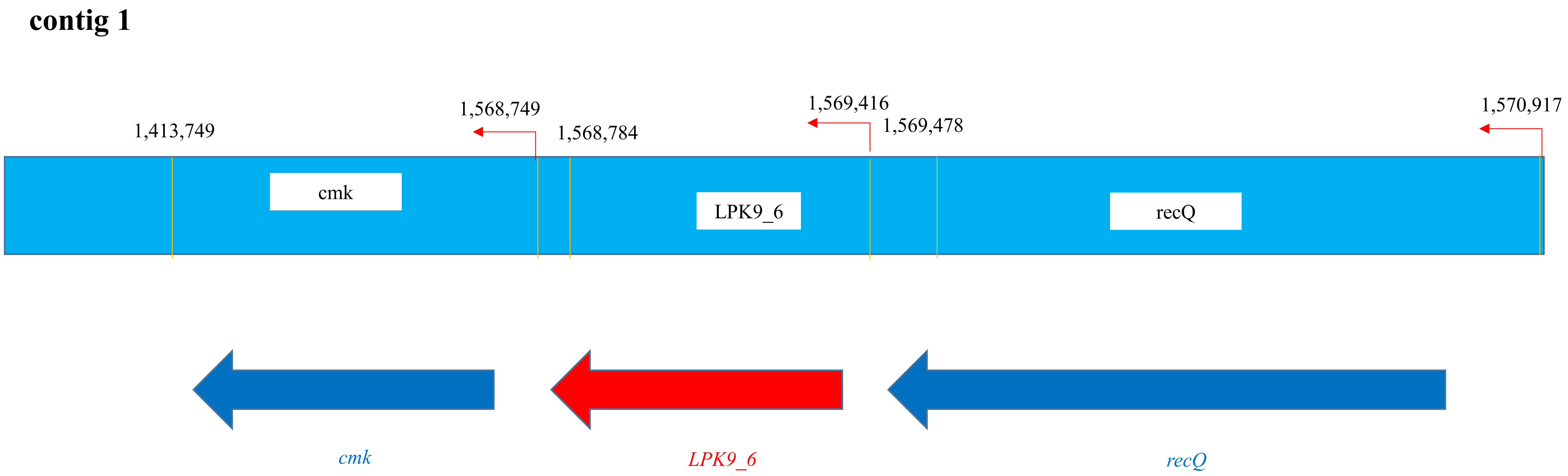

cmK; Cytidylate kinase, recQ; ATP-dependent DNA helicase RecQ

Suppl. Fig. S22. Prediction of signal sequence for LPK9 AMP7

Name	amino acid seq	amino acid No	campsign	Others
>JKLLNNAJ_01715 hypothetical protein	MGLVYASVRILSNRRVQTLEYLADELMLTSLGIINHQQPVTATHDQPAIKPQPVTSVVKAAVKRV	65	> DBAASP_4301|4651|Buf III analog<br>Length=21<br>Score = 20.0 bits (40), Expect = 10.0<br>Identities = 8/17 (47%), Positives = 11/17 (65%), Gaps = 0/17 (0%)<br>Query 49 IKPQPVTSVVKAAVKRV 65<br>+K P+ VVK VK+V<br>Sbjct 3 VKQWPIGKVVKKVVKKV 19	Name : Buf III analog<br>Synthesis Type : Synthetic<br>Target Group : Gram+Gram-FungusMammalian Cell<br>Target Object : DNA / RNA<br>KVVKQWPIGKVVKKVVKKVVK<br>* NOT OVERLAPPING NIBR97

total hydrophobic ratio (%)	47
total net charge	+4
MW (Da)	1820.244
Secondary structure	helix

protein type	Signal peptide (Sec/SPI)	TAT signal peptide (Tat/SPI)	Lipoprotein signal peptide (Sec/SPII)	Other
**Likelihood**	0.0197	0.0061	0.0015	0.9728

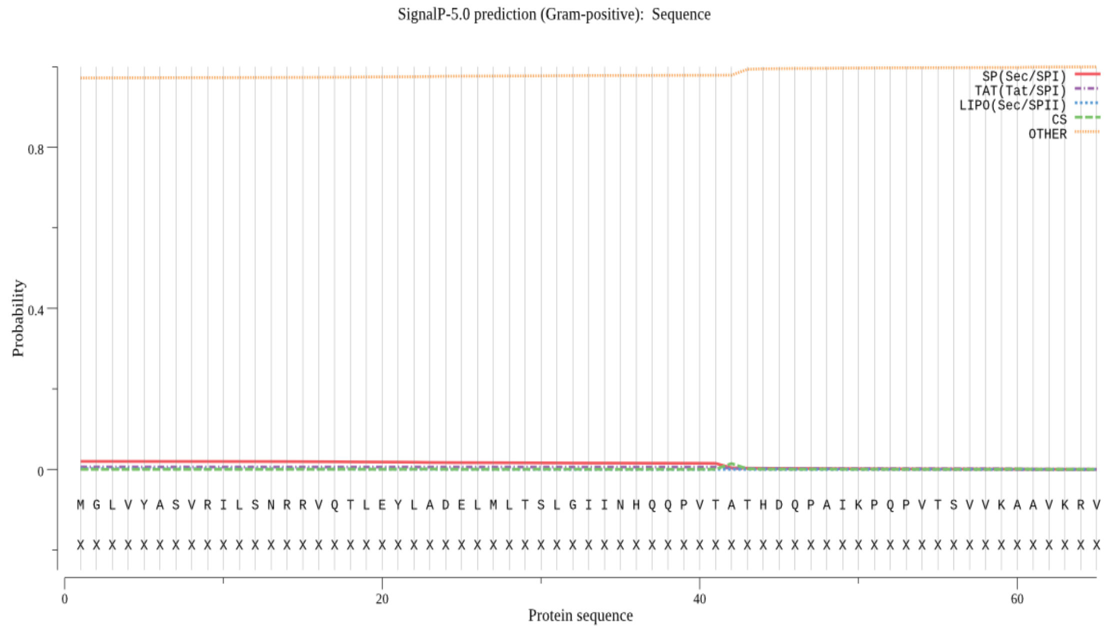

Suppl. Fig. S23. Prediction of signal sequence for LPK9 AMP7

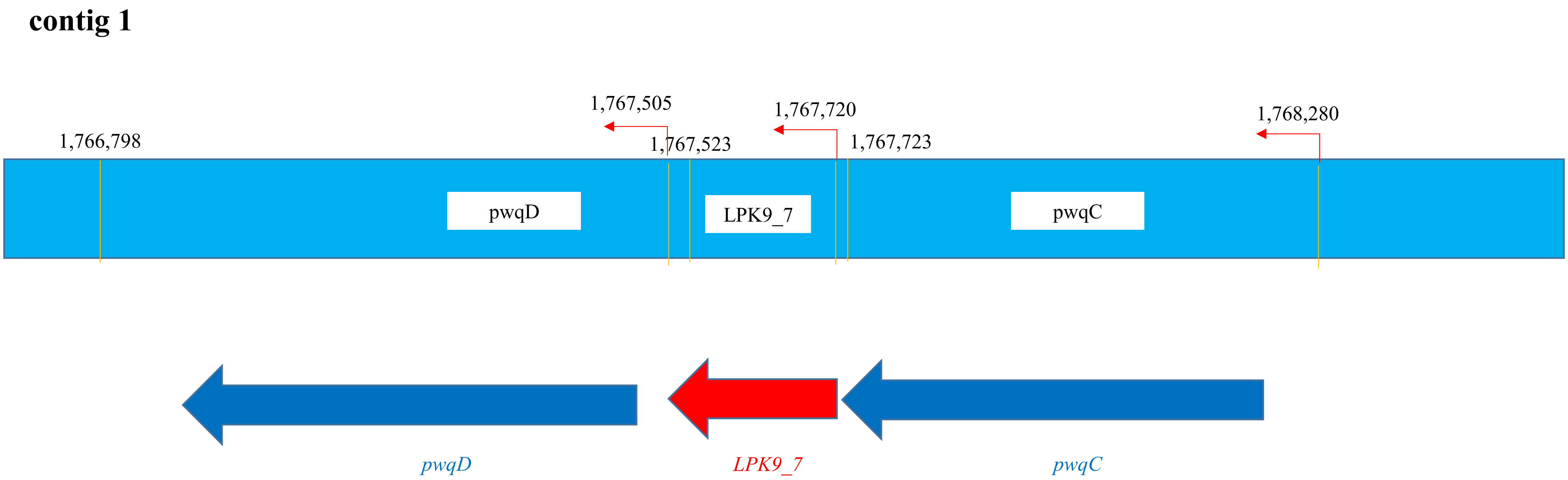

pwqD; Tyrosine-protein kinase YwqD, pwqC; putative capsular polysaccharide biosynthesis protein YwqC

Suppl. Fig. S24. Prediction of signal sequence for LPK9 AMP8

Name	amino acid seq	amino acid No	campsign	Others
>JKLLNNAJ_01850 hypothetical protein	MNREHIDELAEQQAERRRNLYHGLRTDKTHFEVSKHP<br>FEIVYDYRQGFDLDKFVERYSSILNKYDYIVGDWGFE<br>QLRLKGFFRDDMKDVQRSQTIGAVQDYLYEYCNFGCA<br>YFIIKNERVIKPKRTSRSRKDDRRGKKNTNSRQSRAR<br>SNRNSSRSKQRRKSHSFTTKQKAAPFTEKRRQPAKVT<br>AGNGKQAQTTKQSSGKRHFTIRQK	209	> DBAASP_6067|6422|Hemoglobin subunit alpha<br>Length=40<br>Score = 23.5 bits (49), Expect = 3.5<br>Identities = 11/33 (33%), Positives = 17/33 (52%), Gaps = 0/33 (0%)<br>Query 4 EHIDELAEQQAERRRNLYHGLRTDKTHFEVSKH 36<br>EH+D+L +E H LR D +F++ H<br>Sbjct 5 EHLDDLPGALSELSDLHAHKLRVDPVNFKLLSH 37	Name : Hemoglobin subunit alpha (67-106)<br>Synthesis Type : Synthetic<br>Target Group : Gram+ Gram-<br>Target Object : Lipid Bilayer<br>TKAVEHLDDLPGALSELSDLHAHKLRVDPVNFKLLSHSLL
			> Yadamp_1670|2500|Protamine y1<br>Length=31<br>Score = 22.3 bits (46), Expect = 8.5<br>Identities = 10/24 (42%), Positives = 16/24 (67%), Gaps = 0/24 (0%)<br>Query 147 ARSNRNSSRSKQRRKSHSFTTKQK 170<br>AR R+SSR +RR+ TT+++<br>Sbjct 1 ARRRRSSSRPIRRRRPRRRTTRRR 24	acitivity: ?<br>ARRRRSSSRPIRRRRPRRRTTRRRRGARRRR

	hemoglobin	Protamine yl
total hydrophobic ratio (%)	24	8
total net charge	0	+9.25
MW (Da)	4044.419	2876.18
Secondary structure	helix	

protein type	Signal peptide (Sec/SPI)	TAT signal peptide (Tat/SPI)	Lipoprotein signal peptide (Sec/SPII)	Other
**Likelihood**	0.0055	0.0015	0.0016	0.9913

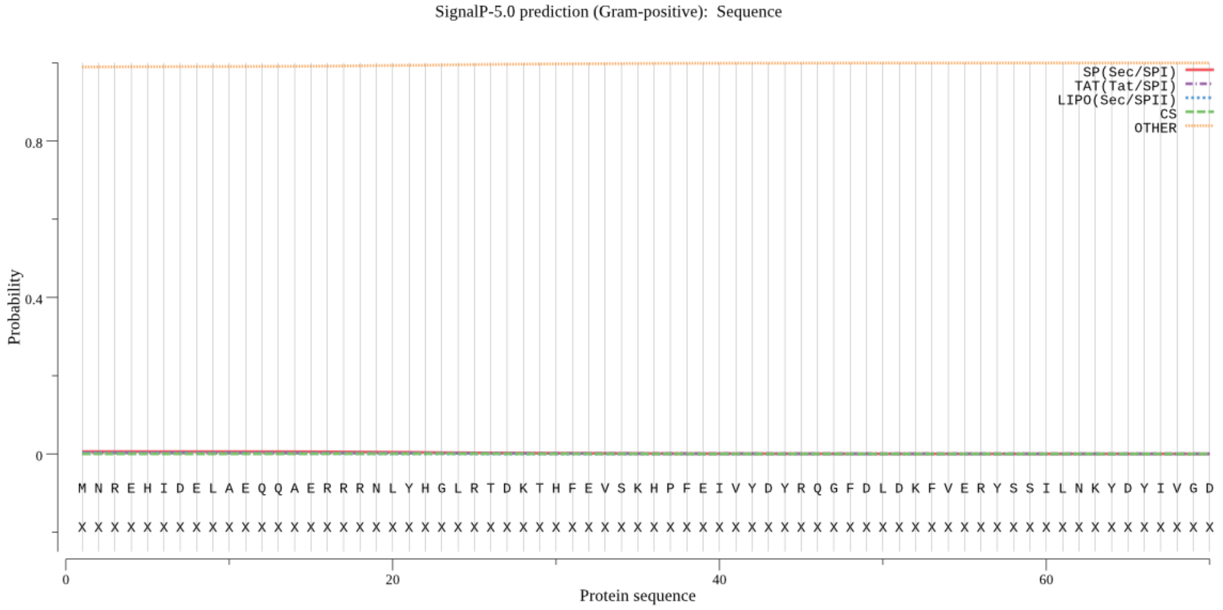

Suppl. Fig. S25. Prediction of signal sequence for LPK9AMP8

contig 1

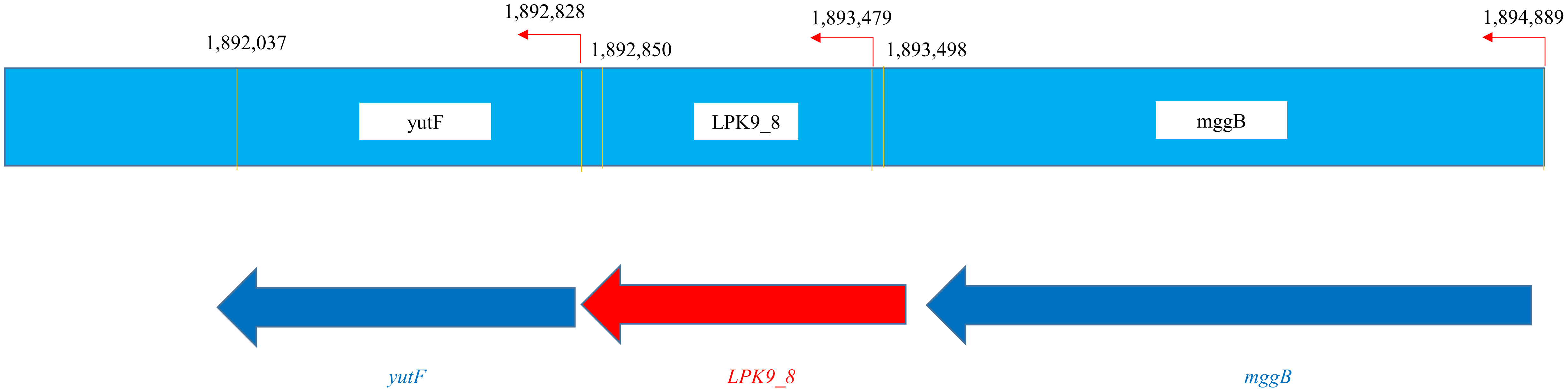

yutF; Acid sugar phosphatase, mggB; Mannosylglucosyl-3-phosphoglycerate phosphatase

Suppl. Fig. S26. Prediction of signal sequence for LPK9 AMP9

Name	amino acid seq	amino acid No	campsign	Others
>JKLLNNAJ_02064 hypothetical protein	MSFFNDFHVLPGTHTSSYTSSVSNDIFGGFHSFILNLIPAIKSLFSK	47	> CAMPSQ2603<br><br>Length=24<br><br>Score = 19.6 bits (39), Expect = 7.3<br>Identities = 8/21 (38%), Positives = 12/21 (57%), Gaps = 0/21 (0%)<br>Query 27 FGGFHSFILNLIPAIKSLFSK 47<br>FG S L+P++ LFS+<br>Sbjct 1 FGSLFSLGSKLLPSVFKLFSR 21	Title : Ponericin-W-like 321<br>Source : Lychas mucronatus [Chinese swimming scorpion]<br>Taxonomy : Animalia, Arachnids<br>Length : 24<br>Activity : Antibacterial, Antifungal<br>FGSLFSLGSKLLPSVFKLFSRKKQ

total hydrophobic ratio (%)	54
total net charge	+2.25
MW (Da)	2449.962
Secondary structure	helix

protein type	Signal peptide (Sec/SPI)	TAT signal peptide (Tat/SPI)	Lipoprotein signal peptide (Sec/SPII)	Other
**Likelihood**	0.0478	0.0044	0.0162	0.9316

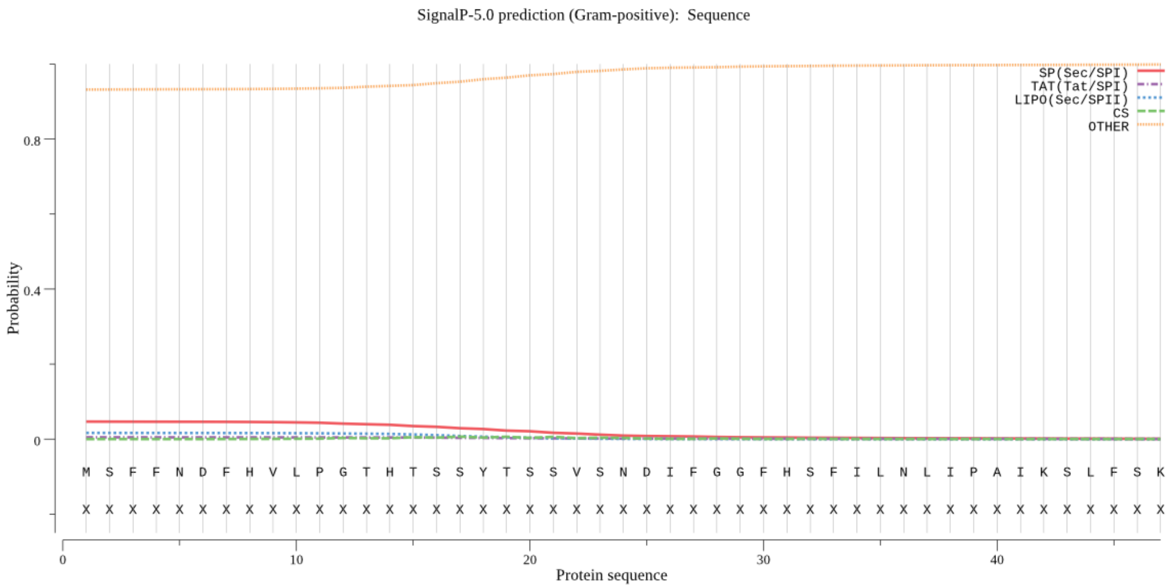

Suppl. Fig. S27. Prediction of signal sequence for LPK9 AMP9

contig 1

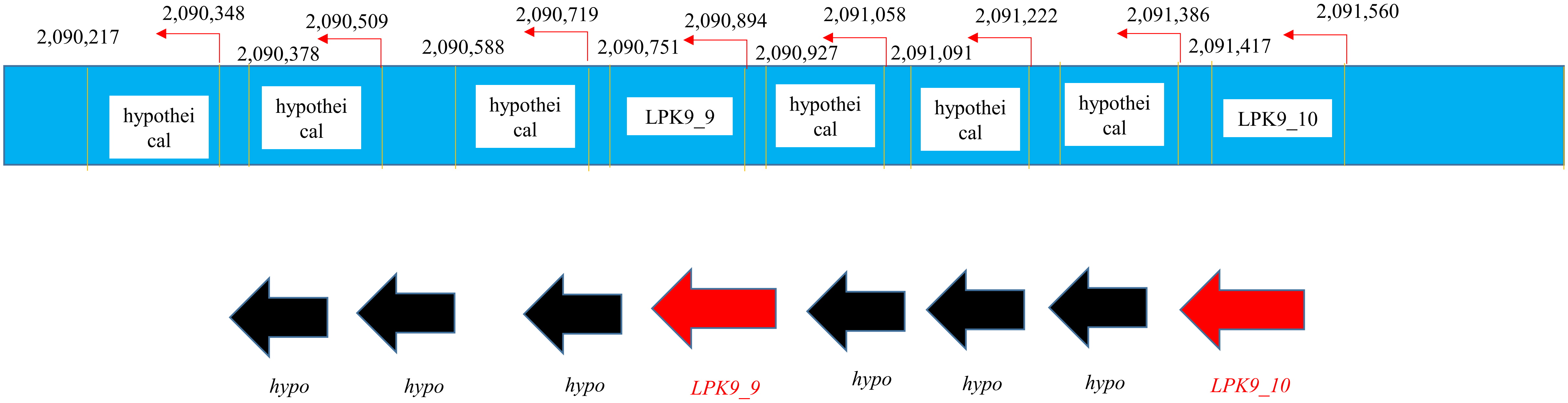

Suppl. Fig. S28. Prediction of signal sequence for LPK9 AMP10

Name	amino acid seq	amino acid No	campsign	Others
>JKLLNNAJ_02068 hypothetical protein	MSFFTDLHFLPGTHTSSYTSSVSNDIFGGFHSFILNLIPAIKSLFSK	47	> CAMPSQ2603<br>Length=24<br>Score = 19.6 bits (39), Expect = 7.3<br>Identities = 8/21 (38%), Positives = 12/21 (57%), Gaps = 0/21 (0%)<br>Query 27 FGGFHSFILNLIPAIKSLFSK 47<br>FG S L+P++ LFS+<br>Sbjct 1 FGSLFSLGSKLLPSVFKLFSR 21	Title : Ponericin-W-like 321<br>Source : Lychas mucronatus [Chinese swimming scorpion]<br>Taxonomy : Animalia, Arachnids<br>Length : 24<br>Activity : Antibacterial, Antifungal<br>FGSLFSLGSKLLPSVFKLFSRKKQ

total hydrophobic ratio (%)	52
total net charge	+2.25
MW (Da)	2336.802
Secondary structure	helix

```
              10        20        30        40
               |         |         |         |
AMPs9       MSFFNDFHVLPGTHTSSYTSSVSNDIFGGFHSFILNLIPAIKSLFSK
AMPs10      MSFFTDLHFLPGTHTSSYTSSVSNDIFGGFHSFILNLIPAIKSLFSK
            ****.*:*.**************************************
Prim.cons.  MSFF2D2H2LPGTHTSSYTSSVSNDIFGGFHSFILNLIPAIKSLFSK
```

protein type	Signal peptide (Sec/SPI)	TAT signal peptide (Tat/SPI)	Lipoprotein signal peptide (Sec/SPII)	Other
**Likelihood**	0.0451	0.0051	0.0185	0.9313

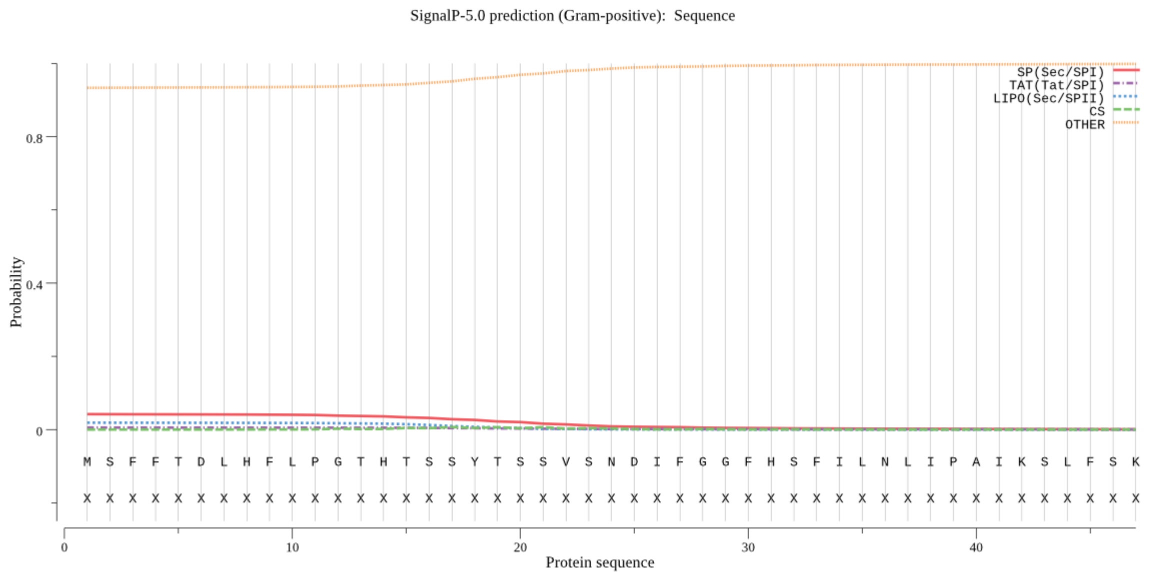

Suppl. Fig. S29. Prediction of signal sequence for LPK9 AMP10

contig 1

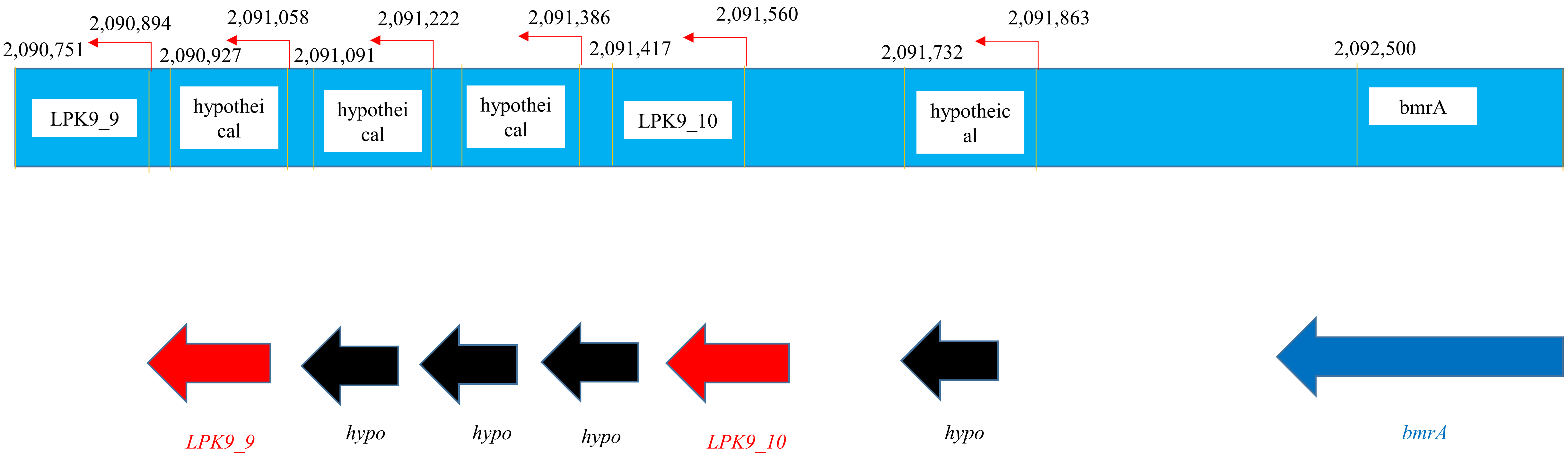

bmrA; Multidrug resistance ABC transporter ATP-binding/permease protein BmrA

Suppl. Fig. S30. Prediction of signal sequence for LPK9 AMP11

Name	amino acid seq	amino acid No	campsign	Others
>JKLLNNAJ_02183 hypothetical protein	MKTTWLASLLVTIFWGAVLGLVVTYLGGAMVEALTATPIVREPFKAMAVGIILAVMSGLLVTTHH	65	> CAMPSQ3668<br>Length=28<br>Score = 20.4 bits (41), Expect = 7.4<br>Identities = 10/25 (40%), Positives = 17/25 (68%), Gaps = 1/25 (4%)<br>Query 38 PIVREPFKAMAVGIILAVMSGLLVT 62<br>PIV++ K +A G +L+ ++G L T<br>Sbjct 5 PIVKDTLKKVAKG-VLSTIAGALST 28	Title : Hymenochirin-5B<br>Source : Hymenochirus boettgeri [Congo dwarf clawed frog]<br>Length : 28<br>Activity : Antimicrobial<br>Validated : Experimentally Validated<br>Sequence: IKIPPIVKDTLKKVAKGVLSTIAGALST
			> CAMPSQ649<br>Length=21<br>Score = 20.0 bits (40), Expect = 10.0<br>Identities = 9/15 (60%), Positives = 11/15 (73%), Gaps = 0/15 (0%)<br>Query 16 GAVLGLVVTYLGGAM 30<br>G VLGLV + LGG +<br>Sbjct 3 GPVLGLVGSALGGLL 17	Title : Bombinin-H4<br>Source : Bombina variegata [Yellow-bellied toad]<br>Length : 21<br>Activity : Antibacterial<br>LIGPVLGLVGSALGGLLKKIG

	Hymenochirin-5B	Bombinin-H4
total hydrophobic ratio (%)	64	60
total net charge	+1	0
MW (Da)	2626.314	1420.74
Secondary structure	helix	G rich

protein type	Signal peptide (Sec/SPI)	TAT signal peptide (Tat/SPI)	Lipoprotein signal peptide (Sec/SPII)	Other
**Likelihood**	0.068	0.0012	0.0183	0.9125

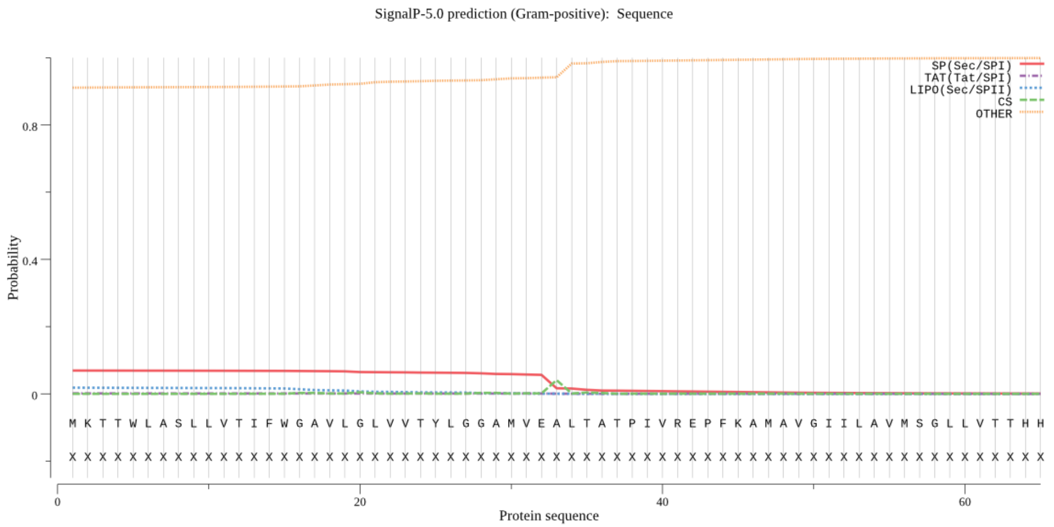

Suppl. Fig. S31. Prediction of signal sequence for LPK9 AMP11

contig 1

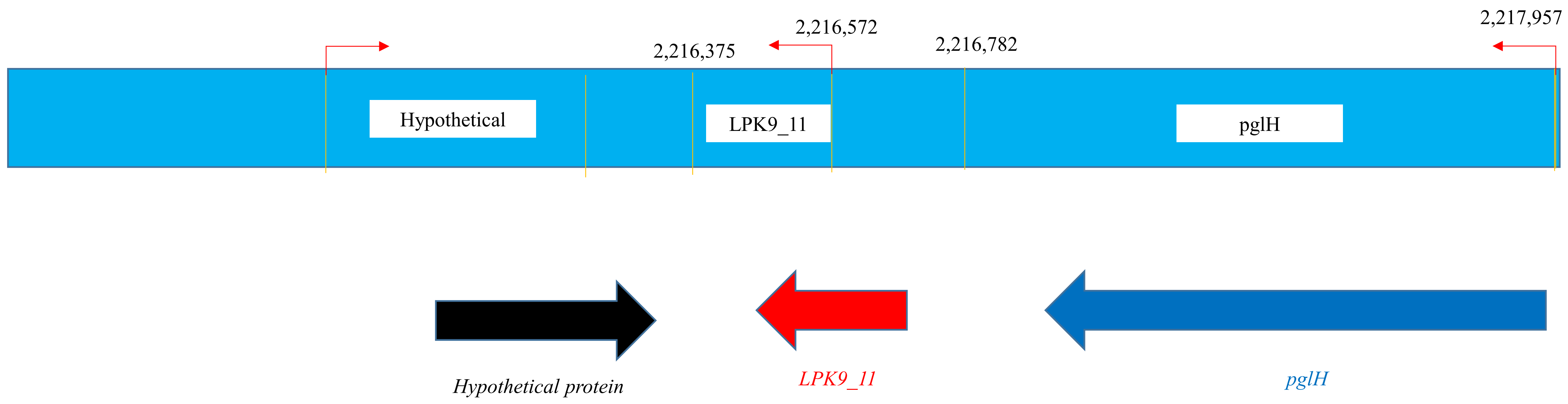

pglH; GalNAc-alpha-(1->4)-GalNAc-alpha-(1->3)-diNAcBac-PP-undecaprenol alpha-1%2C4-N-acetyl-D-galactosaminyltransferase

Suppl. Fig. S32. Prediction of signal sequence for LPK9 AMP12

Name	amino acid seq	amino acid No	campsign	Others
>JKLLNNAJ_0247<br>3 hypothetical<br>protein	MKKWEKQTMKIAALGAMALTLAGCATAKSSSSQGNNP<br>GKTIKIGVNMELSGSAAGYGEQQKQGIQLAVKKINKS<br>GGIKVGGPRRKFSW	88	> CAMPSQ536<br>Length=48<br>Score = 21.6 bits (44), Expect = 6.3<br>Identities = 11/34 (32%), Positives = 17/34 (50%), Gaps = 0/34 (0%)<br>Query 24 CATAKSSSSQGNNPGKTIKIGVNMELSGSAAGYG 57<br>C+ K S + G + G+N +SG A+G G<br>Sbjct 9 CSKTKCSVNWGQAFQERYTAGINSFVSGVASGAG 42	Title : Bacteriocin carnobacteriocin B2<br>Source : Carnobacterium maltaromaticum<br>Length : 48<br>Activity : Antibacterial<br>VNYGNGVSCSKTKCSVNWGQAFQERYTAGINSFVSGVASGA<br>GSIGRRP<br>* NOT OVERLAPPING WITH NIBR97
			> CAMPSQ861<br>Length=24<br>Score = 20.8 bits (42), Expect = 8.9<br>Identities = 7/14 (50%), Positives = 12/14 (86%), Gaps = 0/14 (0%)<br>Query 68 VKKINKSGGIKVGG 81<br>+++I K+GGI +GG<br>Sbjct 6 LERIGKAGGIIGG 19	Title : Winter flounder 1<br>Source : Pseudopleuronectes americanus [Winter flounder]<br>Length : 24<br>Activity : Antibacterial, Antifungal<br>GKGRWLERIGKAGGIIGGALDHL

	Carnobacteriocin B2	Winter flounder 1
total hydrophobic ratio (%)	29	28
total net charge	+2	+4
MW (Da)	3286.64	1384.671
Secondary structure	helix	G rich

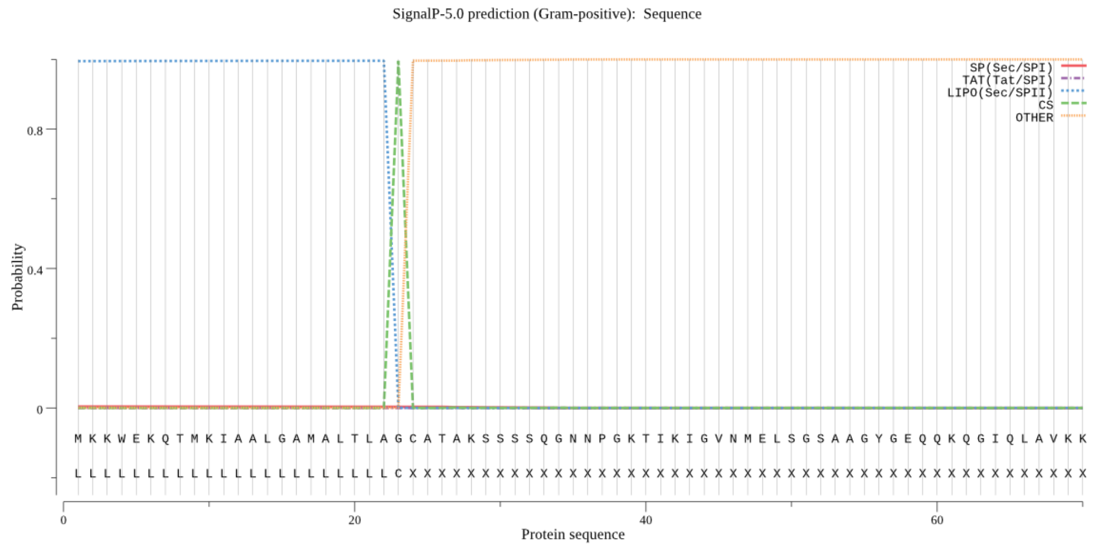

Protein type	Signal peptide (Sec/SPI)	TAT signal peptide (Tat/SPI)	Lipoprotein signal Peptide (Sec/SPII)	Other
**Likelihood**	0.0038	0.0002	0.9958	0.0003

Suppl. Fig. S33. Prediction of signal sequence for LPK9 AMP12

Name	amino acid seq	amino acid No	campsign	Others
>JKLLNNAJ_02473 hypothetical protein	MKKWEKQTMKIAALGAMALTLAGCATAKSSSSQGNNPGKTIKIGVNMELSGSAAGYGEQQKQGIQLAVKKINKSGGIKVGGPRRKFSW	88	> CAMPSQ536 Length=48<br>Score = 21.6 bits (44), Expect = 6.3<br>Identities = 11/34 (32%), Positives = 17/34 (50%), Gaps = 0/34 (0%)<br>Query 24 CATAKSSSSQGNNPGKTIKIGVNMELSGSAAGYG 57<br>C+ K S + G + G+N +SG A+G G<br>Sbjct 9 CSKTKCSVNWGQAFQERYTAGINSFVSGVASGAG 42	Title : Bacteriocin carnobacteriocin B2<br>Source : Carnobacterium maltaromaticum<br>Length : 48<br>Activity : Antibacterial<br>VNYGNGVSCSKTKCSVNWGQAFQERYTAGINSFVSGVASGAGSIGRRP<br>* NOT OVERLAPPING WITH NIBR97
			> CAMPSQ861 Length=24<br>Score = 20.8 bits (42), Expect = 8.9<br>Identities = 7/14 (50%), Positives = 12/14 (86%), Gaps = 0/14 (0%)<br>Query 68 VKKINKSGGIKVGG 81<br>+++I K+GGI +GG<br>Sbjct 6 LERIGKAGGIIGG 19	Title : Winter flounder 1<br>Source : Pseudopleuronectes americanus [Winter flounder]<br>Length : 24<br>Activity : Antibacterial, Antifungal<br>GKGRWLERIGKAGGIIGGALDHL

SignalP-5.0 (https://services.healthtech.dtu.dk/services/SignalP-5.0/)

Suppl. Fig. S34. Prediction of signal sequence for LPK9 AMP12

Name	amino acid seq	amino acid No	campsign	Others
>JKLLNNAJ_02473 hypothetical protein	MKKWEKQTMKIAALGAMALTLAGCATAKSSSSQGNNPGKTIKIGVNMELSGSAAGYGEQQKQGIQLAVKKINKSGGIKVGGPRRKFSW	88	> CAMPSQ536 Length=48<br>Score = 21.6 bits (44), Expect = 6.3<br>Identities = 11/34 (32%), Positives = 17/34 (50%), Gaps = 0/34 (0%)<br>Query 24 CATAKSSSSQGNNPGKTIKIGVNMELSGSAAGYG 57<br>C+ K S + G + G+N +SG A+G G<br>Sbjct 9 CSKTKCSVNWGQAFQERYTAGINSFVSGVASGAG 42	Title : Bacteriocin carnobacteriocin B2<br>Source : Carnobacterium maltaromaticum<br>Length : 48<br>Activity : Antibacterial<br>VNYGNGVSCSKTKCSVNWGQAFQERYTAGINSFVSGVASGAGSIGRRP<br>* NOT OVERLAPPING WITH NIBR97
			> CAMPSQ861 Length=24<br>Score = 20.8 bits (42), Expect = 8.9<br>Identities = 7/14 (50%), Positives = 12/14 (86%), Gaps = 0/14 (0%)<br>Query 68 VKKINKSGGIKVGG 81<br>+++I K+GGI +GG<br>Sbjct 6 LERIGKAGGIIGG 19	Title : Winter flounder 1<br>Source : Pseudopleuronectes americanus [Winter flounder]<br>Length : 24<br>Activity : Antibacterial, Antifungal<br>GKGRWLERIGKAGGIIGGALDHL

Suppl. Fig. S35. Prediction of signal sequence for LPK9 AMP12

contig 1

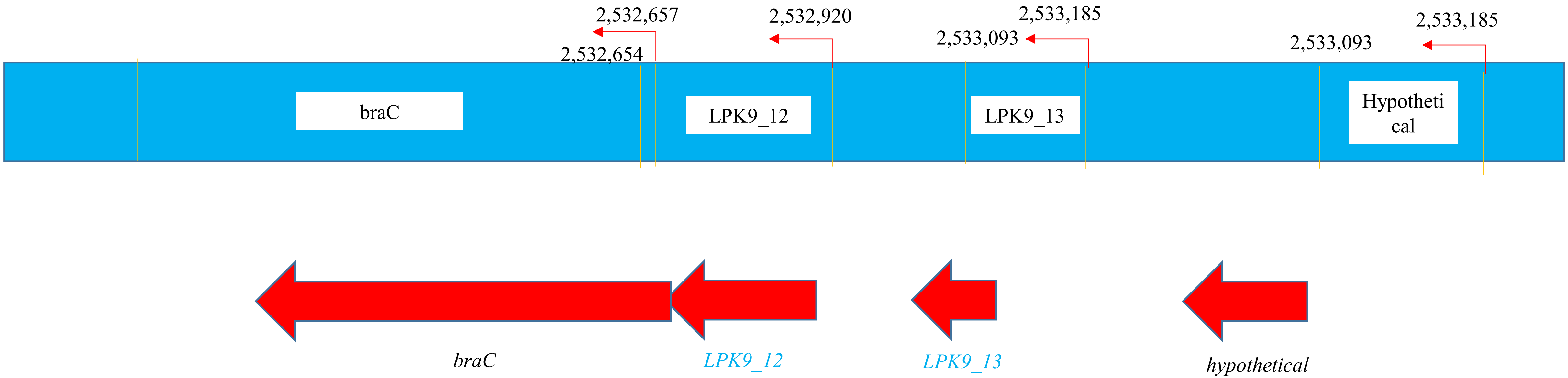

braC; Leucine-, isoleucine-,C valine-, threonine-, and alanine-binding protein

Suppl. Fig. S36. Prediction of signal sequence for LPK9 AMP13

Name	amino acid seq	amino acid No	campsign	Others
>JKLLNNAJ_02474 hypothetical protein	MKIQSNLTNLLTTLAVQNLALLLMKKDPVT	30	> DBAASP_4173|4522|D-LAK140<br>Length=25<br>Score = 19.2 bits (38), Expect = 4.8<br>Identities = 10/22 (45%), Positives = 14/22 (64%), Gaps = 0/22 (0%)<br>Query 5 SNLTNLLTTLAVQNLALLLMKK 26<br>+ L L LA++ LALL +KK<br>Sbjct 4 AKLALLKWLLALKKLALLALKK 25	Name : D-LAK140<br>Synthesis Type : Synthetic<br>Target Group : Gram+Gram-<br>Target Object : Lipid Bilayer<br>KKLAKLALLKWLLALKKLALLALKK

total hydrophobic ratio (%)	54
total net charge	+2
MW (Da)	2412.961
Secondary structure	helix

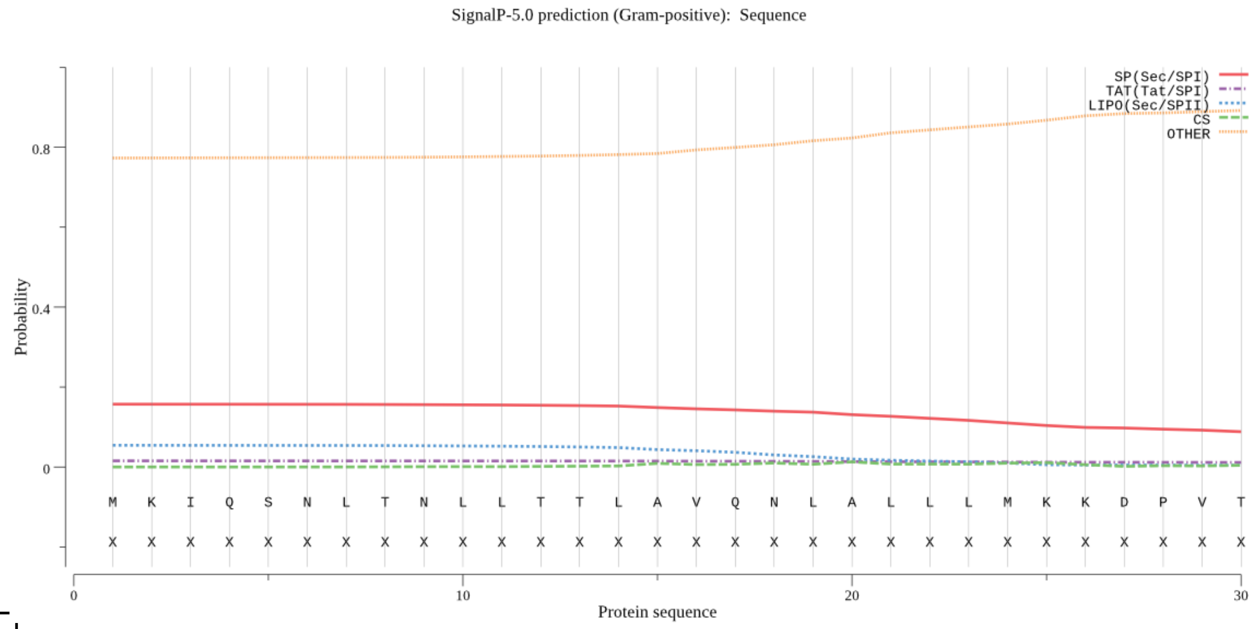

Protein type	Signal peptide (Sec/SPI)	TAT signal peptide (Tat/SPI)	Lipoprotein signal Peptide (Sec/SPII)	Other
**Likelihood**	0.157	0.0153	0.0537	0.774

Suppl. Fig. S37. Prediction of signal sequence for LPK9 AMP13

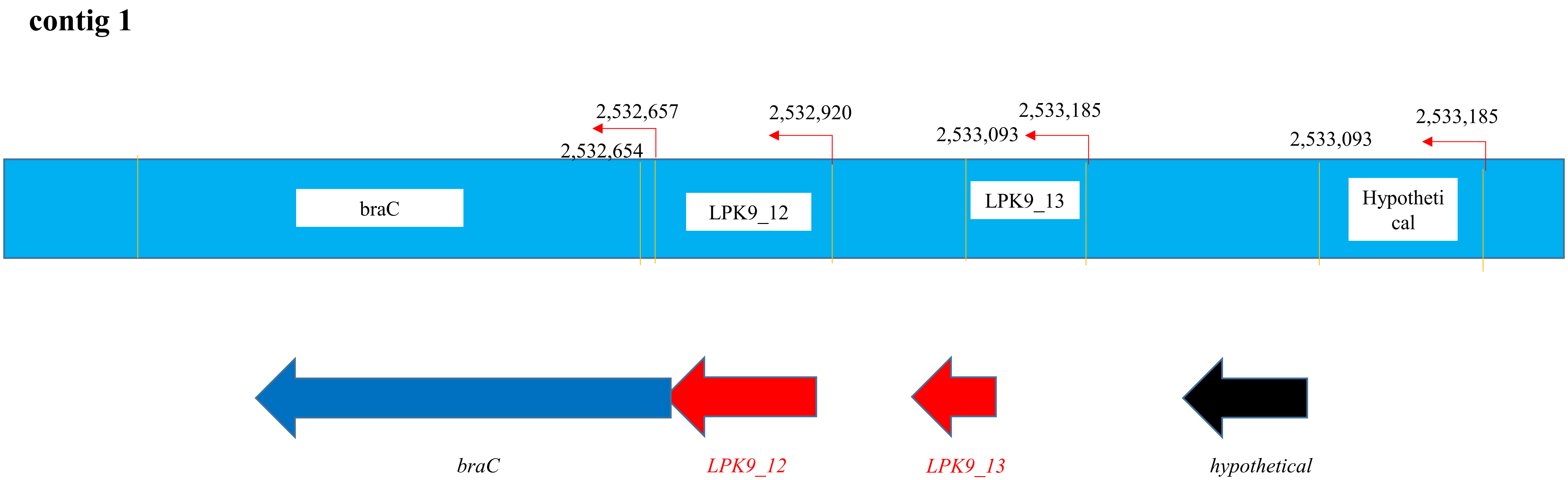

braC; Leucine-, isoleucine-,C valine-, threonine-, and alanine-binding protein

Suppl. Fig. S38. Prediction of signal sequence for LPK9 AMP14

Name	amino acid seq	amino acid No	campsign	Others
>JKLLNNAJ_02712 hypothetical protein	MDNIGYLLMQFSKQLRYQLNQRLIANGLTIQQWAVMQQISLWVERTAQQPTANQLCHVLDMDRPTMSGILRRLAAKRLVEQEVNPADQRAKLLRLTQVGIQELQAGQRISDQVVATGLQKLTAAEKQSLRQLLKKLGSN	139	> CAMPSQ59<br>Length=36<br>Score = 23.1 bits (48), Expect = 2.7<br>Identities = 9/26 (35%), Positives = 16/26 (62%), Gaps = 0/26 (0%)<br>Query 104 QAGQRISDQVVATGLQKLTAAEKQSL 129<br>+AGQR+ D +++ G T A+ +L<br>Sbjct 9 RAGQRVRDAIISAGPAVATVAQATAL 34	Title : Cecropin-D<br>Length : 36<br>Activity : Antibacterial<br>WNPFKELERAGQRVRDAIISAGPAVATVAQATALAK
			> DBAASP_6046|6401| Length=48<br>Score = 22.7 bits (47), Expect = 4.1<br>Identities = 12/32 (38%), Positives = 19/32 (59%), Gaps = 0/32 (0%)<br>Query 74 AAKRLVEQEVNPADQRAKLLRLTQVGIQELQA 105<br>A K+ +E + + LL+LT GI++LQA<br>Sbjct 14 AIKKKIEAIEKEIEAQQHLLQLTVWGIKQLQA 45	Synthesis Type : Synthetic<br>Target Group : Virus<br>Target Object : gp41<br>IKKEIEAIKKEQEAIKKKIEAIEKEIEAQQHLLQLTVWGIKQLQARIL

	Cecropin-D	synthetic
total hydrophobic ratio (%)	37	43
total net charge	+1	+1
MW (Da)	2541.885	3587.143
Secondary structure	helix	helix

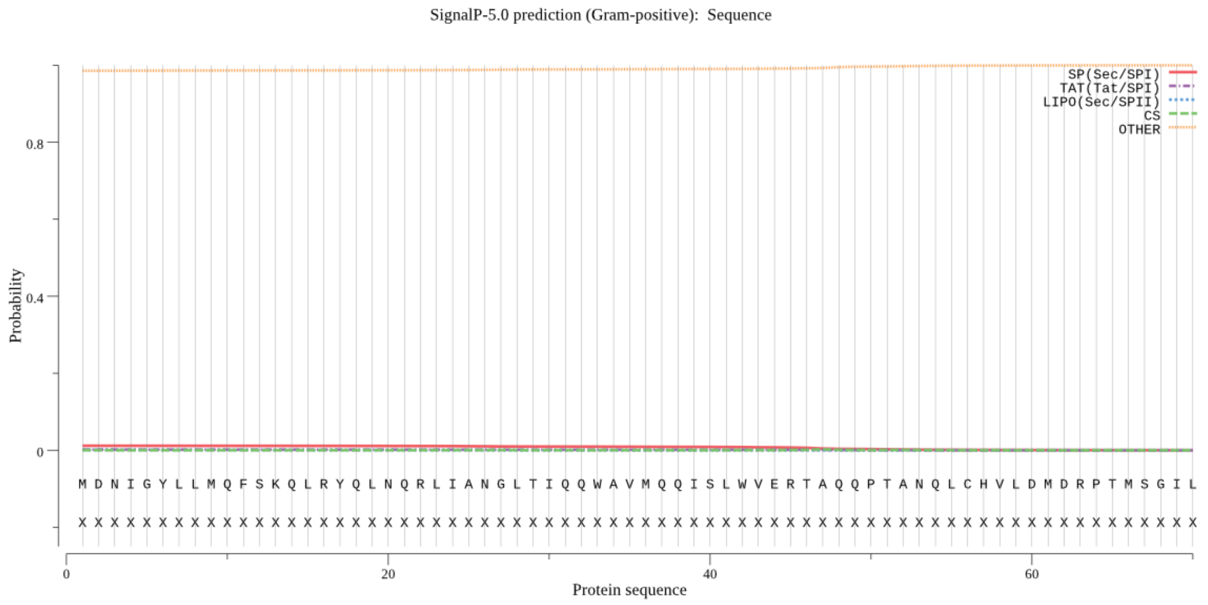

Protein type	Signal peptide (Sec/SPI)	TAT signal peptide (Tat/SPI)	Lipoprotein signal Peptide (Sec/SPII)	Other
**Likelihood**	0.0113	0.0015	0.0008	0.9864

Suppl. Fig. S39. Prediction of signal sequence for LPK9 AMP14

contig 1

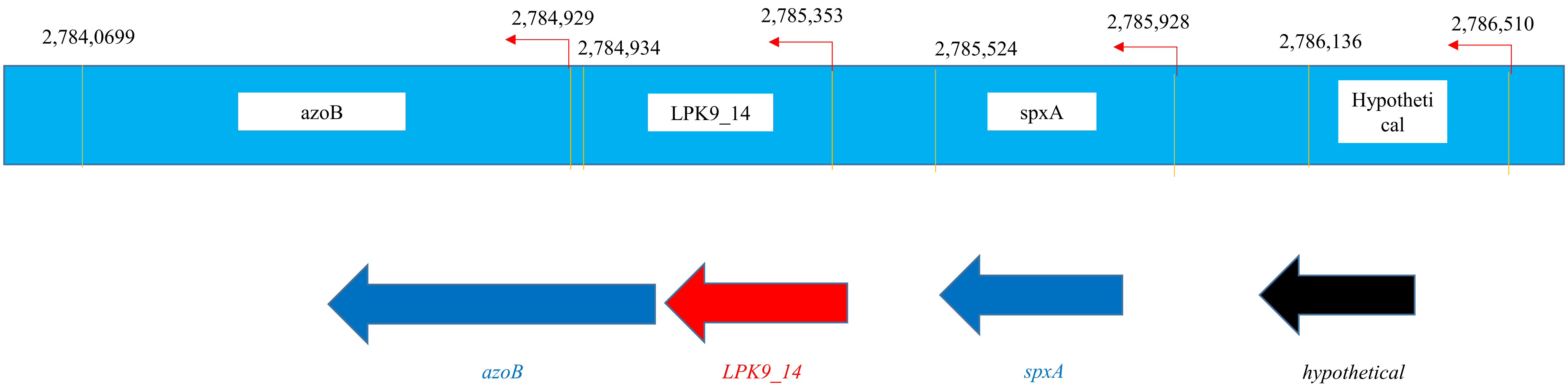

azoB; NAD(P)H azoreductase, spxA; Regulatory protein Spx

Suppl. Fig. S40. Prediction of signal sequence for LPK9 AMP15

Name	amino acid seq	amino acid No	campsign	Others
>JKLLNNAJ_02746 hypothetical protein	MSDTAVMLKTTLLAGAILLENGAEIKRVEDTMQRIVTNAGYPDAQVFVLLTGITVSLPENATSEVRAIHNRGMDLEKVDQVNSLSRQFANHDIDLTEFAAALERVNQAVPTFPFSWLCLAAVVVSVPLMVAFTGRTTPADLITCGLAGLAGFAAFYWINRLGSIRFLSEFMGAFIIGLITLLGFYLTNGHYHPDAIIIGAVMPLVPGVAITNAVRDTMTGNLLSGPARAVEAVLSACAIGVGIALTSFFY	250	> CAMPSQ1461<br>Length=44<br>Score = 23.5 bits (49), Expect = 4.3<br>Identities = 10/32 (31%), Positives = 20/32 (63%), Gaps = 0/32 (0%)<br>Query 212 NAVRDTMTGNLLSGPARAVEAVLSACAIGVGI 243<br>NA++DT+T + + A+ +++S GVG+<br>Sbjct 6 NAIKDTVTAAINNDGAKLGTSIVSIVENGVGL 37	Title : Antibacterial protein<br>Length : 44<br>Activity : Antimicrobial<br>Validated : Predicted<br>Sequence: MEGLFNAIKDTVTAAINNDGAKLGTSIVSIVENGVGLLGKLFGF
			> CAMPSQ813<br>Length=60<br>Score = 23.5 bits (49), Expect = 5.2<br>Identities = 14/47 (30%), Positives = 25/47 (53%), Gaps = 2/47 (4%)<br>Query 29 EDTMQRIVT--NAGYPDAQVFVLLTGITVSLPENATSEVRAIHNRGM 73<br>+ T +++V+ NAG + +L G+TV L T+ AI +G+<br>Sbjct 8 QGTAEKVVSLINAGLTVGSIISILGGVTVGLSGVFTAVKAAIAKQGI 54	Title : Carnocyclin A<br>Source : Carnobacterium maltaromaticum<br>Length : 60<br>Activity : Antibacterial<br>LVAYGIAQGTAEKVVSLINAGLTVGSIISILGGVTVGLSGVFTAVKAAIAKQGIKKAIQL

	CAMPSQ1461	Carnocyclin A
total hydrophobic ratio (%)	53	40
total net charge	0	-.75
MW (Da)	3127.645	5542.35
Secondary structure	helix	helix

Protein type	Signal peptide (Sec/SPI)	TAT signal peptide (Tat/SPI)	Lipoprotein signal Peptide (Sec/SPII)	Other
**Likelihood**	0.0528	0.0044	0.0077	0.9351

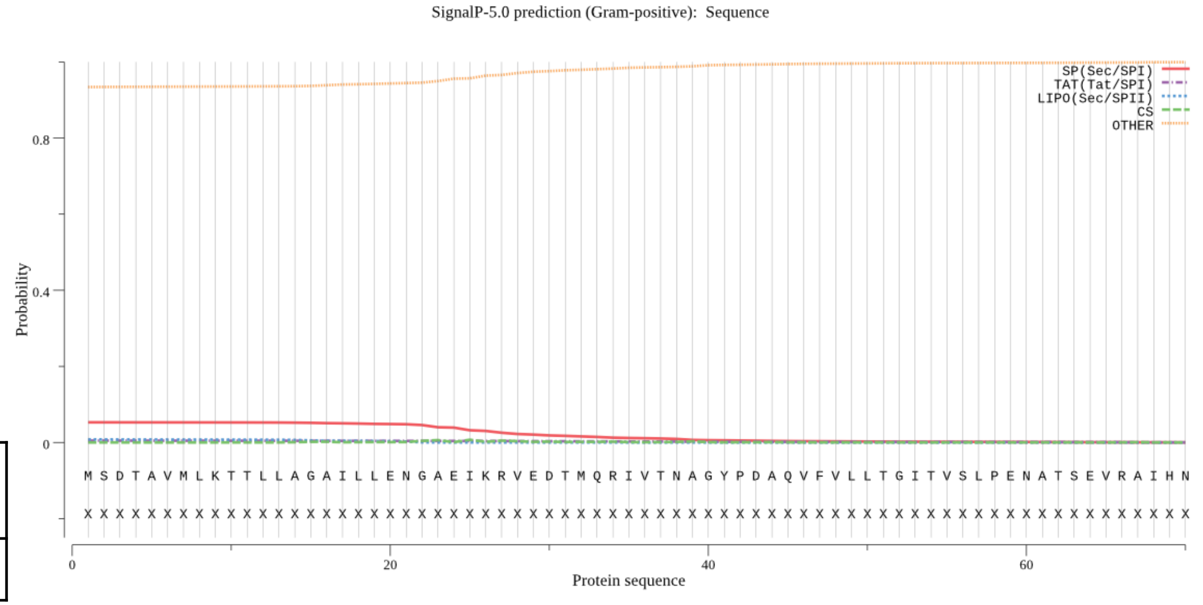

Suppl. Fig. S41. Prediction of signal sequence for LPK9 AMP15

contig 1

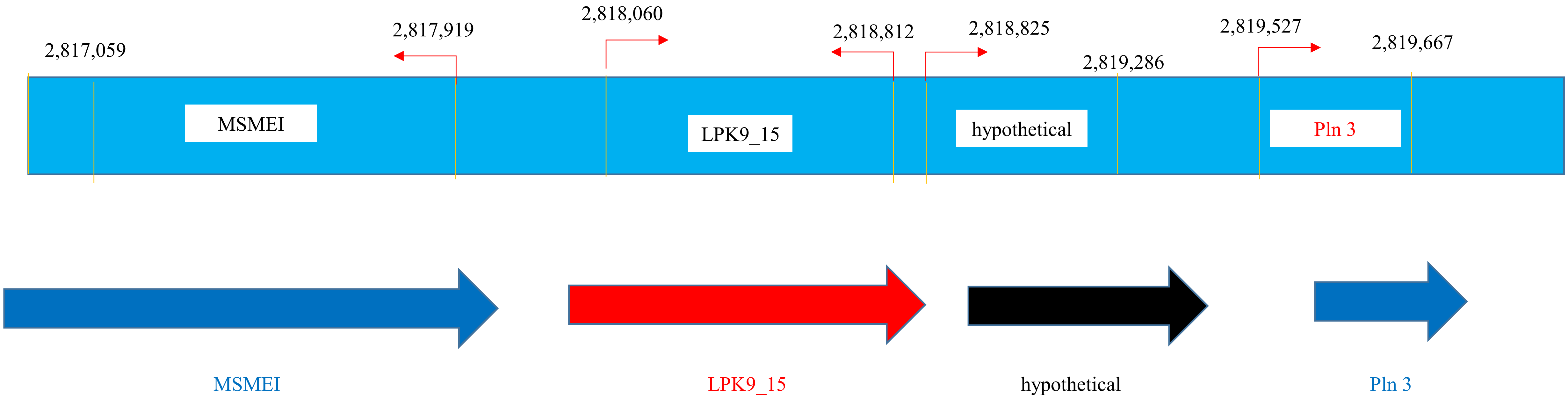

MSMEI; putative oxidoreductase/MSMEI_2347, pln 3; plantaricin 3

Suppl. Fig. S42. Prediction of signal sequence for LPK9 AMP16

Name	amino acid seq	amino acid No	campsign	Others
>JKLLNNAJ_02882 hypothetical protein	MHWLWVLIIGAIIGAIAGAITNKGKSMGWISNIIAGLVGSAIGEALLGSWGPQLAGMAIVPSIIGGVIVVAITSFVLTRMD	81	> CAMPSQ1102<br>Length=26<br>Score = 20.8 bits (42), Expect = 7.8<br>Identities = 9/21 (43%), Positives = 14/21 (67%), Gaps = 0/21 (0%)<br>Query 22 NKGKSMGWISNIIAGLVGSAI 42<br>N K +G I+ + AGL+GS +<br>Sbjct 1 NWRKILGKIAKVAAGLLGSML 21	Title : GrammistinGsC<br>Source : Grammistes sexlineatus [Sixline soapfish]<br>Length : 26<br>Activity : Antibacterial<br>NWRKILGKIAKVAAGLLGSMLAGYQV
			> CAMPSQ3254<br>Length=17<br>Score = 20.8 bits (42), Expect = 8.2<br>Identities = 7/16 (44%), Positives = 13/16 (81%), Gaps = 0/16 (0%)<br>Query 8 IIGAIIGAIAGAITNK 23<br>I+GAI+ ++G ++NK<br>Sbjct 1 ILGAILPLVSGLLSNK 16	Title : Alyteserin-2b<br>Source : Alytes obstetricans [Common midwife toad]<br>Length : 17<br>Activity : Antibacterial<br>Validated : Predicted<br>Sequence: ILGAILPLVSGLLSNKL

	Grammistin GsC	Alyteserin-2b
total hydrophobic ratio (%)	47	62
total net charge	+2	+1
MW (Da)	2116.508	1495.826
Secondary structure	helix	helix

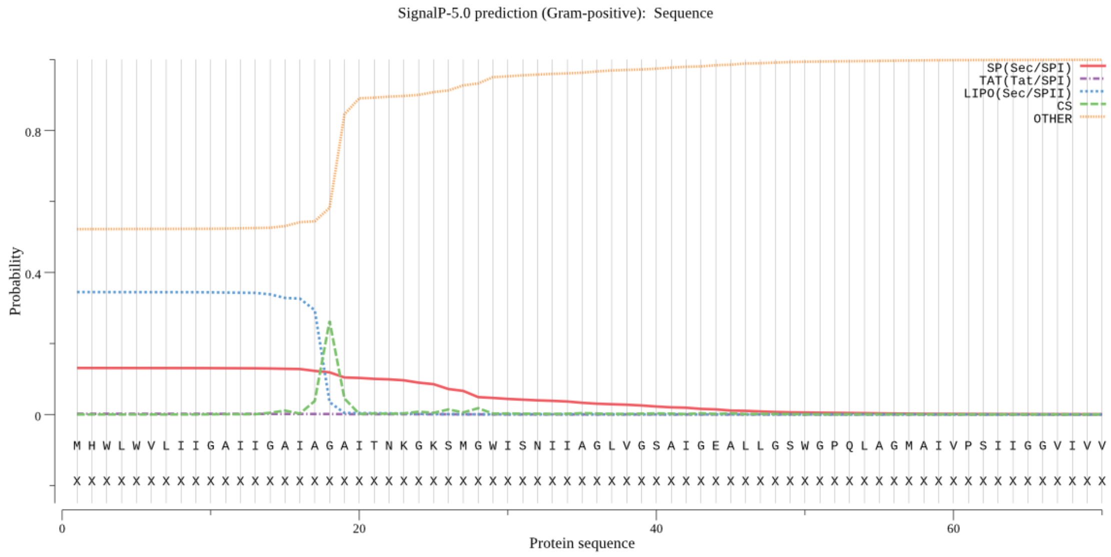

Protein type	Signal peptide (Sec/SPI)	TAT signal peptide (Tat/SPI)	Lipoprotein signal Peptide (Sec/SPII)	Other
**Likelihood**	0.1295	0.0021	0.3427	0.5256

Suppl. Fig. S43. Prediction of signal sequence for LPK9 AMP16

Name	amino acid seq	amino acid No	campsign	Others
>JKLLNNAJ_02882 hypothetical protein	MHWLWVLIIGAIIGAIAGAITNKGKSMGWISNIIAGLVGSAIGEALLGSWGPQLAGMAIVPSIIGGVIVVAITSFVLTRMD	81	> CAMPSQ1102, Length=26<br>Score = 20.8 bits (42), Expect = 7.8<br>Identities = 9/21 (43%), Positives = 14/21 (67%), Gaps = 0/21 (0%)<br>Query 22 NKGKSMGWISNIIAGLVGSAI 42<br>N K +G I+ + AGL+GS +<br>Sbjct 1 NWRKILGKIAKVAAGLLGSML 21	Title : GrammistinGsC<br>Source : Grammistes sexlineatus [Sixline soapfish]<br>Length : 26<br>Activity : Antibacterial<br>NWRKILGKIAKVAAGLLGSMLAGYQV
			> CAMPSQ3254, Length=17<br>Score = 20.8 bits (42), Expect = 8.2<br>Identities = 7/16 (44%), Positives = 13/16 (81%), Gaps = 0/16 (0%)<br>Query 8 IIGAIIGAIAGAITNK 23<br>I+GAI+ ++G ++NK<br>Sbjct 1 ILGAILPLVSGLLSNK 16	Title : Alyteserin-2b<br>Source : Alytes obstetricans [Common midwife toad]<br>Length : 17<br>Activity : Antibacterial<br>Validated : Predicted<br>Sequence: ILGAILPLVSGLLSNKL

Suppl. Fig. S44. Prediction of signal sequence for LPK9 AMP16

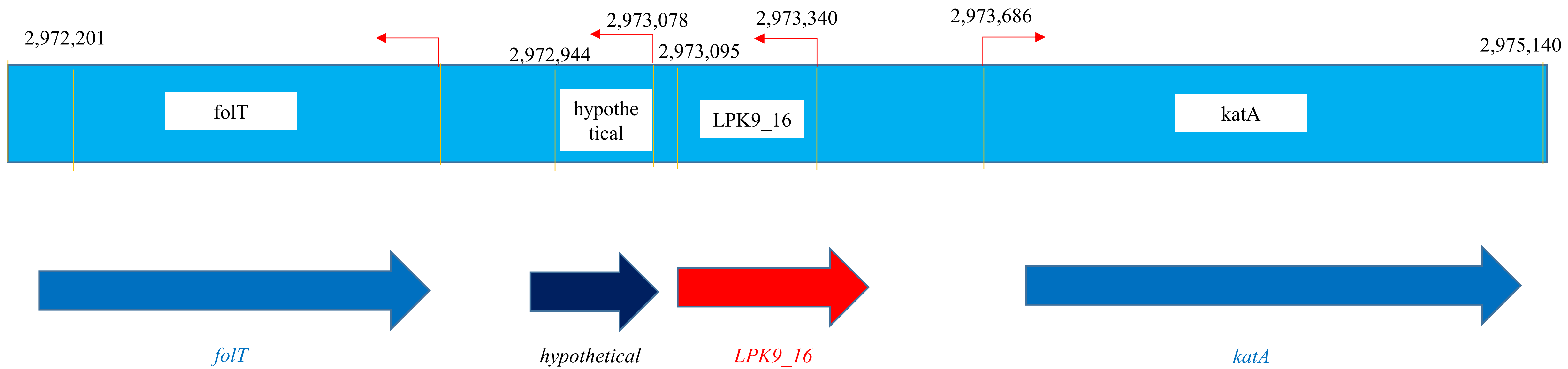

folT; Folate transporter FolT, katA; Vegetative catalase

Suppl. Fig. S45. Prediction of signal sequence for LPK9 AMP17

Name	amino acid seq	amino acid No	campsign	Others
>INGLFCCM_00010<br>hypothetical protein	MHWLWVLIIGAIIGAIAGAITSKGKSMGWI<br>ANIVAGLVGSSIGEAILGSWGPQLAGMAII<br>PSIIGAVIVVAVVSFFLSRSTR	82	> CAMPSQ1102 Length=26 Score = 21.9 bits (45), Expect = 3.0<br>Identities = 9/18 (50%), Positives = 13/18 (72%), Gaps = 0/18 (0%)<br>Query 25 KSMGWIANIVAGLVGSSI 42<br>K +G IA + AGL+GS +<br>Sbjct 4 KILGKIAKVAAGLLGSML 21	Title : GrammistinGsC<br>Source : Grammistes sexlineatus [Sixline soapfish]<br>Length : 26<br>Activity : Antibacterial<br>NWRKILGKIAKVAAGLLGSMLAGYQV
			> CAMPSQ2899 Length=31 Score = 21.9 bits (45), Expect = 3.1<br>Identities = 9/26 (35%), Positives = 16/26 (62%), Gaps = 0/26 (0%)<br>Query 9 IGAIIGAIAGAITSKGKSMGWIANIV 34<br>+G+I+G I GK++G +A+ V<br>Sbjct 2 LGSILGKILNVAGKVGKTIGKVADAV 27	Title : Caerulein precursor-related fragment Ea<br>Source : Rana esculenta [Edible frog]<br>Length : 31<br>Activity : Antibacterial<br>Validated : Experimentally Validated<br>Sequence: GLGSILGKILNVAGKVGKTIGKVADAVGNKE
			> CAMPSQ1026 Length=30 Score = 21.6 bits (44), Expect = 4.2<br>Identities = 8/24 (33%), Positives = 15/24 (63%), Gaps = 0/24 (0%)<br>Query 12 IIGAIAGAITSKGKSMGWIANIVA 35<br>I A+AG + + ++ GW+ IV+<br>Sbjct 6 IPAAVAGTVLNVVEAGGWVTTIVS 29	Title : Bacteriocin As-48<br>Source : Enterococcus faecalis<br>Length : 30<br>Activity : Antibacterial<br>Validated : Experimentally Validated<br>Sequence: AKEFGIPAAVAGTVLNVVEAGGWVTTIVSI
			> DBAASP_1407|1537|SIn2-3 Length=46 Score = 21.6 bits (44), Expect = 5.4<br>Identities = 11/35 (31%), Positives = 18/35 (51%), Gaps = 1/35 (3%)<br>Query 24 GKSMGWIANIVAGLVGSSIGEAILGSWGPQLAGMA 58<br>G W+ + AG+VG ++ AI G W G++<br>Sbjct 6 GSGNHWV-HCGAGIVGGALIGAIGGPWSAVAGGIS 39	Name : SIn2-3<br>Synthesis Type : Synthetic<br>Target Group :<br>Target Object :<br>UniProt : Probable Precursor: V6DN13<br>HNGYGGSGNHWVHCGAGIVGGALIGAIGGPWSAVAGGISGGFASCH
			> CAMPSQ1094 Length=25 Score = 21.2 bits (43), Expect = 5.8<br>Identities = 9/16 (56%), Positives = 13/16 (81%), Gaps = 0/16 (0%)<br>Query 25 KSMGWIANIVAGLVGS 40<br>K +G IA++ AGL+GS<br>Sbjct 4 KILGQIASVGAGLLGS 19	CAMPSQ1094<br>Title : Grammistin Pp3<br>Source : Pogonoperca punctata [Clown grouper]<br>Length : 25<br>Activity : Antibacterial<br>Hemolytic Activity : Has hemolytic activity<br>Validated : Experimentally Validated<br>NWRKILGQIASVGAGLLGSLLAGYE

Suppl. Fig. S46. Prediction of signal sequence for LPK9 AMP17

	: GrammistinGsC	Caerulein precursor-related fragment Ea	Bacteriocin As-4	: Sln2-3	Grammistin Pp3
total hydrophobic ratio (%)	55	57	58	51	56
total net charge	+1	+2	+2	0	+1
MW (Da)	1803.155	2513.04	2342.828	3399.986	1602.917
Secondary structure	helix	helix	helix	helix	helix

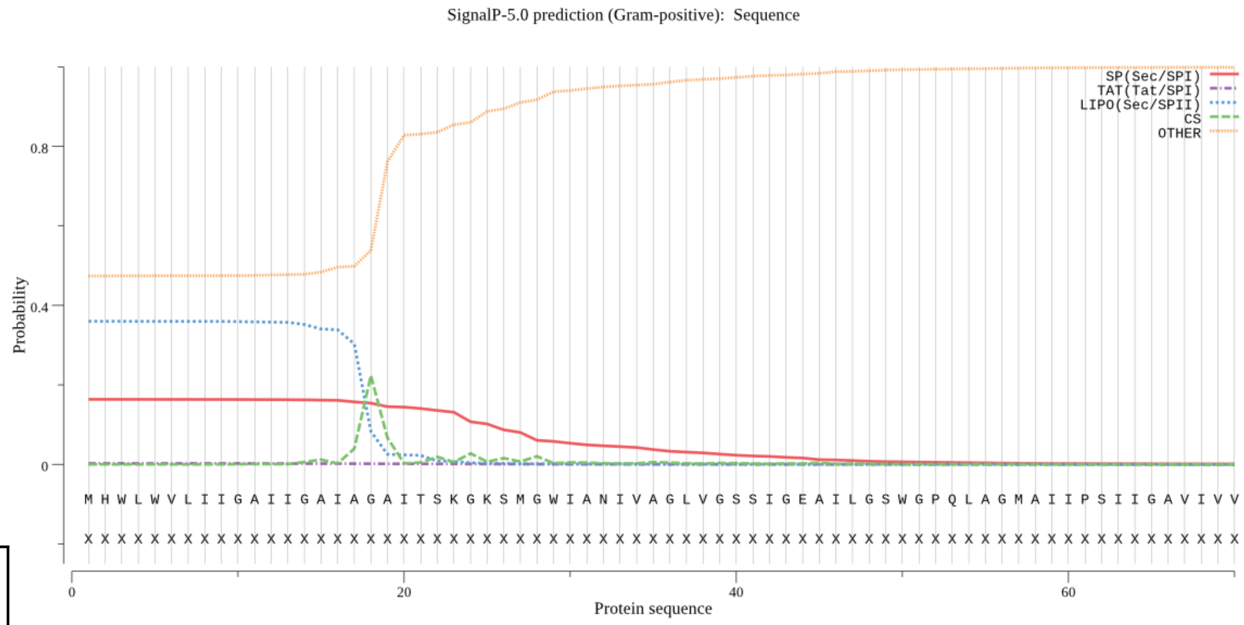

Protein type	Signal peptide (Sec/SPI)	TAT signal peptide (Tat/SPI)	Lipoprotein signal Peptide (Sec/SPII)	Other
**Likelihood**	0.1615	0.0025	0.3596	0.4764

Suppl. Fig. S47. Prediction of signal sequence for LPK9 AMP17

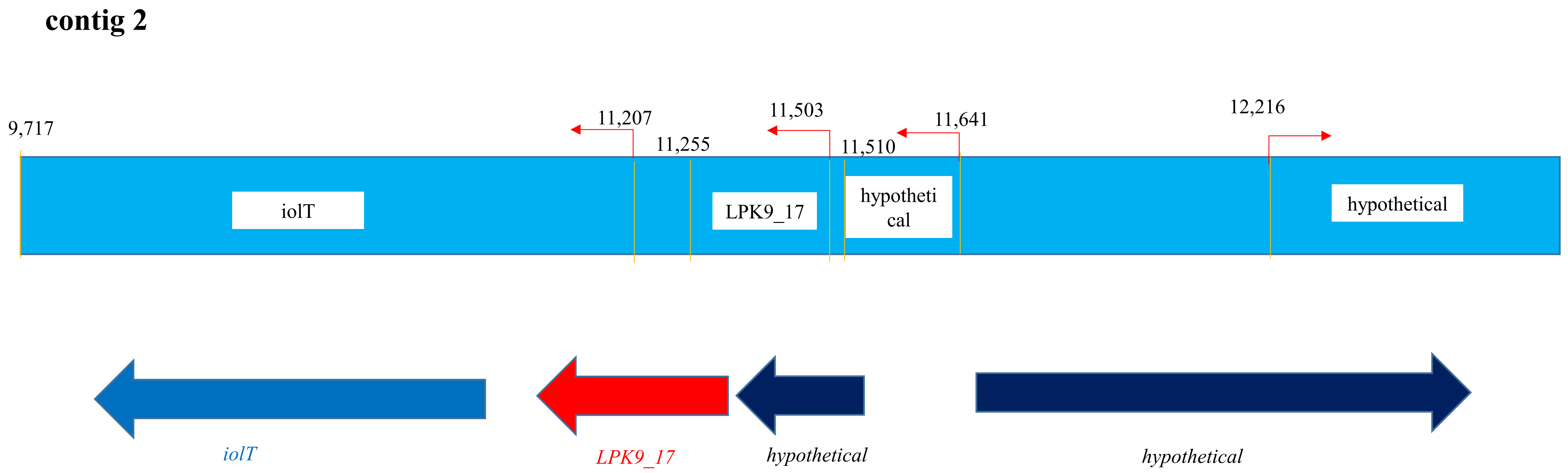

iolT; Major myo-inositol transporter IolT

Suppl. Fig. S48. Prediction of signal sequence for LPK9 AMP18

Name	amino acid seq	amino acid No	campsign	Others
>MLOLCKOO_00006 hypothetical protein	MSELKRYLIVAGINGAGKSTLYRARPELFTHSKRLNADEILQKMGGDWRKDRDNFRAMREEIKQFQRL	68	> CAMPSQ4328<br>Length=24<br>Score = 20.4 bits (41), Expect = 7.8<br>Identities = 6/14 (43%), Positives = 12/14 (86%), Gaps = 0/14 (0%)<br>Query 52 RDNFRAMREEIKQF 65<br>+D F++M E++KQ+<br>Sbjct 3 KDKFKSMGEKLKQY 16	Title : Latarcin 4a<br>Source : Lachesana tarabaevi [Spider]<br>Length : 24<br>Activity : Antibacterial, Antifungal<br>GLKDKFKSMGEKLKQYIQTWKAKF

total hydrophobic ratio (%)	35
total net charge	+1
MW (Da)	1840.097
Secondary structure	helix

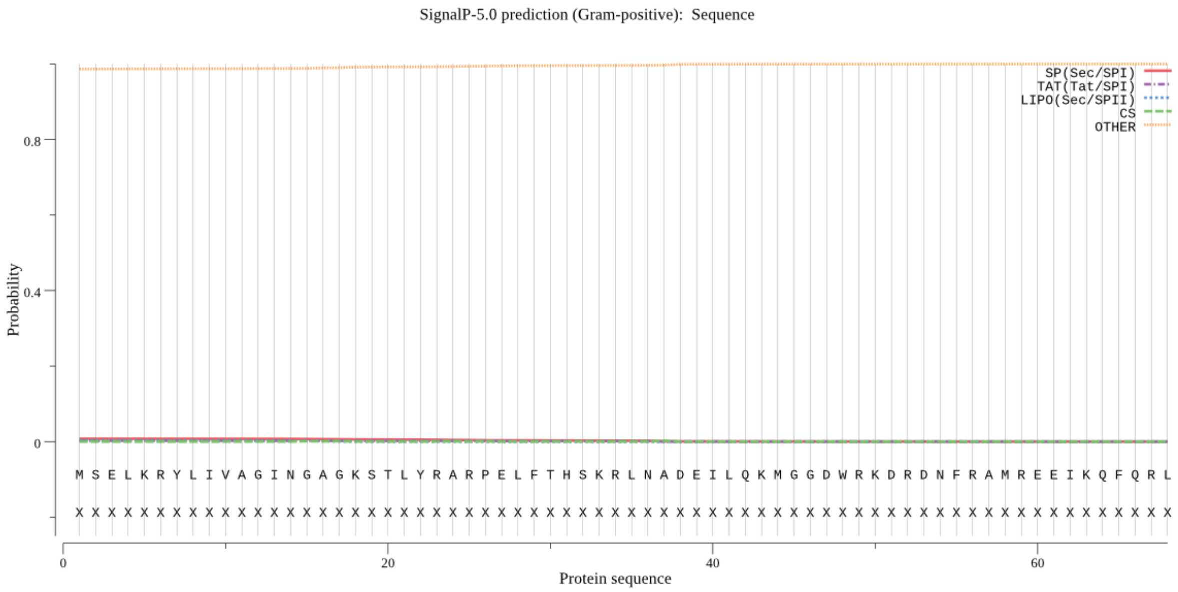

Protein type	Signal peptide (Sec/SPI)	TAT signal peptide (Tat/SPI)	Lipoprotein signal Peptide (Sec/SPII)	Other
**Likelihood**	0.0071	0.002	0.0029	0.9881

Suppl. Fig. S49. Prediction of signal sequence for LPK9 AMP18

contig 3

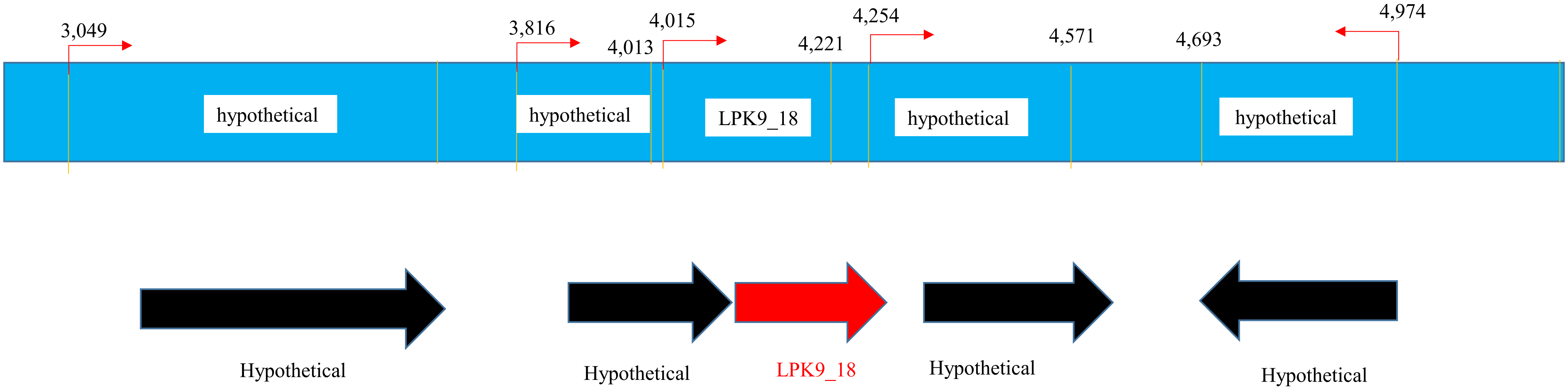

Suppl. Fig. S50. contig 1

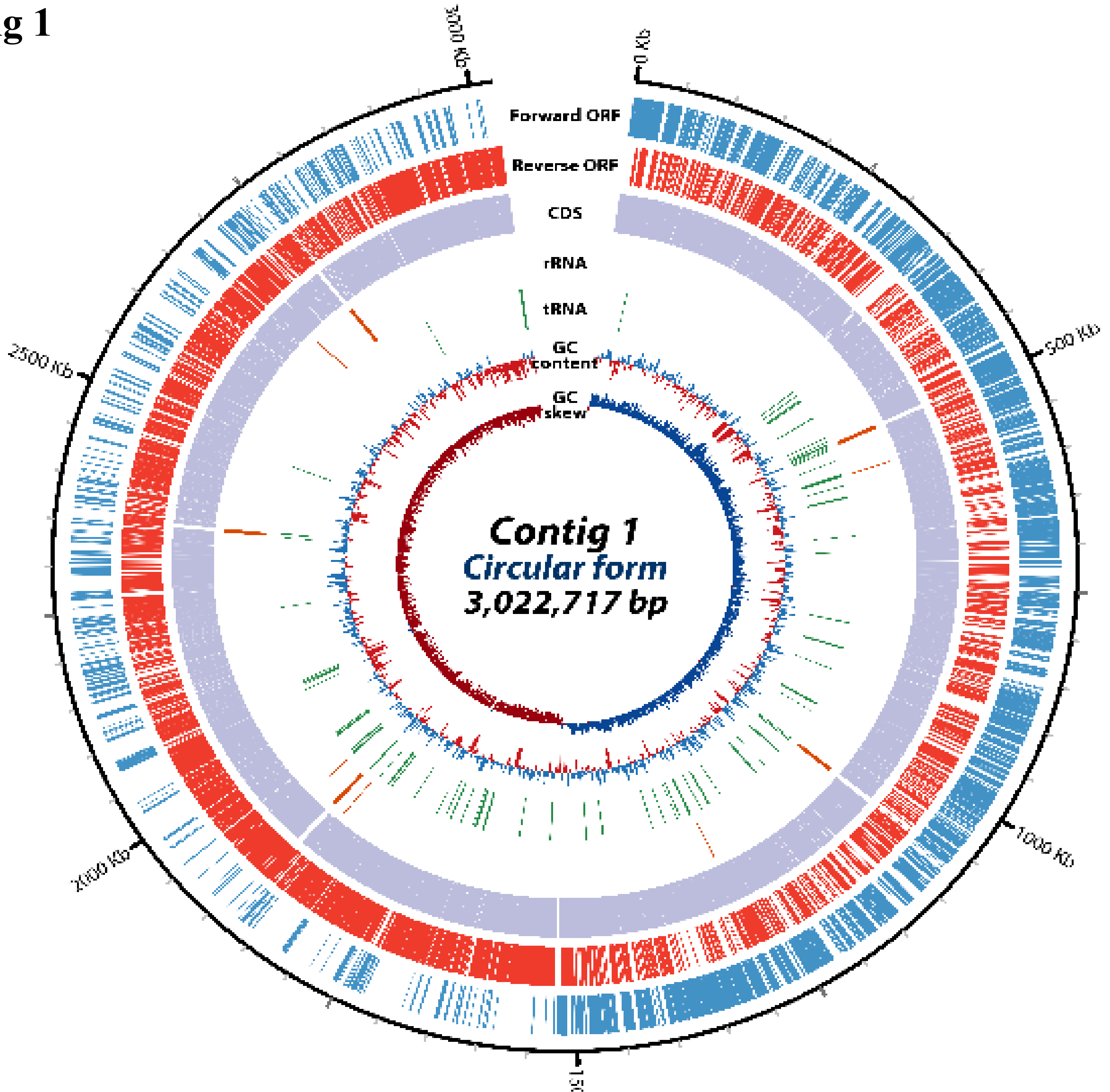

Fig. 2A

Suppl. Fig. S51. contig 2

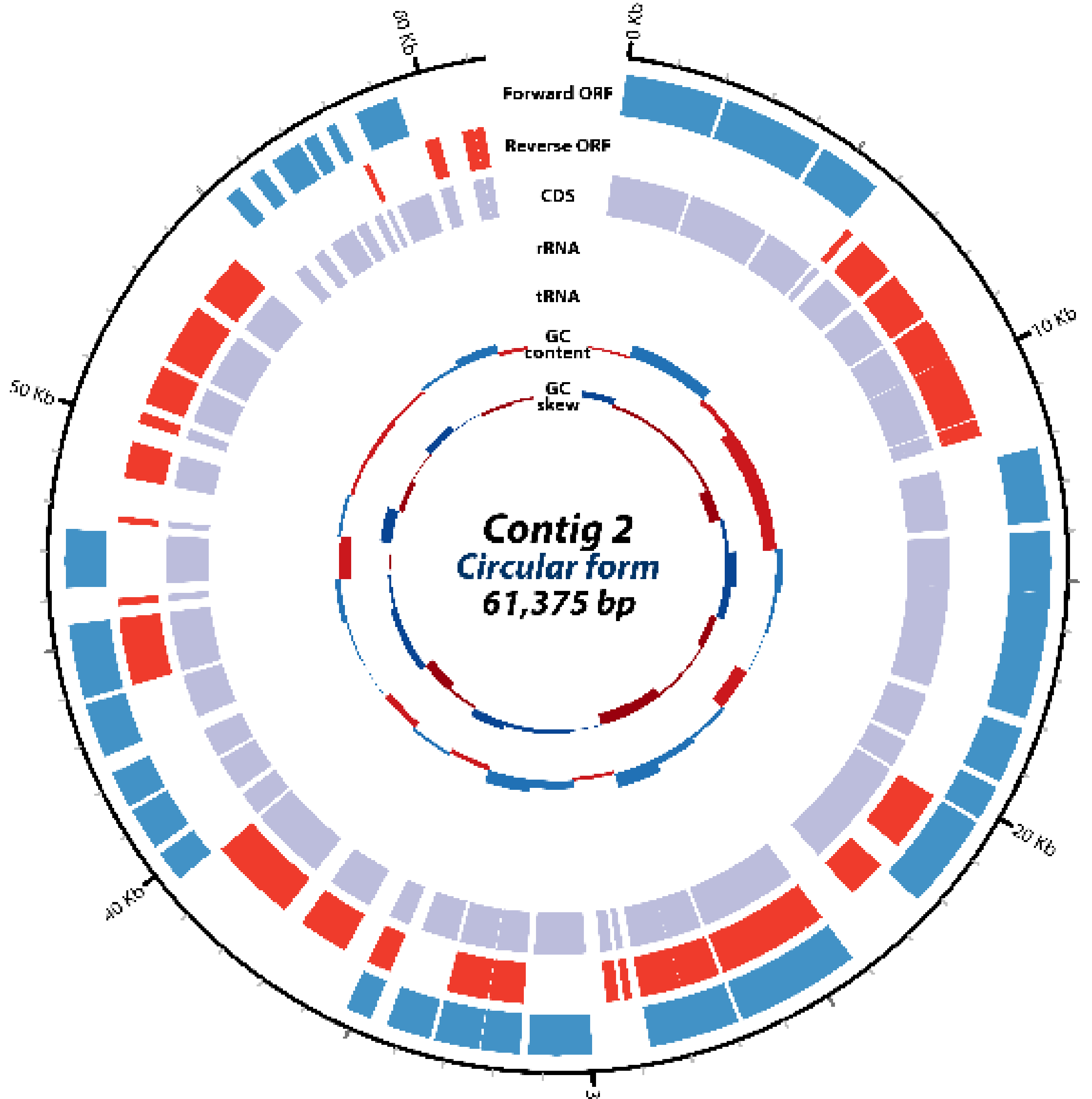

Suppl. Fig. S52. contig 3

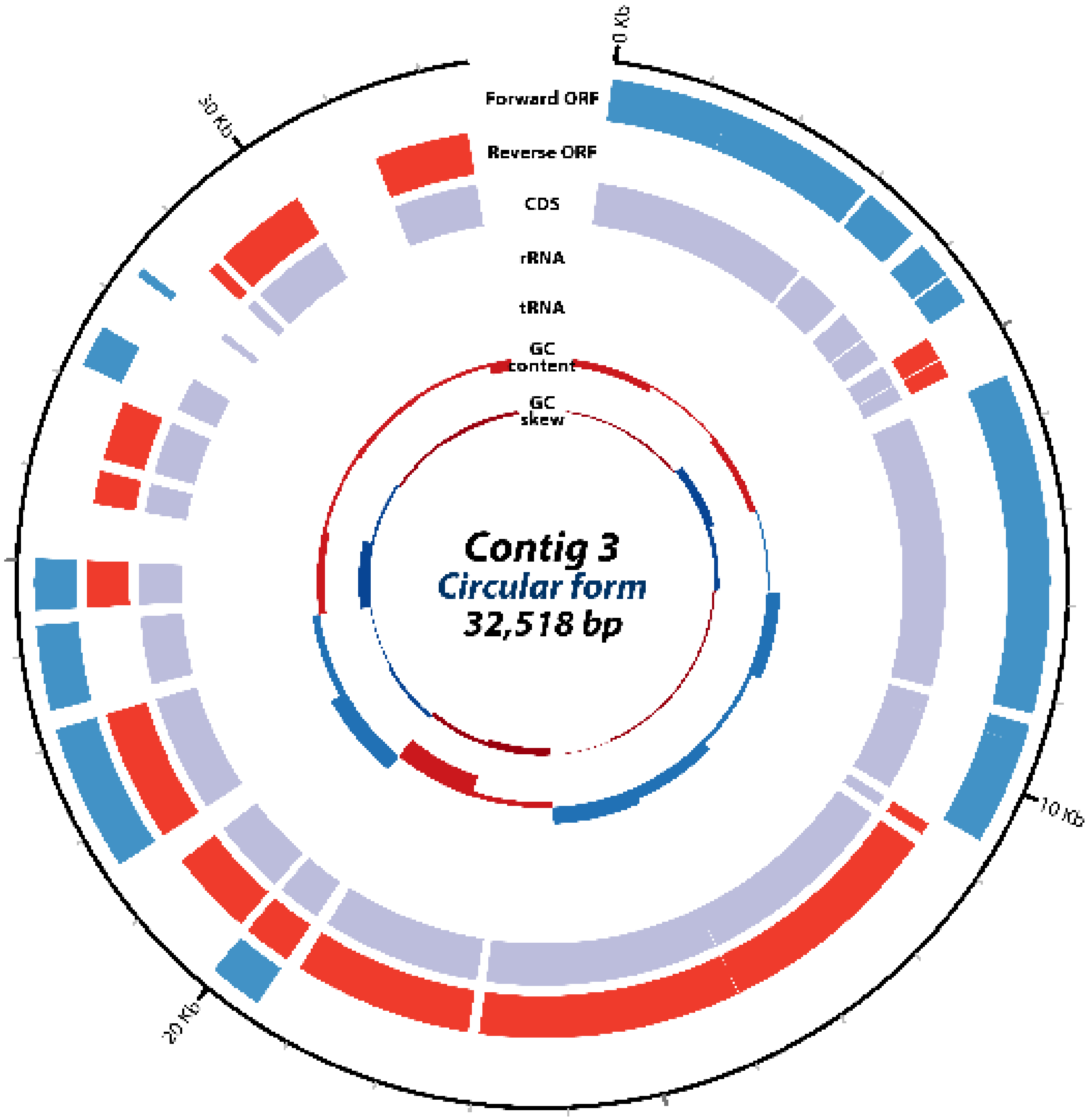

Suppl. Fig. S53. contig 4

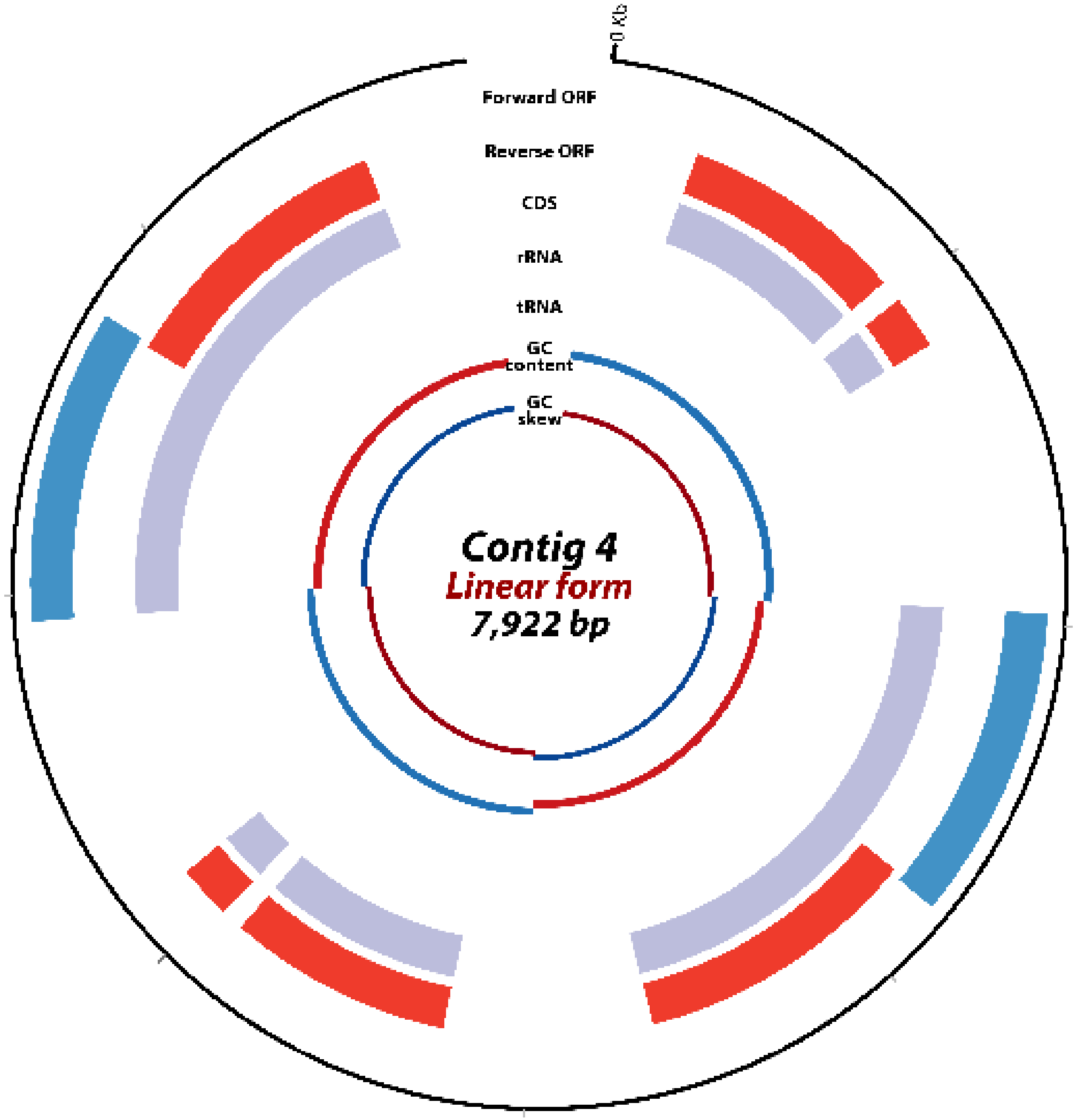

Supplement: Supplementary file 1 [file ijms-27-06843-s001.zip › ijms-4450605-supplementary.pdf]
